# Predicting Carriers of Ongoing Selective Sweeps without Knowledge of the Favored Allele

**DOI:** 10.1371/journal.pgen.1005527

**Published:** 2015-09-24

**Authors:** Roy Ronen, Glenn Tesler, Ali Akbari, Shay Zakov, Noah A. Rosenberg, Vineet Bafna

**Affiliations:** 1 Bioinformatics Graduate Program, University of California, San Diego, La Jolla, California, United States of America; 2 Department of Mathematics, University of California, San Diego, La Jolla, California, United States of America; 3 Department of Electrical & Computer Engineering, University of California, San Diego, La Jolla, California, United States of America; 4 Department of Computer Science & Engineering, University of California, San Diego, La Jolla, California, United States of America; 5 Department of Biology, Stanford University, Stanford, California, United States of America; University of California Davis, UNITED STATES

## Abstract

Methods for detecting the genomic signatures of natural selection have been heavily studied, and they have been successful in identifying many selective sweeps. For most of these sweeps, the favored allele remains unknown, making it difficult to distinguish carriers of the sweep from non-carriers. In an ongoing selective sweep, carriers of the favored allele are likely to contain a future most recent common ancestor. Therefore, identifying them may prove useful in predicting the evolutionary trajectory—for example, in contexts involving drug-resistant pathogen strains or cancer subclones. The main contribution of this paper is the development and analysis of a new statistic, the Haplotype Allele Frequency (HAF) score. The HAF score, assigned to individual haplotypes in a sample, naturally captures many of the properties shared by haplotypes carrying a favored allele. We provide a theoretical framework for computing expected HAF scores under different evolutionary scenarios, and we validate the theoretical predictions with simulations. As an application of HAF score computations, we develop an algorithm (PreCIOSS: Predicting Carriers of Ongoing Selective Sweeps) to identify carriers of the favored allele in selective sweeps, and we demonstrate its power on simulations of both hard and soft sweeps, as well as on data from well-known sweeps in human populations.

## Author Summary

Methods for detecting the genomic signatures of natural selection have been heavily studied, and they have been successful in identifying genomic regions under positive selection. However, methods that detect positive selective sweeps do not typically identify the favored allele, or even the haplotypes carrying the favored allele. The main contribution of this paper is the development and analysis of a new statistic (the HAF score), assigned to individual haplotypes. Using both theoretical analyses and simulations, we describe how the HAF scores differ for carriers and non-carriers of the favored allele, and how they change dynamically during a selective sweep. We also develop an algorithm, PreCIOSS, for separating carriers and non-carriers. Our tool has broad applicability as carriers of the favored allele are likely to contain a future most recent common ancestor. Therefore, identifying them may prove useful in predicting the evolutionary trajectory—for example, in contexts involving drug-resistant pathogen strains or cancer subclones.

## Introduction

With genome sequencing, we now have an opportunity to more completely sample genetic diversity in human populations, and probe deeper for signatures of adaptive evolution [1–3]. Genetic data from diverse human populations in recent years have revealed a multitude of genomic regions believed to be evolving under recent positive selection [4–16].

Methods for detecting selective sweeps from DNA sequences have examined a variety of signatures, including patterns represented in variant allele frequencies as well as in haplotype structure. Initially, the problem of detecting selective sweeps was approached primarily by considering variant allele frequencies, exploiting the shift in frequency at ‘hitchhiking’ sites linked to a favored allele relative to non-hitchhiking sites [17, 18]. The site frequency spectrum (SFS) within and across populations is often used as a basis for such inference [4, 6, 19–25]. More recently, methods based on haplotype structure have been developed using a variety of approaches, including the frequency of the most common haplotype [26], the number and diversity of distinct haplotypes [27], the haplotype frequency spectrum [28], and the popular approach of long-range haplotype homozygosity [29–32].

In general, haplotype-based methods seek to characterize the population with summary statistics that capture the frequency and length of different haplotypes. However, the haplotypes are related through a genealogy, and relationships among them are inherently lost in such analyses. In addition, data on the site frequency spectrum can be lost or hidden in analyses focused on haplotype spectra. In this paper, we connect related measures of haplotype frequencies and the site frequency spectrum by merging information describing haplotype relationships with variant allele frequencies. Our main contribution is a statistic that we term the *haplotype allele frequency* (HAF) score, which captures many of the properties shared by haplotypes carrying a favored allele.

**Fig 1:**
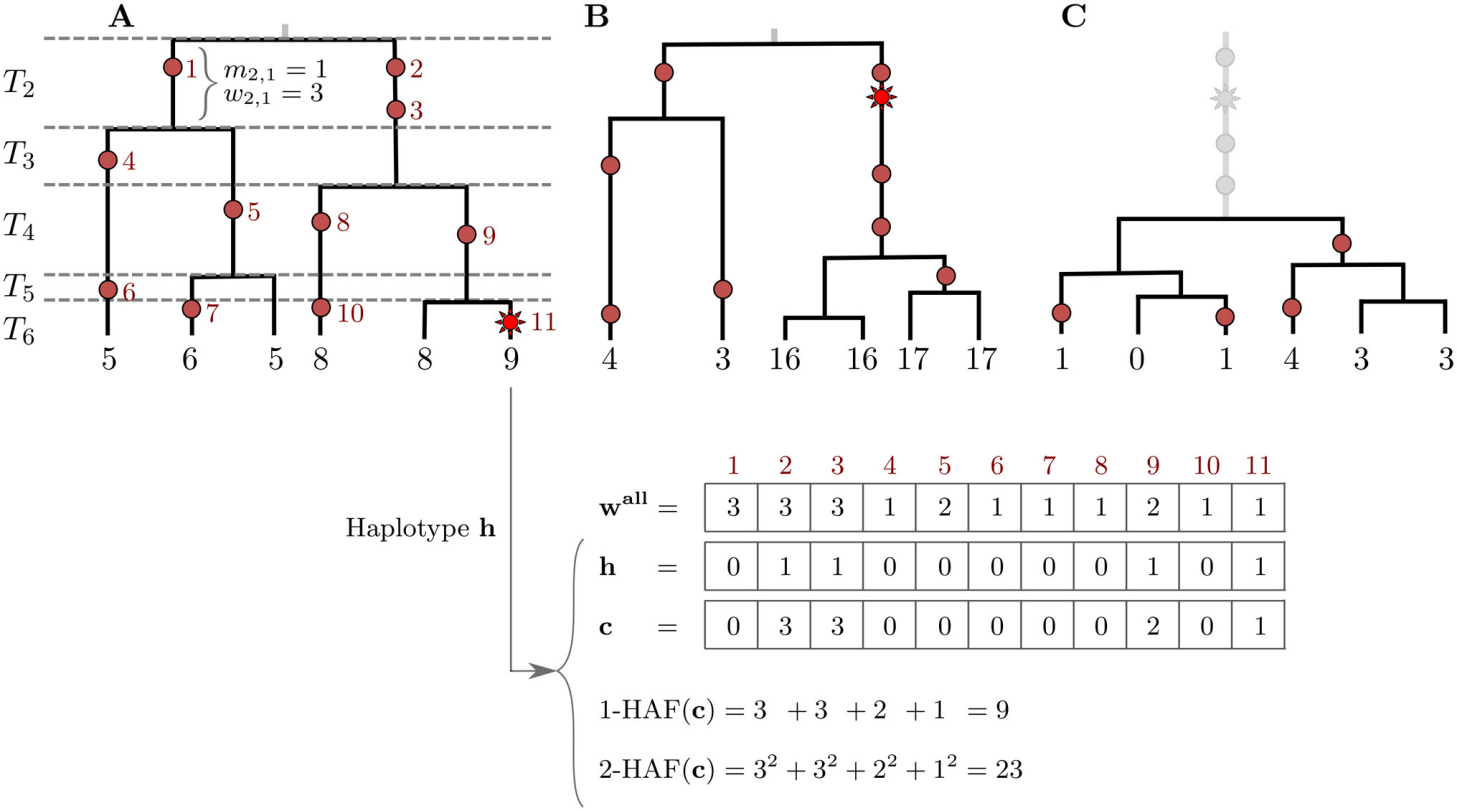
The HAF score. Genealogies of three samples (*n* = 6) progressing through a selective sweep, from left to right. Neutral mutations are shown as red circles, and are numbered in red; the favored allele is shown as a red star. The 1-HAF score of each haplotype is shown below its corresponding leaf, in black. For the rightmost haplotype in (A), the binary haplotype vector **h** is shown along with its HAF-vector **c**, and 1-HAF and 2-HAF scores. Vector **w** ^all^ lists the frequencies of all mutations. (A) The favored allele appears on a single haplotype. At this point in time, both the genealogy and the HAF score distribution are largely neutral. Coalescence times (*T*_2_, …, *T*_6_) are shown on the left, where *T*_*k*_ spans the epoch with exactly *k* lineages. (B) Carriers of the favored allele are distinguished by high HAF scores (in large part due to the long branch of high-frequency hitchhiking variation); non-carriers have low HAF scores. (C) After fixation, there is a sharp loss of diversity causing low HAF scores across the sample.

Consider a sample of haplotypes in a genomic region. We assume that all sites are biallelic, and at each site, we denote ancestral alleles by 0 and derived alleles by 1. We also assume that all sites are polymorphic in the sample. The *HAF vector* of a haplotype *h*, denoted **c**, is obtained by taking the binary haplotype vector and replacing non-zero entries (derived alleles carried by the haplotype) with their respective frequencies in the sample (Fig 1A). For parameter ℓ, we define the ℓ-HAF *score* of **c** as:

$$\ell \text{-HAF}\left(c\right)=\sum_{j}{c_{j}}^{\ell }$$ (1)

where the sum proceeds over all segregating sites *j* in the genomic region. The 1-HAF score of a haplotype amounts to the sum of frequencies of all derived alleles carried by the haplotype. The ℓ-HAF score is equivalent to the ℓ-norm of **c** raised to the ℓ^th^ power, or ${\left({\left\|c\right\|}_{\ell }\right)}^{\ell }$. We will show that during a selective sweep, the HAF score of a haplotype serves as a proxy to its relative fitness.

### Selective sweeps

The classical model for selection, and the one that has received most attention, is the “hard sweep” model, in which a single mutation conveys higher fitness immediately upon occurrence, and rapidly rises in frequency, eventually reaching fixation [17, 33]. Under this model, we can partition the haplotypes into carriers of the favored allele, and non-carriers. In the absence of recombination, the favored haplotypes form a single clade in the genealogy. As a sweep progresses, HAF scores in the favored clade will rise due to the increasing frequencies of alleles hitchhiking along with the favored allele. HAF scores of non-carrier haplotypes will decrease, as many of the derived alleles they carry become rare (Fig 1B). After fixation of the favored and hitchhiking alleles, HAF scores will decline sharply (Fig 1C), as the selected site and other linked sites are no longer polymorphic. Thus, this reduction in the HAF score results from the sudden loss of many high-frequency derived alleles from the pool of segregating sites [18, 20, 24]. Finally, as the site-frequency spectrum recovers to its neutral state due to new mutations and drift [23], so will the HAF scores.

Recombination is a source of ‘noise’ for the properties of the HAF score, predicted under the assumption of a hard sweep and no recombination, as it allows haplotypes to cross *into* and *out of* the favored clade. Recombination can lead to (i) haplotypes that carry the favored allele but little of the hitchhiking variation, thus having relatively low HAF scores despite their high fitness, or (ii) haplotypes that do not carry the favored allele but do carry much of the hitchhiking variation, thus having relatively high HAF scores despite their low fitness. By the same logic, recombination adds ‘noise’ after fixation by making the otherwise sharp decline in HAF scores more subtle and gradual. This more gradual decline occurs due to recombination weakening the linkage between the favored allele and hitchhiking variants.

Recently, “soft sweeps” have generated significant interest [34–36]. A soft sweep occurs when multiple sets of hitchhiking alleles in a given region increase in frequency, rather than a single favored haplotype. Soft sweeps may take place by one or more of the following mechanisms: (i) selection from standing variation: a neutral segregating mutation, which exists on several haplotypic backgrounds, becomes favored due to a change in the environment; (ii) recurrent mutation: the favored mutation arises several times on different haplotypic backgrounds; or, (iii) multiple adaptations: multiple favored mutations occur on multiple haplotypic backgrounds. Several methods have been developed for detecting soft sweeps [37, 38], as well as for distinguishing between soft and hard sweeps [39–41]. In soft sweeps, multiple sets of hitchhiking alleles rise to intermediate frequencies as the favored allele fixes. This could cause the pre-fixation peak and post-fixation trough in HAF scores to be less pronounced and to occur more gradually compared to a hard sweep.

We find (see Results) that the properties of the HAF score remain robust to many soft sweep scenarios. Moreover, the HAF score could potentially be used to detect soft sweeps. However, in this paper, we focus on the foundations, developing theoretical analysis and empirical work that predicts the dynamics of the HAF score. We also develop a single application. Recall that most existing methods for characterizing selective sweeps focus on identifying regions under selection. Here, given a region already identified to be undergoing a selective sweep, we ask if we can accurately predict which haplotypes carry the favored allele, without knowledge of the favored site. Successfully doing so may provide insight into the future evolutionary trajectory of a population. Haplotypes in future generations are more likely to be descended from, and therefore to resemble, extant carriers of a favored allele. This predictive perspective is of particular importance when a sweep is undesirable and measures may be taken to prevent it. For instance, consider a set of tumor haplotypes isolated from single cells, some of which are drug-resistant and therefore favored under drug exposure. Given a genetic sample of the tumor haplotypes, the HAF statistic may be applied to identify the resistant haplotypes—carriers of a favored allele—before they clonally expand and metastasize.

Below, we start with a theoretical explanation of the behavior of the HAF score under different evolutionary scenarios, validating our results using simulation. We then develop an algorithm (PreCIOSS: Predicting Carriers of Ongoing Selective Sweeps) to detect carriers of selective sweeps based on the HAF score. We demonstrate the power of PreCIOSS on simulations of both hard and soft sweeps, as well as on real genetic data from well-known sweeps in human populations. While our theoretical derivations make use of coalescent theory, and explicitly use tree-like genealogies, we note that HAF scores can be computed for any haplotype matrix including those with recombination events. Our results on simulated and real data imply that the utility of the HAF score extends to cases with recombination as well as other evolutionary scenarios.

## Results

### Theoretical and empirical modeling of HAF scores

We consider a sample of *n* haploid individuals chosen at random from a larger haploid population of size *N*. Let *μ* denote the mutation rate per generation per nucleotide, and let *θ* = 2*NμL* denote the population-scaled mutation rate in a region of length *L* bp. We consider both constant-sized and exponentially growing populations. For exponentially growing populations, let *N*_0_ denote the final population size, let *r* denote the growth rate per generation, and let *α* = 2 *N*_0_ *r* the population-scaled growth rate. Let *ρ* denote the population-scaled recombination rate. In our theoretical calculations, we assume no recombination (*ρ* = 0), and we derive expressions for the general ℓ-HAF score. We use simulations to demonstrate the concordance of theoretical and empirical values of the ℓ-HAF score, and show that the values are robust to the presence of recombination (see ‘Simulations’ in Methods for parameter choices). Although some of our theoretical calculations below derive expressions for the general ℓ-HAF score, we primarily use 1-HAF in the applied sections. Applications of ℓ-HAF with ℓ > 1 will be explored in future work.

#### Expected ℓ-HAF score under neutrality, constant population size

First, we assume that the genomic region of interest is evolving neutrally, the population size remains constant at *N*, and that the ancestral states are known or can be derived. In a sample of size *n*, let **c**(*v*) denote the HAF vector **c** for the *v* ^th^ haplotype (*v* ∈ {1, …, *n*}). Let *ξ*_*w*_ be the number of sites with derived allele frequency *w*. We only consider polymorphic sites in the sample, so the frequency is in the range *w* ∈ {1, …, *n* − 1}; a mutation present in all or none of the haplotypes in the sample would not be detectable. Each of the *ξ*_*w*_ sites of frequency *w* contributes *w* ^ℓ^ to the ℓ-HAF score of each of the *w* haplotypes with the mutation, and contributes 0^ℓ^ = 0 for each of the other *n* − *w* haplotypes. The mean of the ℓ-HAF scores of all *n* haplotypes in the sample is

$$\frac{1}{n}\sum_{v=1}^{n}\ell \text{-HAF}\left(c\left(v\right)\right)=\frac{1}{n}\sum_{w=1}^{n-1}\xi_{w}\cdot w^{\ell }\cdot w=\frac{1}{n}\sum_{w=1}^{n-1}\xi_{w}\cdot w^{\ell +1}.$$ (2)

Under the coalescent model, [42, Eq. (22)] shows that 𝔼[*ξ*_*w*_] = *θ*/*w* for all 1 ≤ *w* ≤ *n* − 1. By averaging over all haplotypes in all genealogies, the expected ℓ-HAF score is computed as

$$𝔼\left[\ell \text{-HAF}\right]=\frac{1}{n}\sum_{w=1}^{n-1}𝔼\left[\xi_{w}\right]\cdot w^{\ell }\cdot w=\frac{\theta }{n}\sum_{w=1}^{n-1}w^{\ell }\;.$$ (3)

The first two cases (ℓ = 1,2) yield

$$𝔼\left[1\text{-HAF}\right]=\frac{\theta (n-1)}{2}\;,\;\;𝔼\left[2\text{-HAF}\right]=\frac{\theta (n-1)(2n-1)}{6}\;.$$ (4)

#### Expected ℓ-HAF score, variable population size

Our derivation of expected ℓ-HAF scores for constant, neutrally evolving populations does not immediately extend to other demographic scenarios. We describe a second approach that separates coalescence times from the genealogy, and we apply it to compute the expected ℓ-HAF in an exponentially growing population.

For a sample of size *n*, partition the time spanning from the present back to the sample MRCA into *n* − 1 epochs. Let epoch *k* ∈ {2, …, *n*} be the span of time during which the genealogy contains exactly *k* lineages (Fig 1). Note that mutations on a given lineage in a given epoch share the same frequency, as they appear in exactly the same leaves. For example, mutations 2 and 3 in Fig 1A occur on the same lineage in epoch 2, and they share the frequency 3. Consider the path leading from a randomly chosen haplotype back to the sample MRCA. We can write the ℓ-HAF score of the haplotype as

$$\ell \text{-HAF}\left(c\right)=\sum_{k=2}^{n}m_{k}w_{k}^{\ell },$$ (5)

where *m*_*k*_ is the number of mutations that occurred on the path in the *k* ^th^ epoch, and *w*_*k*_ is the frequency or weight of those mutations. For a given genealogy with haplotypes *v* ∈ {1, …, *n*}, let **c**(*v*) denote **c** (the HAF vector) for the *v* ^th^ haplotype. Similarly, let *m*_*k*_(*v*) and *w*_*k*_(*v*) denote the number of mutations and their frequency in the *k* ^th^ epoch for the *v* ^th^ haplotype. Epoch *k* splits the haplotypes into *k* equivalence classes, which we call **k*-clades*. Let *m*_*k*,*i*_ and *w*_*k*,*i*_ denote the corresponding values on the *i* ^th^ lineage of the *k* ^th^ epoch. We compute the expected value by summing over all haplotypes and genealogies and dividing by *n*. The sum is

$$\begin{array}{ccc} \sum_{v=1}^{n}\ell \text{-HAF}\left(c\left(v\right)\right) & = & \sum_{k=2}^{n}\sum_{v=1}^{n}m_{k}\left(v\right){\left(w_{k}\left(v\right)\right)}^{\ell }\\ & = & \sum_{k=2}^{n}\sum_{i=1}^{k}\sum_{j=1}^{w_{k,i}}m_{k,i}{\left(w_{k,i}\right)}^{\ell }=\sum_{k=2}^{n}\sum_{i=1}^{k}m_{k,i}{\left(w_{k,i}\right)}^{\ell +1}. \end{array}$$ (6)

Let *M*_*k*,*i*_ and *W*_*k*,*i*_ be random variables denoting the number of mutations, and their frequency respectively, on the *i* ^th^ lineage of the *k* ^th^ epoch. As the genealogy of a neutrally evolving sample is independent of branch lengths [43], *M*_*k*,*i*_ and *W*_*k*,*i*_ are independent random variables. Thus, we can compute the expected ℓ-HAF score of a randomly chosen haplotype as

$$𝔼\left[\ell \text{-HAF}\right]=\frac{1}{n}\sum_{k=2}^{n}\sum_{i=1}^{k}𝔼\left[M_{k,i}W_{k,i}^{\ell +1}\right]=\frac{1}{n}\sum_{k=2}^{n}\sum_{i=1}^{k}𝔼\left[M_{k,i}\right]𝔼\left[W_{k,i}^{\ell +1}\right]\;.$$ (7)

To compute $𝔼[W_{k,i}^{\ell }]$, we start with a related quantity. For positive integer ℓ, denote the *rising factorial*

$$w^{\left(\ell \right)}=w\left(w+1\right)\left(w+2\right)\cdots \left(w+\ell -1\right)\;,$$ (8)

and set *w* ^(0)^ = 1. We show in S1 Text that

$$𝔼\left[{\left(W_{k,i}\right)}^{\left(\ell \right)}\right]=\ell !\cdot \frac{n^{\left(\ell \right)}}{k^{\left(\ell \right)}}\;.$$ (9)

We have *w* ^(1)^ = *w* and *w* ^(2)^ = *w*(*w*+1) = *w* ^2^+*w*, so *w* ^2^ = *w* ^(2)^ − *w* ^(1)^, which leads to:

$$𝔼\left[{\left(W_{k,i}\right)}^{2}\right]=𝔼\left[{\left(W_{k,i}\right)}^{\left(2\right)}-{\left(W_{k,i}\right)}^{\left(1\right)}\right]=\frac{2n(n+1)}{k(k+1)}-\frac{n}{k}=\frac{n(2n-k+1)}{k(k+1)}\;.$$ (10)

In S1 Text, we generalize this equation to compute 𝔼[(*W*_*k*,*i*_)^ℓ^]. In addition, we show that for a constant-sized population, the general form in Eq (7) produces the same result as Eq (3).

#### Exponential population growth

Eq (7) can potentially be used to obtain 𝔼[ℓ-HAF] under arbitrarily complex demographics. Consider a population of current size *N*_0_ that has been growing exponentially at a rate *r*. The population size at time *t* in the past is given by *N*(*t*) = *N*_0_ *e* ^−*rt*^. Exponential population growth is of particular interest, as it has been used to analyze the state of a population under a selective sweep *shortly after fixation*. This is a low point (or *trough*) of observed ℓ-HAF scores, as early hitchhiking sites have fixed by this time point, and the (relatively recent) sample MRCA is a carrier of the favored mutation. Immediately after fixation, the population—all of which are carriers of the favored allele—has been growing for the duration of the sweep at a rate that is approximately exponential (with growth rate related to the selection coefficient *s*). In addition, all extant and ancestral haplotypes since the sample MRCA are carriers and therefore equally favored, implying that the independence between *W*_*k*,*i*_ and *M*_*k*,*i*_ is kept. While the branch lengths and distribution of *M*_*k*,*i*_ values change under exponential growth, the distribution for *W*_*k*,*i*_ remains unchanged as described in Eq (10). This key insight allows us to use Eq (7) to estimate the expected HAF scores under exponential population growth.

In order to use 𝔼[*M*_*k*,*i*_] = *μ*𝔼[*T*_*k*_] under exponential growth, we implement two numerical methods to compute 𝔼[*T*_*k*_]: a ‘cumulative time’ method that uses an approximate distribution of *T*_*k*_ given in [44, p. 559], and a ‘conditional expectation’ method (see S1 Text for details). In the conditional expectation method, we compute the expected value of *T*_*k*_ conditioned on *T*_*k*+1_, …, *T*_*n*_, as follows (in the order *k* = *n*, *n* − 1, …, 2):

$$\begin{array}{ccc} t_{k} & = & 𝔼[T_{k}\;|\;T_{k+1}=t_{k+1},\ldots ,T_{n}=t_{n}]\\ & = & \frac{1}{r}\int_{0}^{1}\ln(1-\frac{\alpha }{k(k-1)}\;e^{-r\cdot \tau_{k}}\;\ln\left(u\right))\;du\\ & = & \frac{1}{r}\exp(\frac{k(k-1)}{\alpha }e^{r\cdot \tau_{k}})E_{1}(\frac{k(k-1)}{\alpha }e^{r\cdot \tau_{k}}), \end{array}$$ (11)

where *α* = 2 *N*_0_ *r* is the scaled growth rate, *τ*_*k*_ = *t*_*k*+1_ + ⋯ + *t*_*n*_ (with *τ*_*n*_ = 0), and *E*_1_(*x*) is the exponential integral $E_{1}\left(x\right)=\int_{1}^{\infty }\frac{\exp(-x\;t)}{t}\;dt$.

We then use 𝔼[(*W*_*k*,*i*_)^ℓ^] (evaluated in Eq. (S21) in S1 Text) to evaluate Eq (7), yielding 𝔼[ℓ-HAF] for exponential population growth as

$$𝔼\left[\ell \text{-HAF}\right]\approx \frac{\theta }{\alpha }\sum_{k=2}^{n}\left(r\cdot t_{k}\right)\sum_{q=0}^{\ell }{\left(-1\right)}^{\ell -q}S\left(\ell ,q\right)(\left(q+1\right)!\;\frac{{\left(n+1\right)}^{\left(q\right)}}{{\left(k+1\right)}^{\left(q\right)}}-q\cdot q!\cdot \frac{{\left(n+1\right)}^{\left(q-1\right)}}{{\left(k+1\right)}^{\left(q-1\right)}}),$$ (12)

where *S*(ℓ, *q*) denotes the Stirling number of the second kind [45, Ch. 6.1]. We describe these procedures fully in S1 Text.

#### Empirical validation of expected HAF score computation

We tested our theoretical calculations against empirical observations of HAF scores using simulations for neutral evolution with constant population size *N* = 20000 (see ‘Simulations’ in Methods). For example, for *θ* = 48 and *n* = 200, the expected 1-HAF is exactly 4776.0 (Eqs (3) and (7)), whereas the empirically observed mean 1-HAF score of 20000 simulated samples is 4786 ± 3956 (sample mean ± sample standard deviation). Interestingly, the estimates improve when simulating with recombination, with an observed mean of 4780 ± 1684 (S1 Fig).

We also modeled exponential growth in population size using the scaled growth rate *α*, using the conditional expectation method in Eq (12) (see S1 Text). As expected, the HAF score is much lower than for constant population size. For *α* = 80, *θ* = 48, *n* = 200, the theoretical mean 1-HAF score is 126.9, whereas the empirical mean of 20000 simulations is 128 ± 131.1 for *ρ* = 0, and 127.6 ± 127.4 for *ρ* = 25 (S2 Fig).

We compared the simulations with theoretical expected ℓ-HAF scores for multiple values of ℓ ∈ {1, 2, 3, 4} and different choices of the population-genetic parameters: scaled mutation rate *θ* ∈ {24, 48}, scaled growth rate *α* ∈ {0, 30, 60, 80}), and scaled recombination rate *ρ* ∈ {0, 25, 50} (see ‘Simulations’ in Methods). While theoretical expected values were computed assuming *ρ* = 0, S3 Fig shows the concordance between theoretical and empirical means for each choice of parameters. The concordance improves slightly for increasing values of *n*, ℓ. In each case, the values are robust to choice of *ρ*, and the variance even reduces slightly for higher *ρ* (S3D Fig).

As ℓ increases, the normalized HAF score (ℓ-HAF^1/ℓ^) distribution (S4 Fig) becomes more left-skewed and has generally smaller values (upper bound of range approaching *n* − 1), with reduced variance. Increasing ℓ increases the relative weight of ancient mutations. As an extreme example, the normalized ∞-HAF score is simply the weight of the highest frequency mutation on the haplotype, and not very informative. However, very recent mutations, including those that appear post-selection among the carriers of the favored allele add ‘noise’ to the HAF-score, and an appropriate choice of ℓ > 1 may perform better for some applications. We will explore this in future work.

### HAF score dynamics in selective sweeps

**Fig 2:**
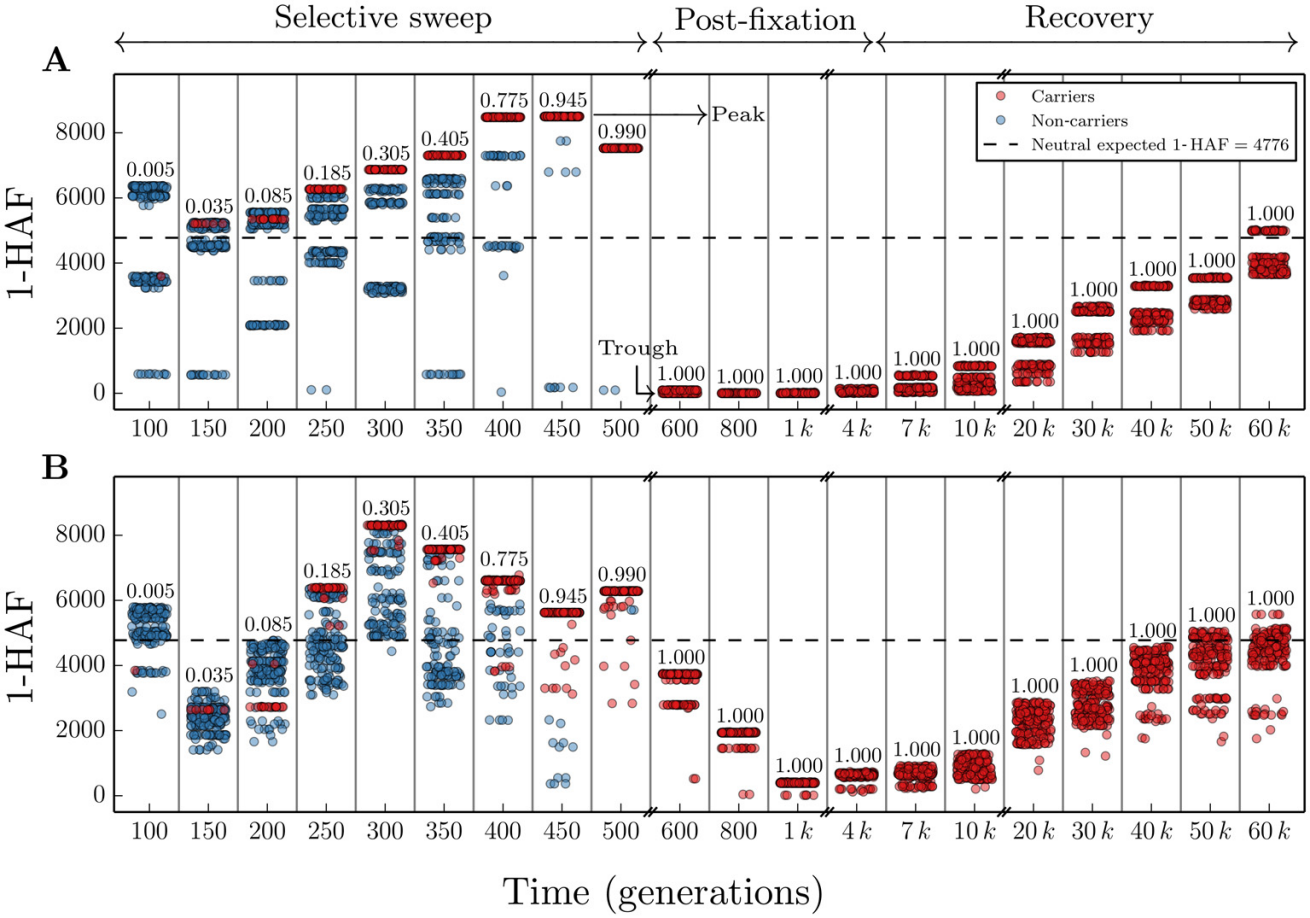
Schematic of HAF score dynamics. We consider HAF scores in 50 kb segments, examining *n* = 200 haplotypes sampled from a constant-sized (*N* = 20000 haploids) population, evolving with population-scaled mutation rate *θ* = 48 and selection coefficient *s* = 0.05. We do forward simulations, with time *t* = 0 at the onset of selection and *t* increasing towards the present time. Snapshots of generations are shown at specific times indicated at tick marks on the *x*-axis. Note that these times are increasing but neither consecutive nor regularly spaced. Each selected generation is depicted as a tall thin rectangle. The number in each rectangle is the frequency of the favored allele (carriers). A few rectangles are shown for each phase of a simulated population undergoing a selective sweep. Each point within a rectangle represents the 1-HAF score of a randomly chosen haplotype. Red points represent carriers of the favored allele and blue points represent non-carriers. Points are scattered randomly on the *x*-axis within each rectangle, but all points within the same rectangle represent the same generation at the time indicated by the tick mark on the *x*-axis, regardless of their horizontal position within the rectangle. Darker shades of red or blue indicate a higher density of points at that level. The dotted line represents the expected 1-HAF score in the neutral population. (A) Simulation of a non-recombining segment. (B) Simulation with population-scaled recombination rate *ρ* = 25 (see Methods).

We now consider the dynamics of HAF scores in a population undergoing a selective sweep. To do this, we use data simulated under several scenarios. Fig 2 illustrates the HAF score dynamics in a single simulated population undergoing a hard sweep, with selection coefficient *s* = 0.05. See ‘Simulations’ in Methods for a detailed description of the simulation parameters. Initially (leftmost, time 0) the HAF scores of carriers and non-carriers of the favored allele are similar. As the sweep progresses (times 100–450), carrier HAF scores increase to a peak value (HAF-peak). Soon after fixation (time ∼450), we observe a sharp decline in HAF scores (HAF-trough), followed by slow and steady recovery due to new mutation and drift (times 500–50000). We observe similar behavior for the HAF score dynamics in an exponentially growing population, and soft sweep scenarios (S5 and S6 Figs). Though soft sweeps can arise under different circumstances, we restrict our attention to soft sweeps arising from standing variation. While the behavior is similar, we note that during a soft sweep, the HAF scores do not have as sharp a decline as in the hard sweep scenarios.

Below, we provide a theoretical description of these dynamics, as well as empirical validation using simulations. This allows us to predict HAF scores in (a) the post-fixation trough; (b) the pre-fixation peak; and (c) the rate of growth of HAF scores from pre-sweep to peak value.

#### Empirical validation of the post-fixation HAF-trough

We showed using simulations that the HAF score computations for an exponentially growing population (Eq (12)) also approximate a population evolving under a selective sweep *shortly after fixation*. This enables prediction of the HAF-trough value.

**Fig 3:**
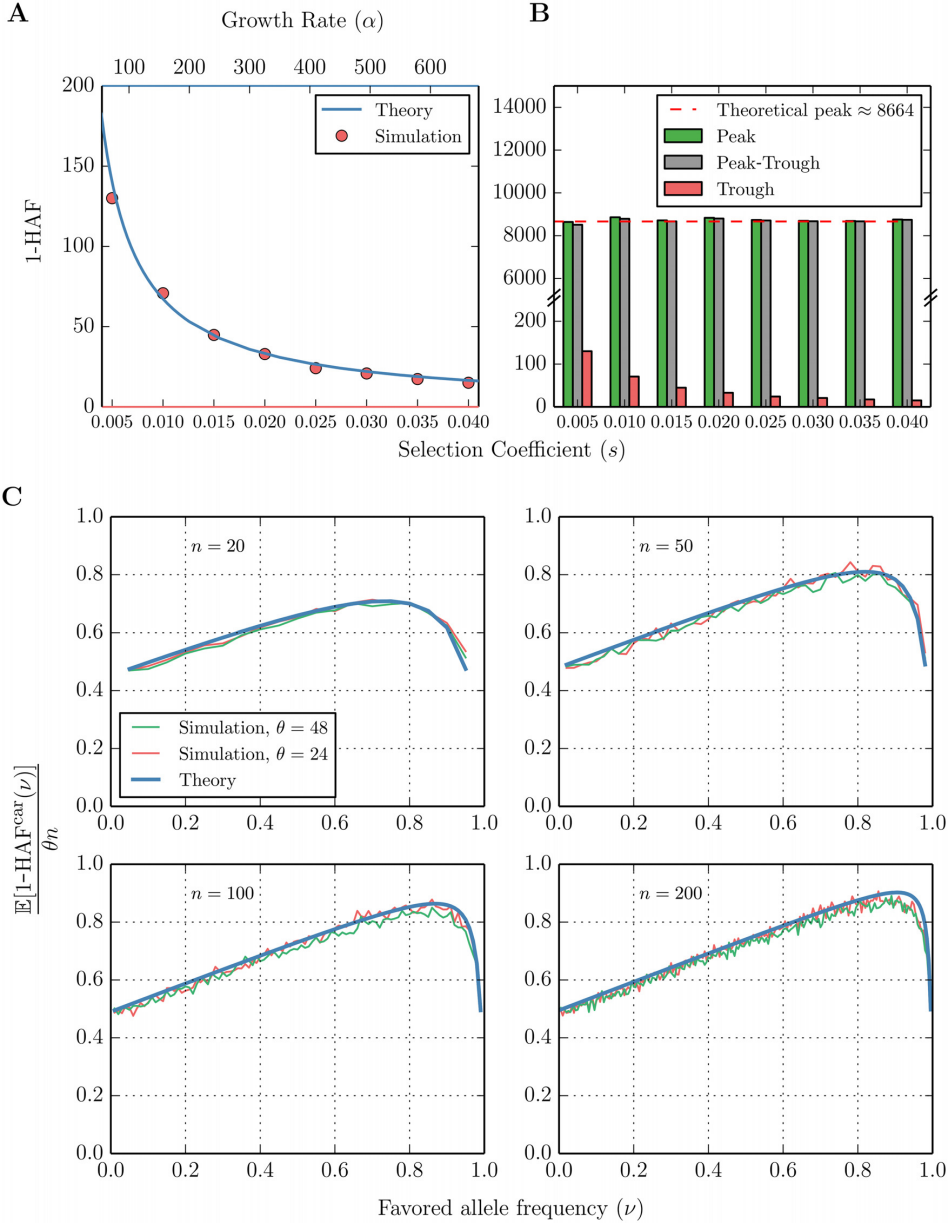
HAF scores in a selective sweep ‘peak’ and ‘trough’. (A) Observed values (red) of the mean ‘trough’ 1-HAF scores in simulated selective sweeps with coefficients *s* ∈ [0.005, 0.040]. Theoretical values (blue) of expected 1-HAF scores under exponential population growth with population-scaled rates *α* ∈ [100, 600] given by Eq (12). Simulated 1-HAF scores (red) represent the mean of 2000 simulated population samples for each value of *s*, with *θ* = 48, *n* = 200. (B) Observed mean 1-HAF peak, trough, and difference (peak minus trough) for selective sweeps with coefficients *s* ∈ [0.005, 0.040]. The dashed line represents the approximate value of the peak 1-HAF score given by Eq (15). (C) Dynamics of the expected value of 1-HAF^car^ (1-HAF score of haplotypes carrying the favored allele) plotted as a function of the fraction of carriers (*ν*) in the sample during a selective sweep. For each (*θ*, *n*, *ν*) with *θ* ∈ {24, 48}, *n* ∈ {20, 50, 100, 200}, $\nu \in \{\frac{1}{n},\frac{2}{n},\ldots ,\frac{n-1}{n}\}$, *s* = 0.01, and *N* = 20000, we plotted the mean value of (1-HAF^car^)/(*θn*) over 1000 trials, and compared against the theoretical values (Eq (13)).

The HAF-trough of a sweep is the value of 1-HAF at fixation. We took the mean of the HAF-trough values over 200 populations simulated under selective sweeps with coefficients *s* ∈ [0.005, 0.040] (see ‘Simulations’ in Methods), and compared it to 1-HAF values in simulated neutral populations growing exponentially at rates *α* ∈ [100, 600]. Fig 3A shows a close similarity between the 1-HAF values under exponential growth (blue) and the selective sweep trough (red).

#### The pre-fixation 1-HAF-peak

As the selective sweep progresses, the value of the HAF score of haplotypes carrying the favored allele increases, eventually reaching a peak value. Consider *n* haplotypes sampled from a fixed population of *N* haploid individuals under a selective sweep. Let *μ* denote the mutation rate per base per generation in the genomic region of interest, and assume that there is no recombination. The scaled mutation rate is given by *θ* = 2*Nμ*.

We let *ν* denote the fraction of carrier haplotypes in the sample. When *ν* ≤ 1/*n* (i.e., 0 or 1 carriers), there is no selection going back in time, and the time to MRCA can be computed using the neutral Wright-Fisher model [46]. The expected 1-HAF scores for carriers and non-carriers are identical (Eq (3)). At the time when *ν* first equals 1, there are no non-carriers, and the HAF-scores are given by the exponential growth model. In S1 Text, we model the 1-HAF scores for all intermediate values of *ν*.

Let 1-HAF^car^ (respectively, 1-HAF^non^) denote the 1-HAF score of a random haplotype carrying the favored allele (respectively, a non-carrier) when a fraction *ν* of the *n* sampled haplotypes carry the favored allele. In S1 Text, we show that under strong selection (*Ns* ≫ 1) and no recombination (*ρ* = 0),

$$𝔼\left[1{\text{-HAF}}^{\;\text{car}}\right]\approx \theta n(\frac{\nu +1}{2}-\frac{1}{(1-\nu )n+1}),$$ (13)

$$𝔼\left[1{\text{-HAF}}^{\;\text{non}}\right]\approx \theta n(\frac{1}{2}+\frac{1}{2n}-\frac{1}{(1-\nu )n+1}).$$ (14)

For any sample of size *n*, the carrier haplotypes reach a peak value of 1-HAF^car^ as *ν* varies along its trajectory. We do not compute the expected value of this peak (𝔼[max_*ν*_(1-HAF^car^(*ν*, *n*))]) directly. Instead, we compute the peak value of 𝔼[1-HAF^car^(*ν*, *n*)] (maximizing over all *ν* ∈ [0, 1]) as

$$\max_{\nu }𝔼\left[1{\text{-HAF}}^{\;\text{car}}\left(\nu ,n\right)\right]=\theta n{\left(1-\frac{1}{\sqrt{2n}}\right)}^{2}\approx \theta n\;.$$ (15)

Note that under strong selection, this peak does not depend on *s* (see Fig 3B).

The trough for each trajectory is computed as the 1-HAF score at fixation (when *ν* = 1 is first reached).

#### Empirical validation

Simulated data under selective sweeps with coefficients *s* ∈ [0.005, 0.040] show that for strong selection (*Ns* ≫ 1) (i) the pre-fixation HAF peak scores appear to be independent of the selection coefficient (Fig 3B), and (ii) as predicted by Eq (15), the mean value of the HAF peak score is approximately *θn*. We also simulated (1-HAF^car^)/(*nθ*) as a function of *ν* (Fig 3C and S15 Fig). The results show a tight correspondence between theory and empirical observations.

### HAF score application: Characterizing carriers and non-carriers

Our understanding of the dynamics of HAF scores of a haplotype during a selective sweep has many potential applications. For example, we could compare the dynamics of hard and soft sweeps to distinguish between the two events. Second, HAF scores of haplotypes in a region under selection might help predict the future MRCA of a population. Finally, by conditioning on known or deduced selective sweeps in a population sample, we can predict the state (carrier/non-carrier) of the favored allele in its haplotypes. Below we explore the last application, leaving the first two to future work.

**Fig 4:**
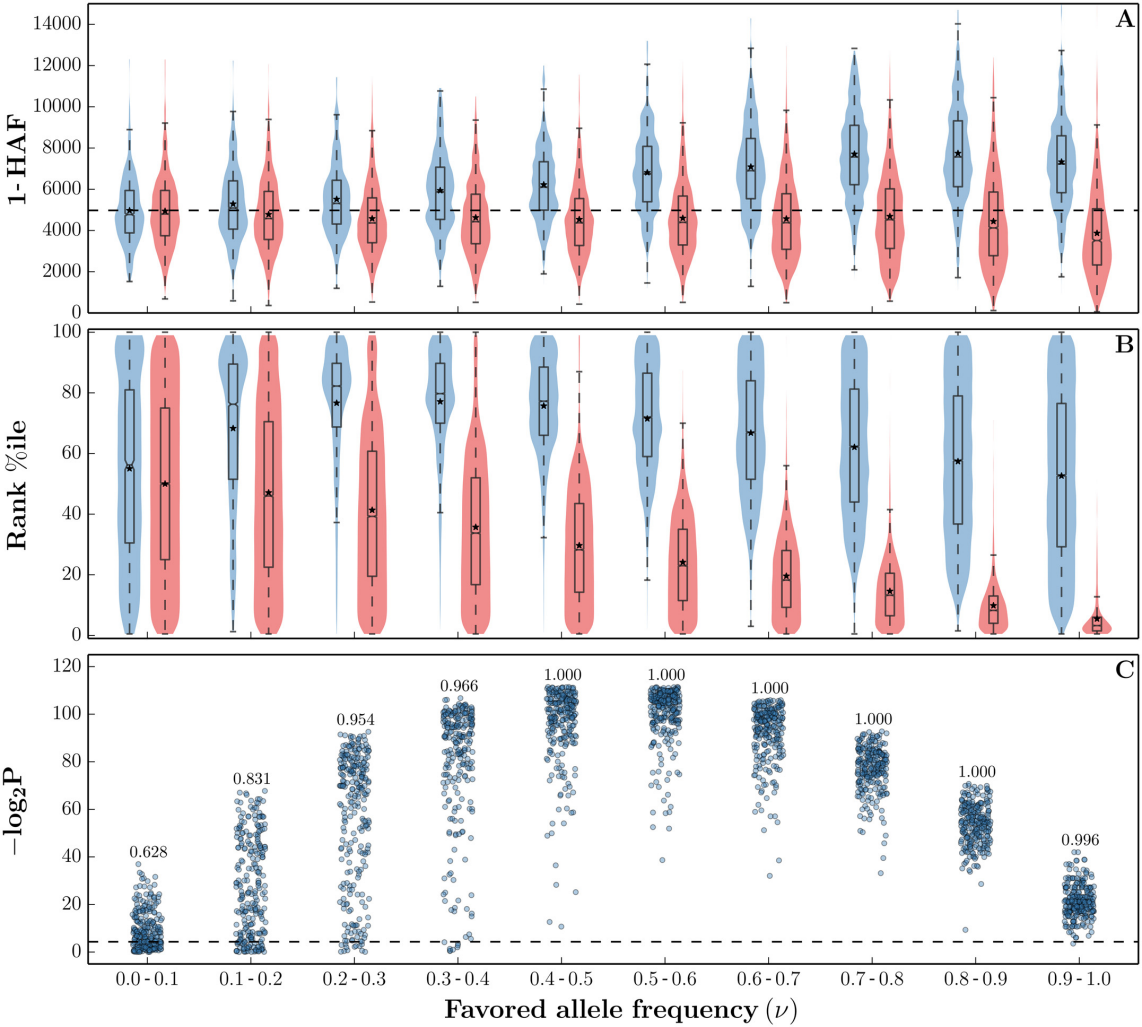
HAF score dynamics in ongoing selective sweeps. HAF scores were computed from 250 simulated population samples (*n* = 200) undergoing a hard sweep (*θ* = 48, *ρ* = 25, *s* = 0.01), using the simulation software *msms* [47]. (A) Each violin shows the Gaussian kernel density estimation (KDE) of 1-HAF scores in carriers (blue) and non-carriers (red) of the favored allele, as the sweep progresses in frequency. A standard box plot is overlaid on each violin to mark the 25^th^, 50^th^, and 75^th^ percentiles, with means indicated by asterisks. The horizontal dashed line represents the expected 1-HAF scores under neutrality (Eq (4)). (B) Corresponding violins showing the *in-sample* percentile rank of 1-HAF scores. (C) −log_2_(*P*) values for Wilcoxon rank sum tests rejecting the null hypothesis of identically distributed 1-HAF scores among carriers and non-carriers *within* each population sample. The number above each bin indicates the fraction of significant tests (where *P* < 0.05, shown by the dashed line).

In Fig 4A, we show the distributions of haplotype 1-HAF scores aggregated from 500 simulated populations undergoing a hard selective sweep (see ‘Simulations’ in Methods for detailed parameter choices). Scores were computed for random samples of *n* = 200 haplotypes taken at regular time intervals. They are stratified by the frequency of the favored allele at the time of sampling. Further, scores are stratified into carrier and non-carrier classes (of the favored allele). As with a single population, HAF scores of carriers and non-carriers diverge as the sweep progresses in frequency. We note, however, that even close to fixation (frequencies 80–100%) the distributions of HAF scores between carriers and non-carriers maintain considerable overlap. The high variance in HAF scores makes them only weakly informative of sweep carrier status when comparing across population samples (or genomic regions within a single population). Within a single population sample, however, the HAF scores are highly informative of the carrier status. This is illustrated in Fig 4B, showing the distributions of HAF score percentile rank within their respective samples. We observe that the rank distributions have minimal overlap for carriers and non-carriers of the favored allele. Any remaining overlap in the percentile rank distributions in the final stages of a sweep (favored allele frequency ≥ 70%) stems mostly from recombination, which allows the favored allele to recombine onto haplotypes outside the selected clade (creating low HAF score carriers) and vice-versa (creating high HAF score non-carriers). The overall strong separation between carriers and non-carriers is further illustrated by the highly significant *P*-values of Wilcoxon rank sum tests rejecting the null hypothesis of identically distributed HAF scores among carriers and non-carriers *within* each population sample (Fig 4C).

Fig 4 does not show how HAF scores are distributed following fixation of the sweep. Starting at fixation, we see a strong decline in HAF scores owing to the loss of many high frequency derived alleles from the pool of segregating sites. However, crossover events may unlink hitchhiking alleles from the favored allele, and they may remain segregating in the population even after fixation of the favored allele. Therefore, the decline in HAF scores may be abrupt or gradual, depending on the linkage between the favored and hitchhiking alleles. Finally, after reaching a trough, HAF scores gradually recover to their neutral levels over time. The post-fixation dynamics of HAF scores are shown in S7 Fig.

#### PreCIOSS: Predicting Carriers of Ongoing Selective Sweeps

Our simulations suggest that, in a region undergoing a selective sweep, we could use HAF scores to predict whether a haplotype is carrying the favored allele. We implemented a simple algorithm (PreCIOSS) to carry out this prediction by clustering HAF scores in a sample. PreCIOSS takes as input a set of binary haplotypes sampled from a population undergoing a selective sweep. For each haplotype, the ℓ-HAF score is computed (ℓ = 1 by default). We then fit a Gaussian Mixture Model (GMM) with exactly two Gaussians to the haplotype HAF scores. The fit is performed using Expectation Maximization (EM). Finally, we apply the fitted model to assign a label to each haplotype according to the Guassian component to which it is assigned. Haplotypes whose HAF score is higher are denoted as ‘carriers’.

We apply PreCIOSS to data from simulated populations undergoing hard and soft sweeps (see ‘Simulations’ in Methods). The haplotypes predicted as carriers might in fact be carriers (True Positives, TP) or non-carriers (False Positives, FP). Similarly, the haplotypes predicted as non-carriers could be True Negatives (TN) or False Negatives (FN). We measure the *balanced accuracy*

$$\frac{1}{2}(\frac{TP}{TP+FN}+\frac{TN}{FP+TN}),$$ (16)

which is more appropriate to use than *Rand accuracy* (*TP*+*TN*)/(total predictions) when the positive and negative classes appear at different proportions in the sample [48].

**Fig 5:**
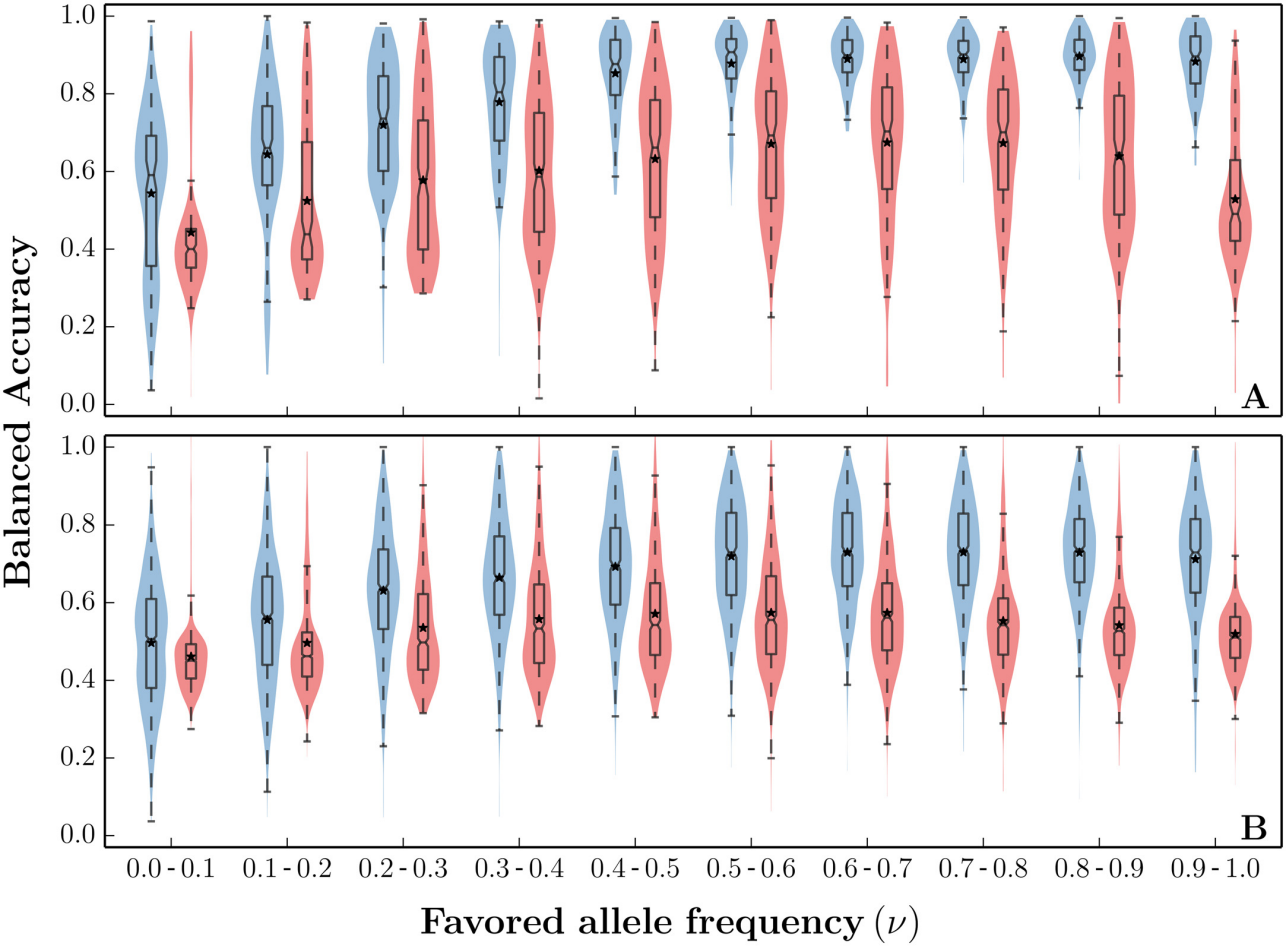
Predicting carriers of hard and soft sweeps. Balanced accuracy (Eq (16)) of PreCIOSS in populations undergoing hard and soft sweeps. For each frequency bin, (A) 200 samples were simulated (*n* = 200, *θ* = 48, *ρ* = 25) undergoing a hard sweep (*s* = 0.01, *ν*_0_ = 1/20000), and (B) 200 samples were simulated undergoing a soft sweep (*s* = 0.01, *ν*_0_ = 0.02). We split each sweep into intervals as *ν* progresses ([0.0, 0.1] through [0.9, 1.0]). For each *ν* interval, we show the distribution of balanced accuracy using standard violin plots (blue). For comparison, we also plotted the balanced accuracy of iHS adapted to predicting carrier haplotypes (red).

While there are no tools currently available that directly predict the carrier state of a haplotype, some approaches are relevant. For example, Grossman et al. [49] developed a ‘composite of multiple signals’ (CMS) statistic to reduce the number of candidates for the favored mutation, but CMS cannot directly be used to identify carriers of the favored mutation. Similarly, the iHS statistic uses the dominant haplotype frequency decay in a window centered around each locus, as a test for recent positive selection [30]. As a comparison, we used iHS to distinguish carriers from non-carriers based on segregating alleles at the locus with peak iHS score. The balanced accuracy of PreCIOSS on hard sweeps is shown in Fig 5A for a specific choice of parameters (200 samples with *n* = 200, *θ* = 48, *ρ* = 25, *s* = 0.01). Once the sweep reaches frequencies above 30%, the balanced accuracy increases (median ∼70%) and remains high (median ∼90%) for the remainder of the sweep. At the beginning of the sweep, the balanced accuracy, despite being asymptotically unbiased, suffers from high variance due to the severe class imbalance (few carriers in the beginning, few non-carriers at the end). The accuracy is reduced for soft sweeps (Fig 5B, run with similar parameters), as increasing the carrier haplotype frequency leads to higher variance in 1-HAF scores.

We tested PreCIOSS under a wide range of population-genetic parameters (S1 Table), and observed consistently high balanced accuracy in carrier-state prediction as the sweeps progressed (S8 Fig). Specifically, PreCIOSS is quite robust to changes in sample size (S8A–S8D Fig). A higher recombination rate has only a limited impact (S8A and S8H Fig), while setting *ρ* = 0 shows reduced performance at an early stage of the sweep (S8A and S8G Fig). This is consistent with selection acting more efficiently in the presence of recombination.

We tested the effect of the position of the carrier mutation (unknown to PreCIOSS) on the performance of PreCIOSS. We considered different 50 kb windows, with the carrier mutation located at one end (0 kb), and moving towards the middle (25 kb). For each location of the carrier, we simulated 200 samples with *n* = 200, *θ* = 48, *ρ* = 25, *s* = 0.01 (S9 Fig), but did not observe a marked change in accuracy. However, when the favored allele is in the middle of the window, the median balanced accuracy is generally higher and has lower variance (S10 Fig).

**Fig 6:**
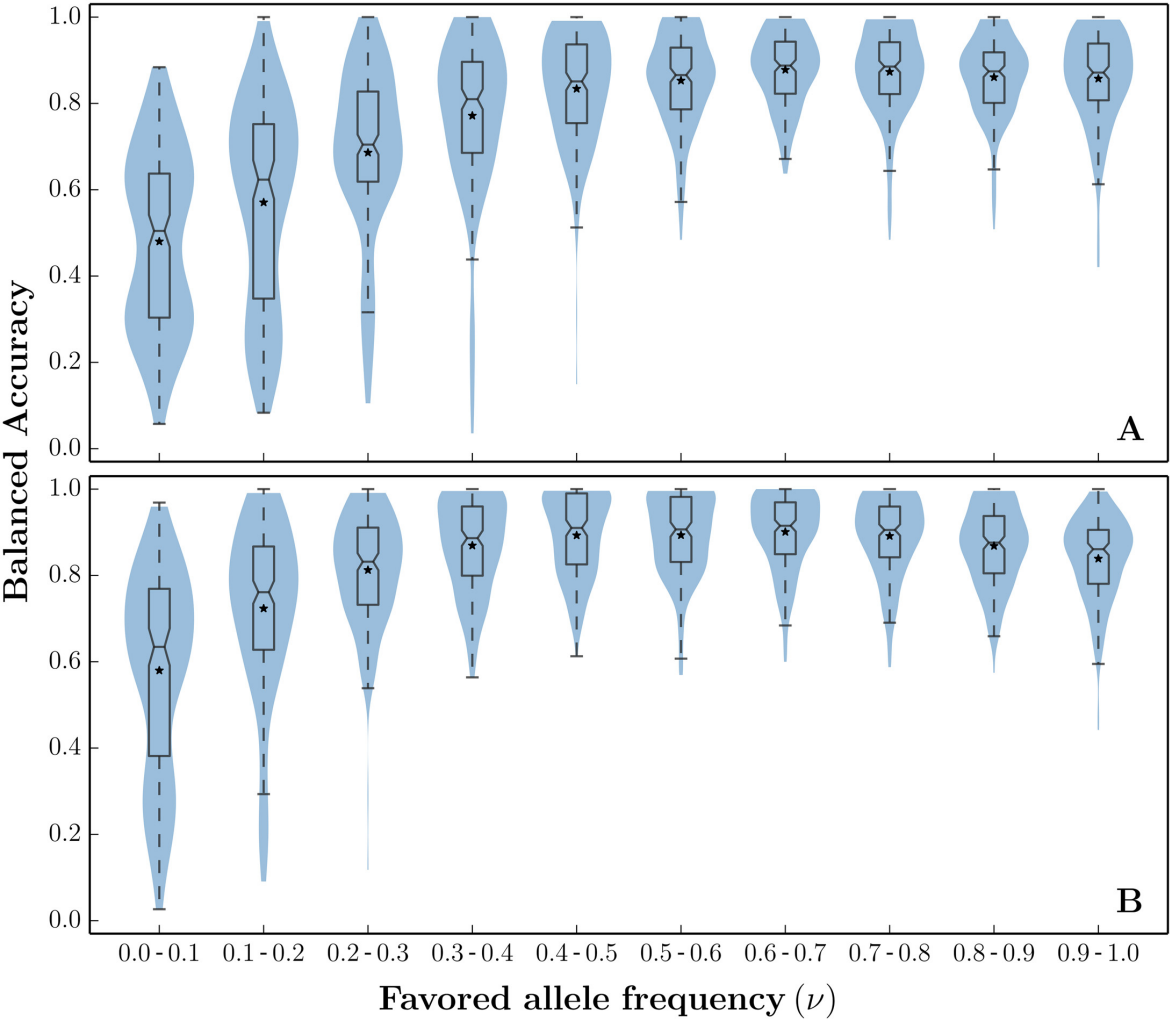
Balanced accuracy of PreCIOSS on a model of European demography. Populations were simulated for a popular model of human demography (S11 Fig and Gravel et al. (2011) [50]). The onset times of selection were separated into (A) pre-bottleneck (51 kya–23 kya) and (B) post-bottleneck (23 kya–current) epochs, with 10000 start times in each bin. All samples were simulated with *n* = 200, *θ* = 48, *ρ* = 25. Samples were simulated with selection coefficient *s* = 0.005 in the pre-bottleneck epoch and *s* = 0.02 in the post-bottleneck epoch.

Finally, we tested PreCIOSS on a popular model of European demography [50]. The model (S11 Fig) suggests an Out-of-Africa migration 51 kya (51 thousand years ago), followed by a European and East Asian split 23 kya. It also suggests bottlenecks that reduced the effective population sizes of the European (*N*_Eu0_ = 1032), and East-Asian (*N*_As0_ = 550) populations, and exponential growth in the populations following the bottleneck events. We simulated populations based on this model, as well as selection events (hard sweep) at different times after the Out-of-Africa migration, and partitioned all samples into two categories depending on whether the selection event happened before or after the bottleneck. These scenarios are challenging for most tests of adaptation (see, e.g., [23]). However, there are still significant differences in the 1-HAF scores of carriers and non-carriers. The balanced accuracy of PreCIOSS is shown in Fig 6A for ancient selection and Fig 6B for recent (after bottleneck) selection. The performance is quite robust, although somewhat worse in the early stages of the sweep. Once the favored allele frequency reaches 60%, the median accuracy is at 0.9. The accuracy is improved for recent adaptation, compared to ancient adaptation. Even for very recent sweeps, where the carrier frequency is 30–40%, the median balanced accuracy is close to 0.8. We used a lower selection coefficient for ancient selection compared to recent selection to ensure that we have sufficient cases of incomplete sweeps. Not surprisingly, the performance of PreCIOSS is worse for ancient selection compared to recent selection.

**Fig 7:**
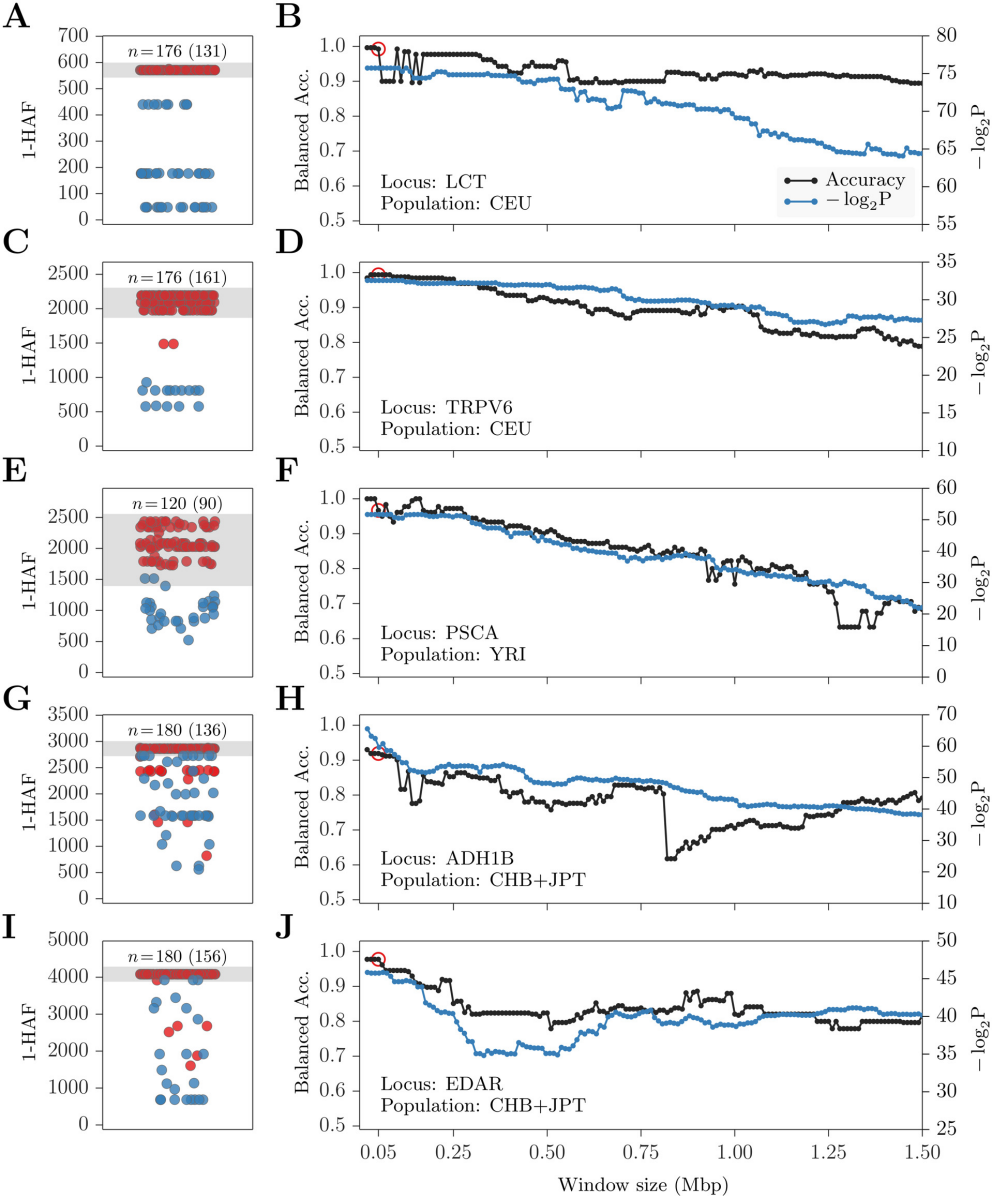
Predicting carriers of well-known selective sweeps. (Left): Haplotype 1-HAF scores in a 50 kb window centered at known favored sites indicated by the gene name, and the SNP identifier. (A) LCT/rs4988235, (C) TRPV/rs4987682, (E) PSCA/rs2294008, (G) ADH1B/rs1229984, and (I) EDAR/rs3827760. Points represent haplotype 1-HAF scores, red indicating a carrier of the favored allele and blue indicating a non-carrier. At the top of each panel, the number of haplotypes, *n*, is shown, with the number of carriers in parenthesis. Areas shaded in gray indicate haplotypes designated as ‘carrier’ by PreCIOSS. (Right) classification Balanced Accuracy (black) and −log_2_(*P*) values (blue) as function of window size around the favored allele in (B) LCT, (D) TRPV6, (F) PSCA, (H) ADH1B, and (J) EDAR. *P*-values are for Wilcoxon rank sum tests rejecting the null hypothesis of identically distributed 1-HAF scores among carriers and non-carriers. Red circles indicate the 50 kb windows shown on the left.

Our results suggest that for cases of recent adaptation (e.g., lactase adaptation, shown in Fig 7A, which happened between 2 kya and 20 kya and rapidly spread to high frequencies in the European population), PreCIOSS would show good performance in separating the carriers and non-carriers.

### Applying PreCIOSS to human selective sweeps

To evaluate the effectiveness of PreCIOSS in distinguishing carriers of a selective sweep from non-carriers, we applied it to several genomic regions (e.g., [39]) where (i) there is strong evidence of a selective sweep, and (ii) the favored allele has been characterized. In applying PreCIOSS to the datasets, we assumed that the region was known, but did not supply the favored allele to PreCIOSS. In each case, we tested if PreCIOSS could separate the haplotypes that carried the favored allele. We use phased haplotypes from the HapMap project [51], setting the ancestral allele to that observed in orthologous Chimpanzee sequence [52].

#### LCT

We consider the well-known sweep in the lactase (LCT) gene region in Northern Europeans. The best characterized variant is C/T-13910 (rs4988235), for which the T allele was found to be 100% associated with lactase persistence in the Finnish population [53]. T-13910 was further shown to be causal by in-vitro analysis, where it was found to increase enhancer activity [54, 55]. We considered haplotypes from the CEU population, applying PreCIOSS to a 50 kb window centered at C/T-13910 (Fig 7A). This yielded 100% accuracy in classifying carriers from non-carriers. Increasing the window size above 50 kb, the balanced classification accuracy reduced to ∼90% (Fig 7B). LCT shows the highest and most prolonged (with increasing distance from the causal site) statistical significance in separating carrier and non-carrier HAF scores, remaining highly significant for haplotypes of 1.5 Mb (Fig 7B, blue line). Despite this highly significant separation, the classification accuracy is initially unstable, alternating between ∼100% (perfect classification) and 90%. This is due to the pattern of HAF scores observed in the LCT region, where carriers form a tight cluster with the highest scores, but several non-carriers cluster closer to carriers than to the majority of other non-carriers (Fig 7A). These haplotypes are therefore sometimes included (90% accuracy) and sometimes excluded (100% accuracy) from the reported ‘carriers’ class.

#### TRPV6

Transient Receptor Potential Cation Channel, Subfamily V, Member 6 (TRPV6) is a membrane calcium channel thought to mediate the rate-limiting step of dietary calcium absorption. It is reportedly under strong positive selection in several non-African populations [56, 57]. Following Peter et al. (2012) [39], we focus on the CEU population and set rs4987682 as the favored allele. This site, of the three non-synonymous SNPs with highest allele frequency differentiation among human populations, is the only one located in the the N-terminal region of TRPV6, thought to be the target of selection [58]. Applying PreCIOSS to a 50 kb window centered at this site, we obtain ∼99% balanced classification accuracy (Fig 7C), which gradually decays to ∼80% when considering a 1.5 Mb window (Fig 7D). As with LCT, the separation in HAF scores between carriers and non-carriers of the allele is highly statistically significant (*P* < 10^−7^; see Fig 7D). Unlike LCT, accuracy decays stably with distance from the favored site. This appears to be due to the less complex clustering pattern of non-carrier HAF scores in the region (Fig 7C).

#### PSCA

Prostate Stem Cell Antigen (PSCA) has been proposed to be under selection in a global analysis of allele frequency differentiation [59]. The putative causal site is a non-synonymous SNP (rs2294008) known to be involved in several cancer types [60, 61]. Interestingly, the derived allele is observed in all human populations, but at vastly different frequencies. It is most common in West Africa and East Asia, where it segregates at ∼75% frequency. We consider haplotypes from the YRI population and apply PreCIOSS to a 50 kb window centered at rs2294008, yielding balanced accuracy of 97% (Fig 7E). Unlike LCT and TRPV6, accuracy decays more noticeably with distance, reaching 63% at 1.25 Mb (Fig 7F). This sharper decay in accuracy is even more pronounced when considering the sweep in the CHB population (S12 Fig). Such decay is consistent with a (*soft*) sweep from the standing variation, which would allow more time for recombination to break the linkage between the favored allele and hitchhiking variation. Indeed, the sweep in PSCA was proposed to be from the standing variation by Bhatia et al. (2011) [59], and further substantiated as such by Peter et al. (2012) [39].

#### ADH1B

ADH1B encodes one of four subunits of Alcohol dehydrogenase (ADH1), which plays a key role in alcohol degradation. ADH1 genes (including ADH1B) have been studied extensively on both a functional and a population-genetic level, as they are thought to be one of the major drivers of alcoholism risk [62]. These genes have also been suggested to cause the “alcohol flush” phenotype common in Asian populations [63]. A specific non-synonymous mutation in ADH1B (Arg47His, rs1229984) has been proposed to be the target of selection. This is because (i) the derived allele has been shown to cause increased enzymatic activity [64, 65], and (ii) the estimated age of the allele coincides with rice domestication [63, 66] and the availability of fermented beverages [67]. Computing HAF scores for phased haplotypes from East Asian populations (CHB+JPT) and applying PreCIOSS, we obtained balanced classification accuracy of 92% using a 50 kb window centered at rs1229984 (Fig 7G). Both accuracy and statistical significance (of class separation) gradually decay with increasing window size (Fig 7H). As before, statistical significance decays more stably than classification accuracy.

#### EDAR

EDAR encodes a cell-surface receptor and has been associated with development of distinct hair and teeth morphologies [68, 69]. Specifically, a non-synonymous SNP (rs3827760, V370A) has been associated with these phenotypes [70]. The SNP is located within a DEATH-domain, which is highly conserved in mammals [71], and has been experimentally confirmed (in vitro) to increase EDAR activity [70]. It is found at very high frequencies in East Asian and American populations, while being completely absent from Europeans and Africans [70]. The EDAR gene has been found to be under selection in multiple studies [30, 56, 72], showing one of the strongest signatures of selection genome wide among the 1000 Genomes populations [39]. Applying PreCIOSS to phased CHB+JPT haplotypes in a 50 kb region centered at rs3827760, we obtained 98% balanced accuracy in predicting carriers vs. non-carriers of the allele.

In each case, PreCIOSS was applied to a 50 kb window centered at the favored allele, and separated the carriers and non-carriers with high accuracy of 97–100% (Fig 7A–7F). The accuracy decayed with increasing window size, but in many cases stayed high even for windows of 1.5 Mbp.

## Discussion

This paper introduces a new perspective on the genetic signatures of selective sweeps. From identifying and characterizing sweeps in a population sample—the topic of typical studies of selective sweeps—we progress to considering the role of individual haplotypes within an ongoing sweep. Using both simulated and real data, we show that the HAF score is well-correlated with the *relative* fitness of individual haplotypes, and that our algorithm (PreCIOSS) is highly effective at predicting carriers of selective sweeps.

The HAF framework has many natural extensions and potential applications. On the theoretical side, we have obtained the expected HAF score in both constant-sized and exponentially growing populations evolving neutrally (Eqs (3) and (12)). However, we do not yet know the variance. This quantity would provide a better understanding of the respective distributions, and a means to to statistically test for deviations from neutrality. Moreover, although we have observed in simulation and in practice that our theoretical argument is robust to recombination (genealogies violating a tree structure), a theoretical argument supporting these observations would be valuable.

In terms of application, several additional directions are worth investigating. The HAF framework is potentially useful in distinguishing hard from soft sweeps. Intuitively, hard sweep genealogies will likely have a single hitchhiking branch dominating the HAF scores, and leading to near-uniform scores in favored haplotypes. However, soft sweep genealogies may have several hitchhiking branches, potentially leading to distinct HAF score peaks. Even if the different favored clades happen to have similar scores, the haplotypes within them will not form a highly-related group as expected in hard sweeps.

Our results on known selective sweeps in humans illustrates this idea already (Fig 7). A recent study by Peter et al. (2012) [39] assigned posterior probabilities to hard vs. soft sweeps occurring in the same genes. Peter et al. assigned the highest likelihood of a hard sweep to LCT (0.99), followed by EDAR (0.89), ADH1B (0.78), TRPV (0.45), and finally PSCA (0.24). This is in striking concordance with the spread in HAF scores in Fig 7. The clusters capturing the carriers in LCT and EDAR have tightly distributed HAF scores (Fig 7A and 7I). The cluster for ADH1B (Fig 7G) has more variance by comparison, and the variance increases for TRPV6 (Fig 7C) and PSCA (Fig 7E), with PSCA showing the highest variance of HAF scores in carriers.

Finally, perhaps the highest potential impact of the HAF score could be in predicting the ‘MRCA of the future’. We know that future haplotypes are more likely similar to favored individuals than to unfavored ones, and that HAF scores correlate well with relative fitness in ongoing selective sweeps. Therefore, high HAF haplotypes are more likely to be similar to future generations. This relationship is particularly valuable when action may be taken based on such predictions. For instance, rapid influenza viral evolution is known to change the strain composition from year to year. The mutations are a mix of favored and deleterious mutations. The fitness and frequency of the current year’s strain have been used to predict the next year’s dominant strain [73]. The HAF score may allow for a careful look at the dynamics of the current strain and possibly offer better insight into the problem. As a second example, tumor cells show great heterogeneity and much variation occurs at the single cell level. This intra-tumor variation allows sub-population of cells to resist therapy and proliferate [74]. Once again, HAF scores of haplotypes in cells undergoing treatment can potentially distinguish between carriers and non-carriers of drug resistance mutations, and thereby improve our insight into mechanisms of drug resistance.

## Methods

### Simulations

We simulated data for various evolutionary scenarios. Neutral samples and sweep samples were generated using the simulator *msms* [47]. All simulations generated samples of *n* ∈ [20, 400] haplotypes from a larger effective population of *N* = 20000 haplotypes, each of length 50 kb. A mutation rate of approximately *μ* = 2.4⋅10^−8^ mutations per bp per generation was used [75, 76]. For our simulations, we choose a population-scaled mutation rate *θ* ∈ {24, 48}. For human recombination events, a population scaled rate of *ρ* = 1.32*θ* has been proposed (e.g. [77]). We use simulations either with no recombination, or with *ρ* ∈ {25, 50} in a 50 kb region to approximate human rates.

For exponential growth, we used *N* = 20000 as the size of the final (current) population. Let *r* denote the growth rate per generation, so that at *t* generations prior to the current generation, the population size was *N*(*t*) = *Ne* ^−*rt*^. Define the scaled growth rate *α* = 2*Nr*. We set *α* to a range of values in [200, 1600].

For selective sweeps, we used forward simulations assuming a diploid population with recombination and mutation parameters as described above. While diploid populations were simulated to incorporate recombination, we used phased haplotypes for our analysis. We assumed a single favored allele under selection coefficient *s* ∈ [0.005, 0.050] and heterozygosity 0.5 (haploid carriers get half the fitness advantage of diploid carriers). When *s* is ‘low’ (0.001 ≤ *s* ≤ 0.01), the available tests do not detect a selective sweep with reasonable power [21, 23]. Selection with *s* ≥ 0.08 is considered ‘high’ (e.g., see [21]). For high values of *s*, the carrier haplotypes are identical or very similar in simulations, making the problem of detecting carriers easy. Therefore, we chose intermediate values (*s* ∈ [0.005, 0.050]) in our simulations.

Soft sweeps can arise either due to standing variation or due to multiple favored alleles. Here, we focus on the former, where the favored allele is present in at least one carrier in the population (*ν*_0_ ∈ [1/*N*, 1]), and drifting at the onset of selection. In our simulations, we set *ν*_0_ at the beginning of the sweep to *ν*_0_ = 1/*N* = 5⋅10^−5^ for hard sweeps and *ν*_0_ ∈ {0.001, 0.02} for soft sweeps, corresponding to 20–400 carrier haplotypes at the onset of selection.

In comparing the performance of PreCIOSS against iHS, we used the software *selscan* [78] to compute iHS scores.

To investigate the performance of HAF scores on human populations, we used a popular demographic model (S11 Fig) with parameters suggested from Gravel et al. (2011) [50]. Among the different properties, the model assumes an out-of-Africa migration at 51 kya, and a European, East Asian split 23 kya. The European Asian split was accompanied by a bottleneck event that reduced the effective population sizes of the European and Asian populations, and was followed by an exponential growth in these populations. We used *msms* to simulate populations according to this model.

In modeling selection, we partitioned the onset of selection into two epochs: ‘pre-bottleneck’ events between 51 kya and 23 kya, and 23 kya, and ‘post-bottleneck’ epoch between 23 kya, and the current generation. For each epoch, we picked 10000 times for onset of selection chosen uniformly from the time interval, and performed forward simulations with a sample size of *n* = 200. Samples were chosen during the sweeps, and partitioned according to carrier allele frequency, with 200 samples randomly chosen for each bin. Samples in the pre-bottleneck epoch were simulated with *s* = 0.005 to reduce the chance of fixation, and *s* = 0.02 in the post-bottleneck epoch. The balanced accuracy measurements were done independently for the two epochs.

### Data preprocessing

We downloaded pre-phased haplotype data from the HapMap [51] project website. Both HapMap 3 [51] and HapMap 2 [79] project data were used depending on whether the causal allele was sampled or not. For LCT (rs4988235), we used 88 CEU individuals haplotypes from HapMap 3; for PSCA (rs2294008), we used 60 YRI individuals from HapMap 2; for TRPV6, 88 CEU individuals from HapMap 3; for ADH1B (rs1229984), 90 CHB+JPT individuals from HapMap 2; and, for EDAR (rs3827760), we chose 90 CHB+JPT individuals from HapMap 2. The number of phased haplotypes was twice the number of individuals in each case.

We downloaded Chimpanzee genome alignments [52] to identify the ancestral allele. A total of ∼93% of the sites analyzed had were covered by the Chimpanzee data. For these sites, we set the ancestral allele to the Chimpanzee allele, and we discarded sites that were not covered.

### Software

The PreCIOSS software is available from the website http://bix.ucsd.edu/projects/precioss/.

## Supporting Information

S1 Fig HAF scores in neutrally evolving constant-sized populations. The distribution of 4 × 10^6^ 1-HAF scores aggregated from 20000 population samples (each of *n* = 200 haplotypes) simulated under a standard coalescent model without recombination. Plugging the simulation parameters *θ* = 48, *n* = 200 into Eqs (3) or (7) give an expected 1-HAF score of 4776. The observed mean 1-HAF score is 4786 ± 3956 with no recombination (*ρ* = 0), and 4780 ± 1684 with *ρ* = 25 (blue line).
(TIF)

S2 Fig HAF scores in neutrally evolving exponentially growing populations. The distribution of 4 × 10^6^ 1-HAF scores aggregated from 20000 population samples (each of *n* = 200 haplotypes) simulated under a coalescent model of exponential growth without recombination. Computing the conditional expectation as described in Eq (12) with the simulation parameters (*θ* = 48, *n* = 200, *α* = 80) gives 126.9. The observed mean 1-HAF score is 128.0 with *ρ* = 0 (red line), and 127.4 with *ρ* = 25 (blue line).
(TIF)

S3 Fig HAF scores for a range of simulation parameters. Each empirical test is the average of 1000 trials. (A) Empirical mean and theoretical expected ℓ-HAF scores for a fixed size population (ℓ ∈ {1, 2, 3, 4}, *θ* ∈ {24, 48}, *ρ* = 0). (B) Empirical mean and theoretical expected 1-HAF scores for an exponentially growing population (*α* ∈ {0, 30, 60, 80}, *θ* ∈ {24, 48}, *ρ* = 0). (C) Theoretical expected 1-HAF scores (computed assuming *ρ* = 0) compared against empirical means of 1-HAF scores from samples with different recombination rates (*ρ* ∈ {0, 25, 50}, *θ* ∈ {24, 48}). (D) Interestingly, higher recombination rates reduce the variance in 1-HAF estimates. In the three green curves for *θ* = 24 (and in the three red curves for *θ* = 48), the variation from the expected value (blue) decreases as *ρ* increases. Rate *ρ* = 0 (dotted) has the most variation; *ρ* = 25 (dashed) has less; and *ρ* = 50 (solid) has the least. The theoretical values are based on (A) Eqs (3) and (S22), (B) Eq (12), and (C, D) Eq (4).
(TIF)

S4 Fig Distribution of normalized ℓ-HAF scores (ℓ-HAF^1/ℓ^). Results are based on simulated samples of size *n* = 200 drawn from a larger population size of neutrally evolving haploid population with *N* = 20000 (*θ* = 48, *ρ* = 0, *α* = 0). The green line marks the sample mean of the ℓ^th^ root of ℓ-HAF, while the red dashed line marks the ℓ^th^ root of the sample mean of ℓ-HAF. The latter matches the blue dotted line, which marks the theoretically computed value of (𝔼[ℓ-HAF])^1/ℓ^, using Eq. (S22). As ℓ increases, the high frequency mutations dominate the normalized ℓ-HAF score. The distribution becomes more left-skewed and has generally smaller values (upper bound of range approaching *n* − 1), with reduced variance.
(TIF)

S5 Fig Schematic of HAF score dynamics in an exponentially growing population with current population size *N* = 20000, population-scaled growth rate *α* = 80, and population-scaled mutation rate *θ* = 48. The population is under selection with *s* = 0:05. See Fig 2 for an explanation of the conventions used.
(TIF)

S6 Fig Schematic of HAF score dynamics in a population undergoing a soft sweep due to standing variation with *ν*_0_ = 0:002. Samples were simulated with *θ* = 48, *n* = 200, *s* = 0:05, and *ρ* ∈ {0, 25}. See Fig 2 for an explanation of the conventions used.
(TIF)

S7 Fig Recovery of HAF scores after a selective sweep. Each violin shows the Gaussian kernel density estimation (KDE) of 1-HAF scores in populations sampled at regular time intervals following the fixation of a selective sweep. All individuals at this stage are carriers of the favored allele. A standard box plot is overlaid on each violin. The horizontal dotted line represents the neutral expected value. At each time point, HAF scores were computed from 1000 simulations with *msms* [47], each with *n* = 200 haplotypes undergoing a hard sweep, with parameters *N* = 20000, *θ* = 48, *ρ* = 25, *n* = 200. At each time point, box plots marking 25^th^, 50^th^, and 75^th^ percentiles were computed for the 1000 × 200 HAF scores, with an asterisk marking the mean.
(TIF)

S8 Fig Predicting carriers of hard sweeps. Balanced accuracy of PreCIOSS in populations undergoing hard sweeps. Balanced accuracy is shown for each allele frequency bin as a standard box plot computed over 200 samples for each frequency bin, and each parameter set in S1 Table.
(TIF)

S9 Fig Balanced accuracy with different positions of favored allele. In each panel, 200 samples were simulated (*N* = 20000, *n* = 200, *θ* = 48, *ρ* = 25) while undergoing a hard sweep (*s* = 0.01) in a 50 kb window. Each panel shows balanced accuracy for a different position of the favored allele within the window, as the position varies from 0 to 25 kb.
(TIF)

S10 Fig Balanced accuracy variation with different positions of favored allele: summary statistics. In each case, 5000 samples were simulated (*N* = 20000, *n* = 200, *θ* = 48, *ρ* = 25) while undergoing a hard sweep (*s* = 0.01) in a 50 kb window. The mean, median and standard deviation of balanced accuracy of PreCIOSS was measured with the favored allele at the start of the window (0 kb, in blue) and at the middle of the window (25 kb, in red).
(TIF)

S11 Fig A model of human demography described by Gravel et al. (2011) [50, Fig 4, Table 2]. The model assumes an out-of-Africa split at time *T*_*B*_, with a bottleneck that reduced the effective population from *N*_Af_ to *N*_*B*_, allowing for migrations at rate *m*_Af-B_. The African population stays constant at *N*_Af_ up to the present generation. The model assumes a second split between European and Asian populations at time *T*_EuAs_, with a bottleneck reducing the Asian and European populations to *N*_As0_ and *N*_Eu0_ respectively. The bottleneck was followed by exponential growth at rates *r*_As_ and *r*_Eu_, as well as migrations among all three sub-populations, leading to current populations from which Asian (CHB+JPT), European (CEU), and Africans (YRI) individuals were sampled.
(TIF)

S12 Fig Predicting carriers of the PSCA sweep in CHB. (A) Haplotype 1-HAF scores in a 50 kb window centered at the favored site. (B) Balanced classification accuracy (black) and −log_2_(*P*) values (blue) as function of window size around the favored allele. *P*-values are for Wilcoxon rank sum tests rejecting the null hypothesis of identically distributed 1-HAF scores among carriers and non-carriers. The red circle indicates the balanced accuracy obtained for the 50 kb window shown on the left. As with the YRI population, we achieve high classification accuracy when considering ∼100 kb window centered at the favored allele. But unlike in YRI, we see a sharp decline in both accuracy and −log_2_(*P*) values beginning at larger distances from the favored allele. See Fig 7 for further details on the conventions used.
(TIF)

S13 Fig The coalescence of a sample of *n* individuals to their most recent common ancestor MRCA^all^, during a hard sweep. We assume that the current time has *νn* carriers of the favored allele. These coalesce to MRCA^car^ in *T* ^car^ generations. From that point, the coalescence to MRCA^all^ is governed by neutral coalescent theory. *T*(*k*) is time to MRCA of *k* randomly chosen haplotypes in a neutrally evolving population.
(TIF)

S14 Fig Partitioning the SNP matrix A of a sample of *n* individuals. (TIF)

S15 FigDynamics of expected 1-HAF score during a selective sweep.For each (*θ*, *n*, *ν*) with *θ* ∈ {24, 48}, *n* ∈ {100, 200, 300, 400}, $\nu \in \{\frac{1}{n},\frac{2}{n},\ldots ,\frac{n-1}{n}\}$, *s* = 0.08, and *N* = 2000, we did 1500 trials. We plotted the mean value of (1-HAF)/(*θn*) as a function of *ν*, for both carriers and non-carriers, and compared against the theoretical expected value. The expected value of (1-HAF)/(*nθ*) lies somewhere between the blue and red curves. The mean values may range over the whole distribution (and are not constrained by the blue and red curves) but tend to vary around the expected value.(TIF)

S1 Table Simulation parameter sets used for generating S8 Fig. In simulations B through E, we changed one parameter (in boldface) at a time vs. simulation A.
(PDF)

S1 Text Mathematical derivations of HAF score expected values, peak scores, and dynamics. (PDF)

S1 Source Code PreCIOSS. ZIP file with source code for PreCIOSS. See the project website for the latest version: http://bix.ucsd.edu/projects/precioss/
(ZIP)
