# Correction: Predicting Carriers of Ongoing Selective Sweeps without Knowledge of the Favored Allele

**DOI:** 10.1371/journal.pgen.1006472

**Published:** 2016-11-30

**Authors:** Roy Ronen, Glenn Tesler, Ali Akbari, Shay Zakov, Noah A. Rosenberg, Vineet Bafna

In panel B of Fig 1, the two haplotypes on the right have incorrect scores of 17. The correct scores of these haplotypes are 18. Please view the correct Fig 1 here.

**Fig 1:**
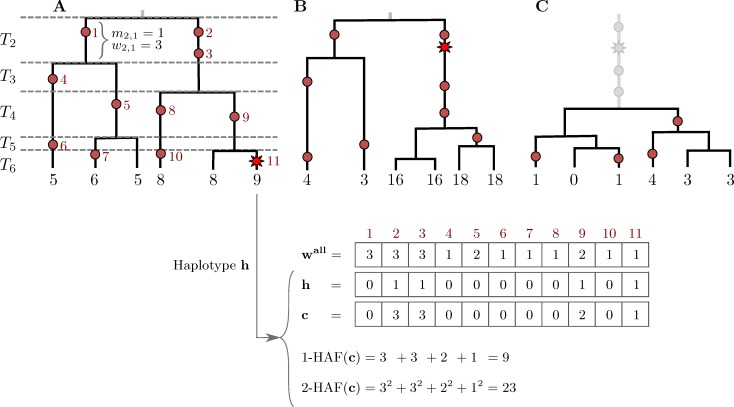
The HAF score. Genealogies of three samples (*n* = 6) progressing through a selective sweep, from left to right. Neutral mutations are shown as red circles, and are numbered in red; the favored allele is shown as a red star. The 1-HAF score of each haplotype is shown below its corresponding leaf, in black. For the rightmost haplotype in (A), the binary haplotype vector **h** is shown along with its HAF-vector **c**, and 1-HAF and 2-HAF scores. Vector **w**^all^ lists the frequencies of all mutations. (A) The favored allele appears on a single haplotype. At this point in time, both the genealogy and the HAF score distribution are largely neutral. Coalescence times (*T*_2_, …, *T*_6_) are shown on the left, where *T*_*k*_ spans the epoch with exactly *k* lineages. (B) Carriers of the favored allele are distinguished by high HAF scores (in large part due to the long branch of high-frequency hitchhiking variation); non-carriers have low HAF scores. (C) After fixation, there is a sharp loss of diversity causing low HAF scores across the sample.
