# Supplementary figures and images for: Predicting Carriers of Ongoing Selective Sweeps without Knowledge of the Favored Allele
Source: PLoS Genet. 2015 Sep 24;11(9):e1005527. doi: 10.1371/journal.pgen.1005527 (PMC4581834; doi:10.1371/journal.pgen.1005527)

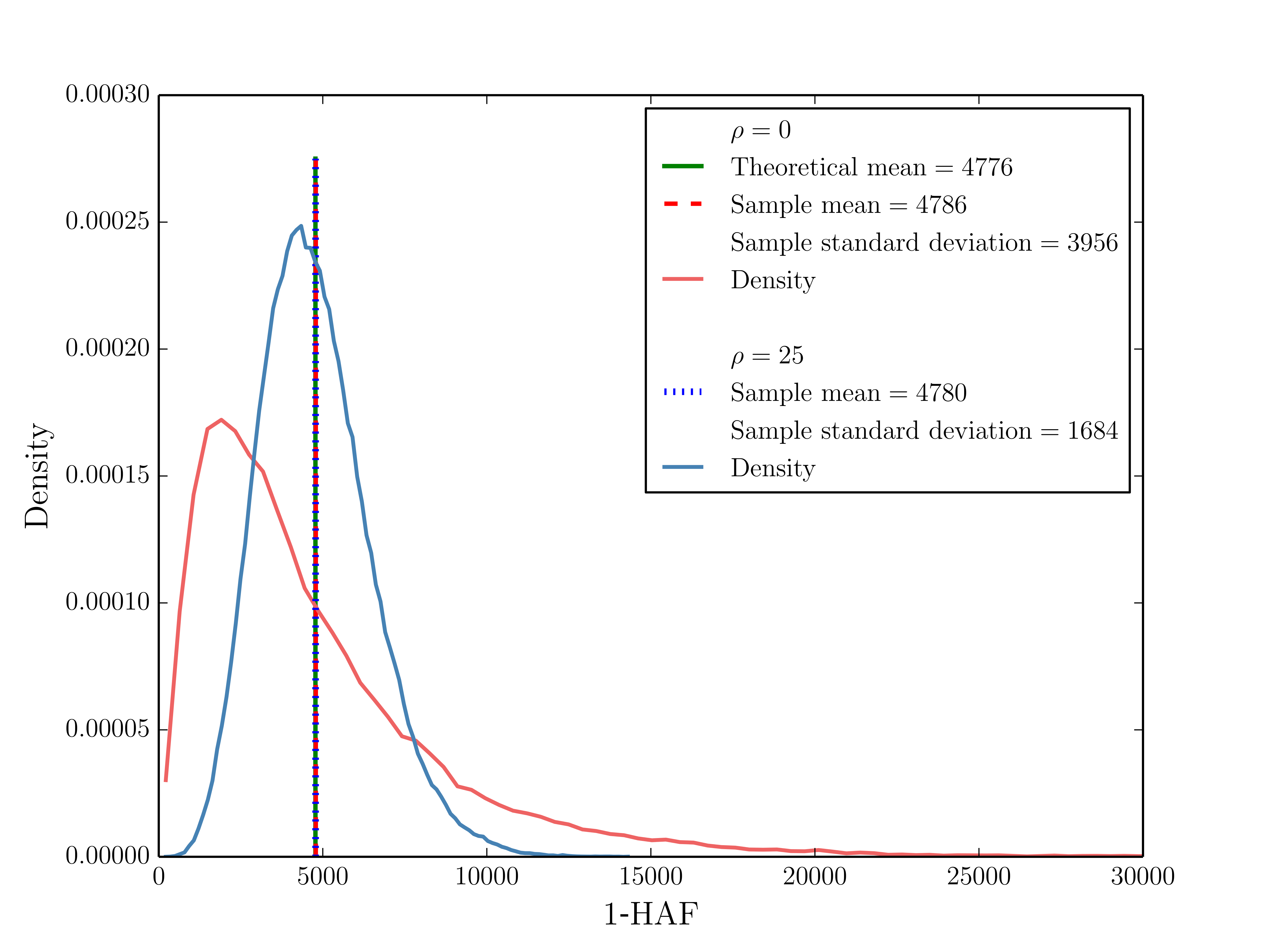

Supplement: S1 Fig — The distribution of 4 × 10^6^ 1-HAF scores aggregated from 20000 population samples (each of n = 200 haplotypes) simulated under a standard coalescent model without recombination. Plugging the simulation parameters θ = 48, n = 200 into Eqs (3) or (7) give an expected 1-HAF score of 4776. The observed mean 1-HAF score is 4786 ± 3956 with no recombination (ρ = 0), and 4780 ± 1684 with ρ = 25 (blue line). (TIF) [file pgen.1005527.s001.tif]

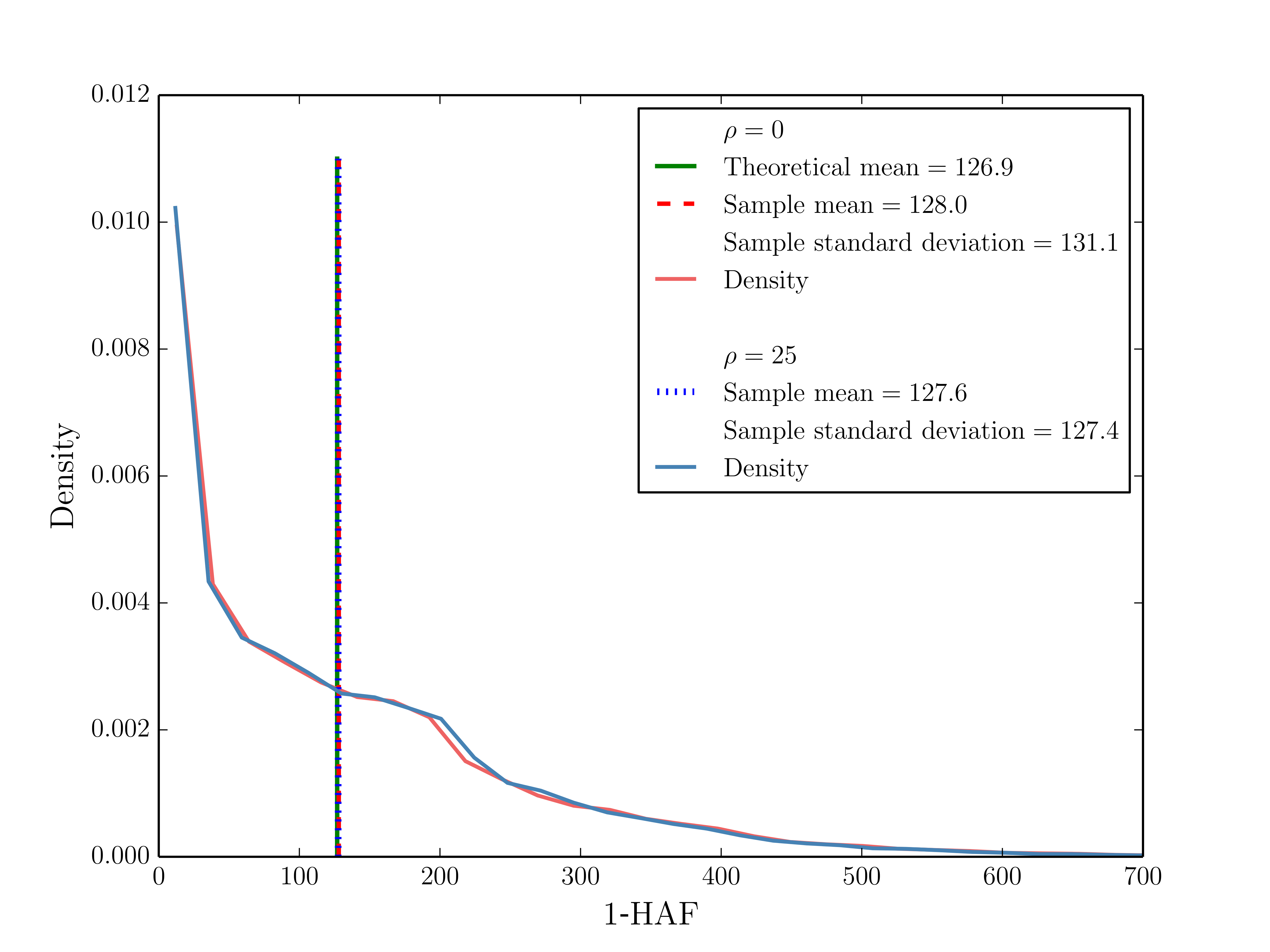

Supplement: S2 Fig — The distribution of 4 × 10^6^ 1-HAF scores aggregated from 20000 population samples (each of n = 200 haplotypes) simulated under a coalescent model of exponential growth without recombination. Computing the conditional expectation as described in Eq (12) with the simulation parameters (θ = 48, n = 200, α = 80) gives 126.9. The observed mean 1-HAF score is 128.0 with ρ = 0 (red line), and 127.4 with ρ = 25 (blue line). (TIF) [file pgen.1005527.s002.tif]

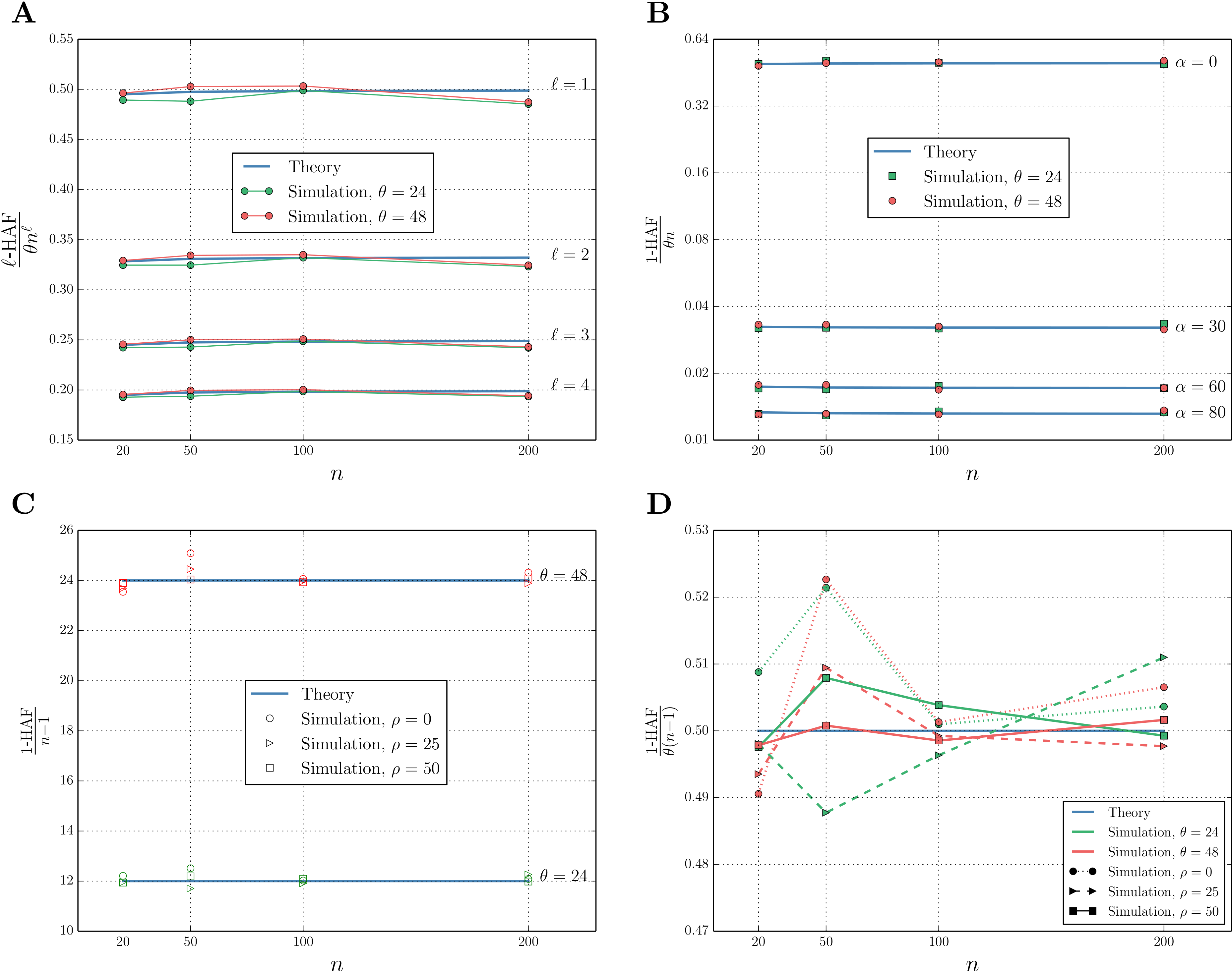

Supplement: S3 Fig — Each empirical test is the average of 1000 trials. (A) Empirical mean and theoretical expected ℓ-HAF scores for a fixed size population (ℓ ∈ {1, 2, 3, 4}, θ ∈ {24, 48}, ρ = 0). (B) Empirical mean and theoretical expected 1-HAF scores for an exponentially growing population (α ∈ {0, 30, 60, 80}, θ ∈ {24, 48}, ρ = 0). (C) Theoretical expected 1-HAF scores (computed assuming ρ = 0) compared against empirical means of 1-HAF scores from samples with different recombination rates (ρ ∈ {0, 25, 50}, θ ∈ {24, 48}). (D) Interestingly, higher recombination rates reduce the variance in 1-HAF estimates. In the three green curves for θ = 24 (and in the three red curves for θ = 48), the variation from the expected value (blue) decreases as ρ increases. Rate ρ = 0 (dotted) has the most variation; ρ = 25 (dashed) has less; and ρ = 50 (solid) has the least. The theoretical values are based on (A) Eqs (3) and (S22), (B) Eq (12), and (C, D) Eq (4). (TIF) [file pgen.1005527.s003.tif]

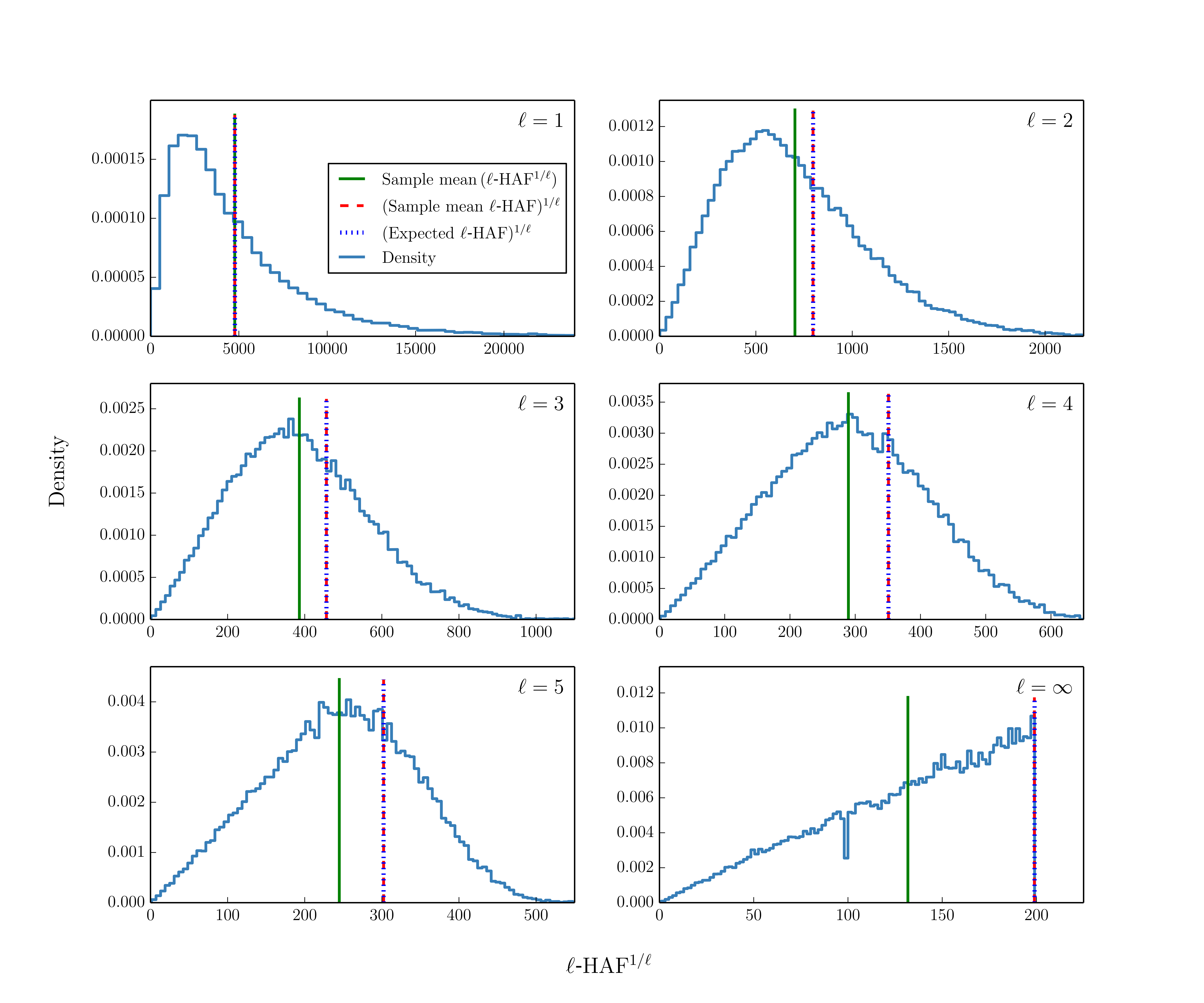

Supplement: S4 Fig — Results are based on simulated samples of size n = 200 drawn from a larger population size of neutrally evolving haploid population with N = 20000 (θ = 48, ρ = 0, α = 0). The green line marks the sample mean of the ℓth root of ℓ-HAF, while the red dashed line marks the ℓth root of the sample mean of ℓ-HAF. The latter matches the blue dotted line, which marks the theoretically computed value of (𝔼[ℓ-HAF])1/ℓ, using Eq. (S22). As ℓ increases, the high frequency mutations dominate the normalized ℓ-HAF score. The distribution becomes more left-skewed and has generally smaller values (upper bound of range approaching n − 1), with reduced variance. (TIF) [file pgen.1005527.s004.tif]

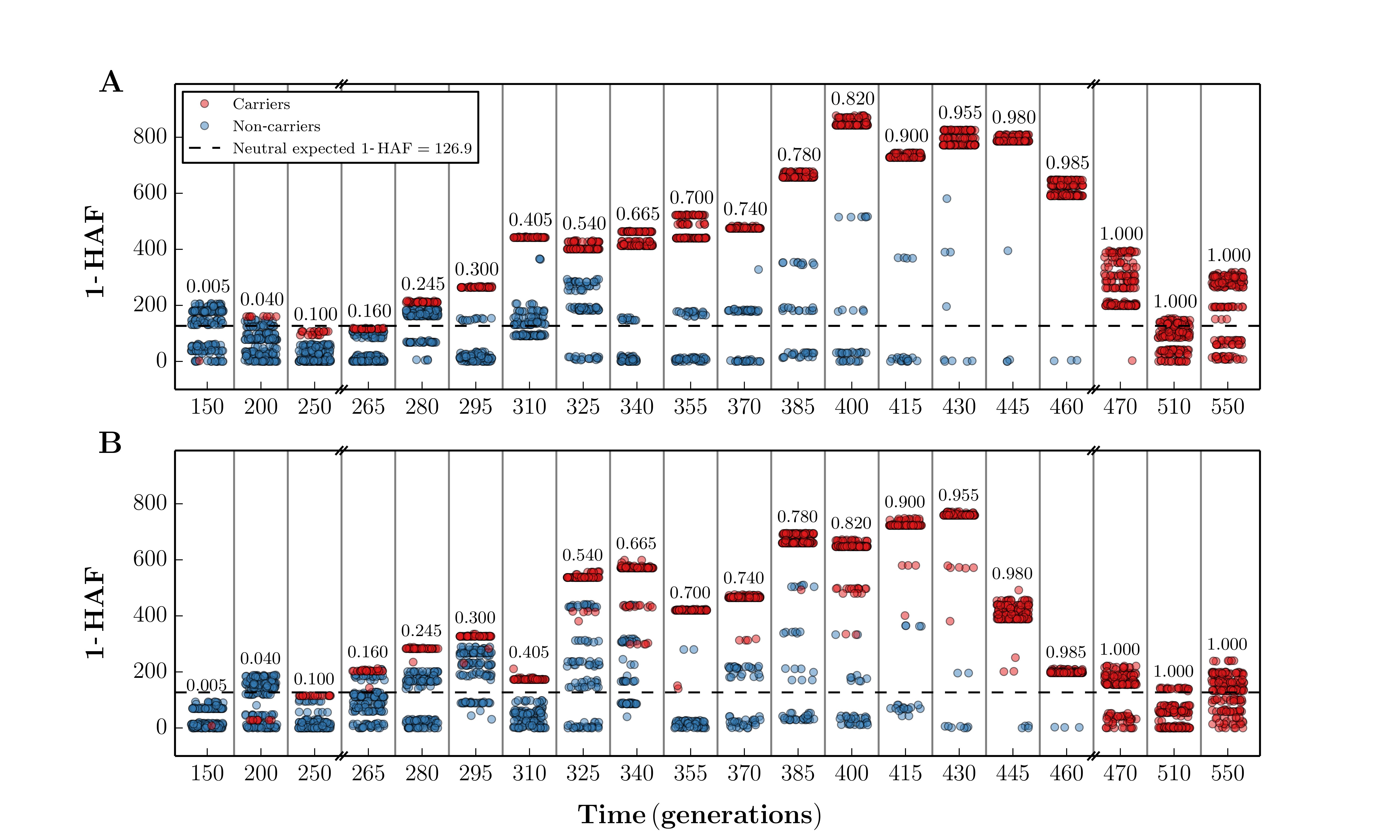

Supplement: S5 Fig — The population is under selection with s = 0:05. See Fig 2 for an explanation of the conventions used. (TIF) [file pgen.1005527.s005.tif]

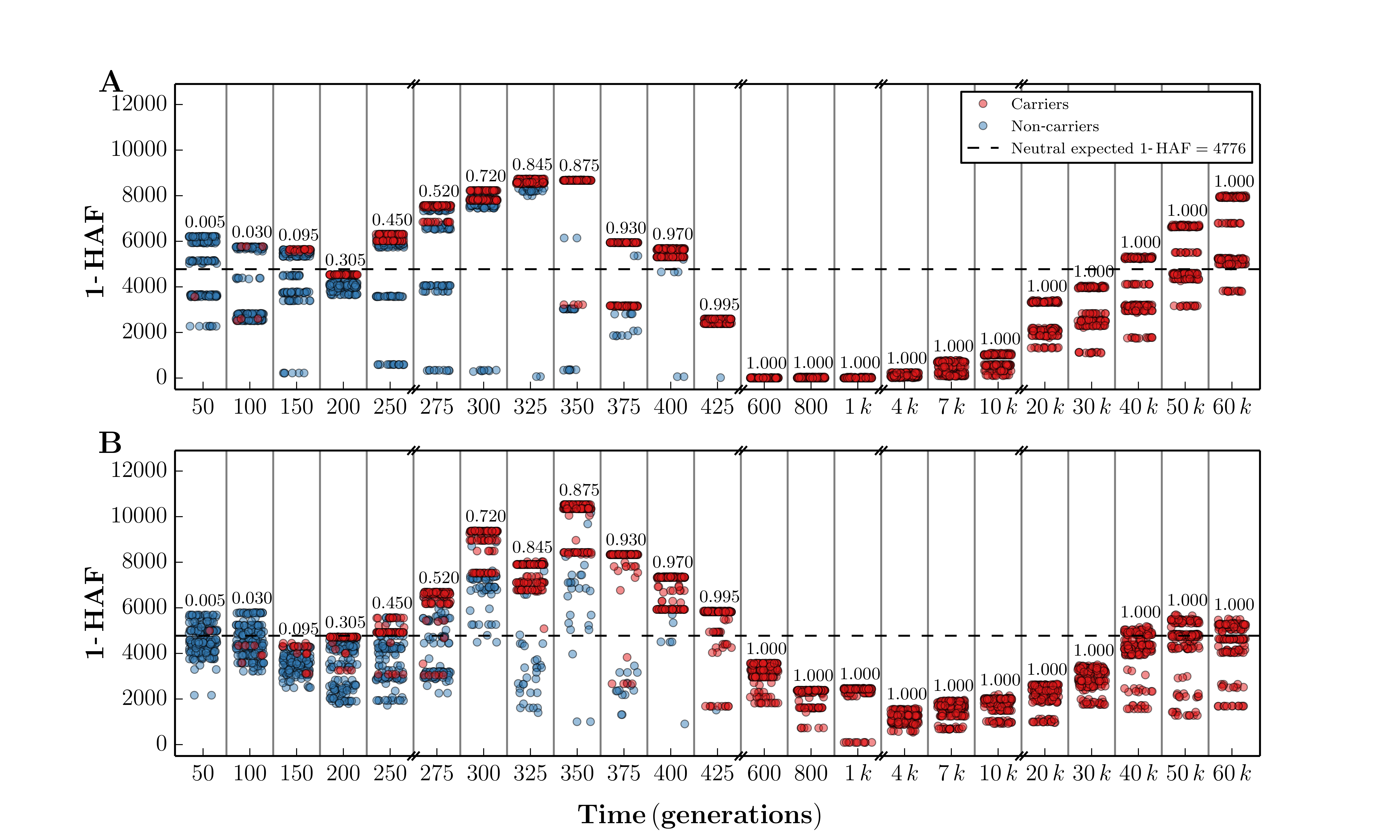

Supplement: S6 Fig — Samples were simulated with θ = 48, n = 200, s = 0:05, and ρ ∈ {0, 25}. See Fig 2 for an explanation of the conventions used. (TIF) [file pgen.1005527.s006.tif]

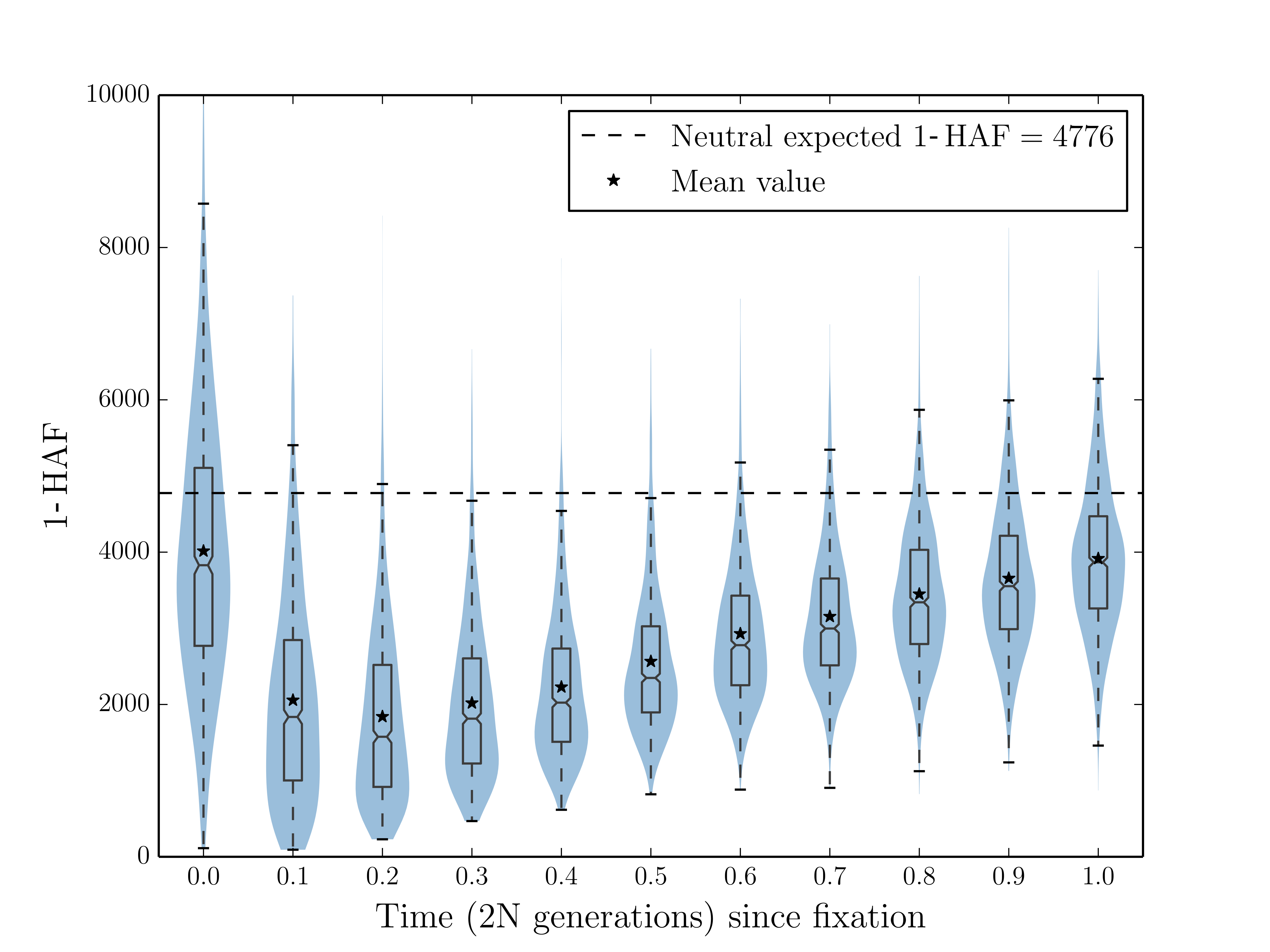

Supplement: S7 Fig — Each violin shows the Gaussian kernel density estimation (KDE) of 1-HAF scores in populations sampled at regular time intervals following the fixation of a selective sweep. All individuals at this stage are carriers of the favored allele. A standard box plot is overlaid on each violin. The horizontal dotted line represents the neutral expected value. At each time point, HAF scores were computed from 1000 simulations with msms [47], each with n = 200 haplotypes undergoing a hard sweep, with parameters N = 20000, θ = 48, ρ = 25, n = 200. At each time point, box plots marking 25th, 50th, and 75th percentiles were computed for the 1000 × 200 HAF scores, with an asterisk marking the mean. (TIF) [file pgen.1005527.s007.tif]

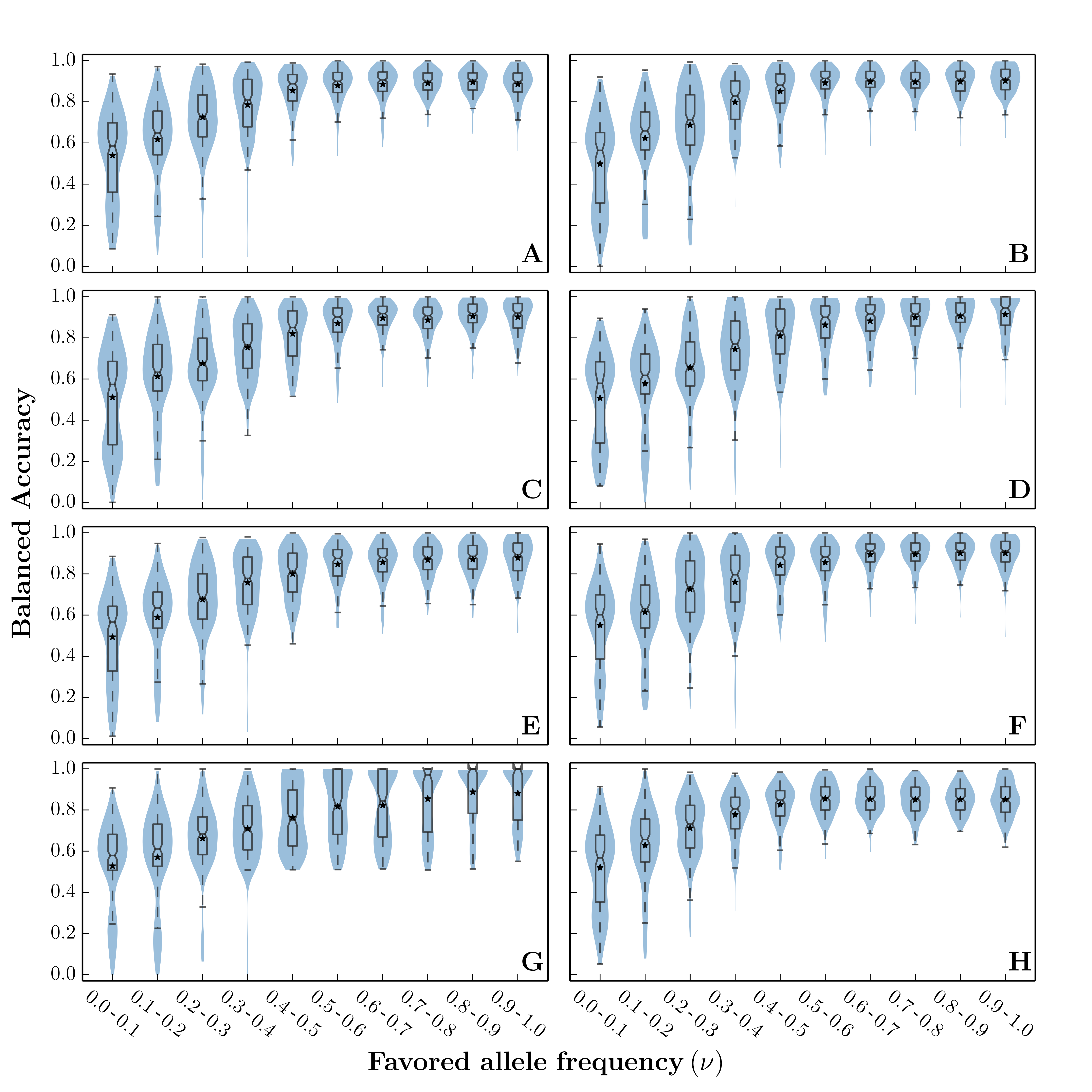

Supplement: S8 Fig — Balanced accuracy of PreCIOSS in populations undergoing hard sweeps. Balanced accuracy is shown for each allele frequency bin as a standard box plot computed over 200 samples for each frequency bin, and each parameter set in S1 Table. (TIF) [file pgen.1005527.s008.tif]

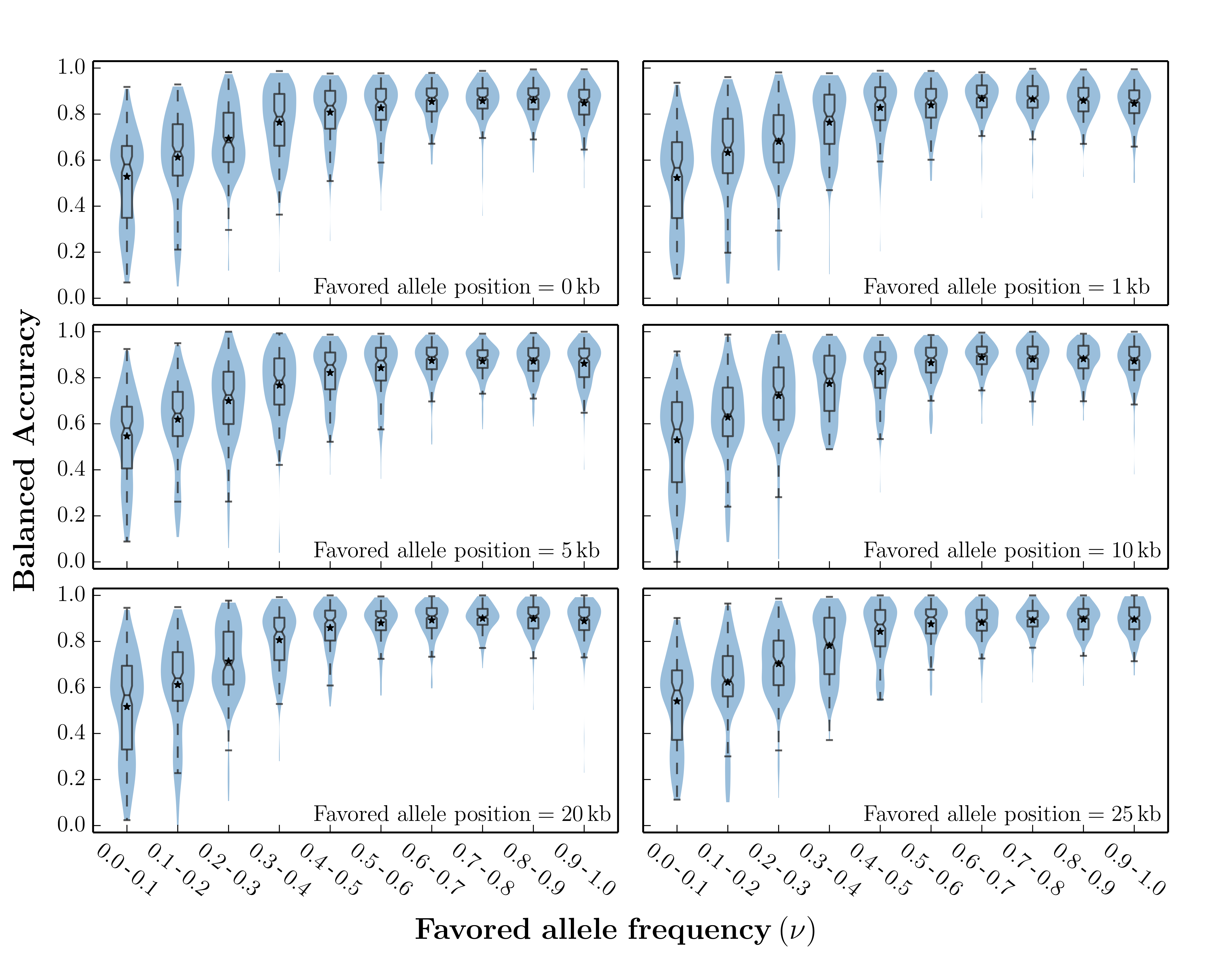

Supplement: S9 Fig — In each panel, 200 samples were simulated (N = 20000, n = 200, θ = 48, ρ = 25) while undergoing a hard sweep (s = 0.01) in a 50 kb window. Each panel shows balanced accuracy for a different position of the favored allele within the window, as the position varies from 0 to 25 kb. (TIF) [file pgen.1005527.s009.tif]

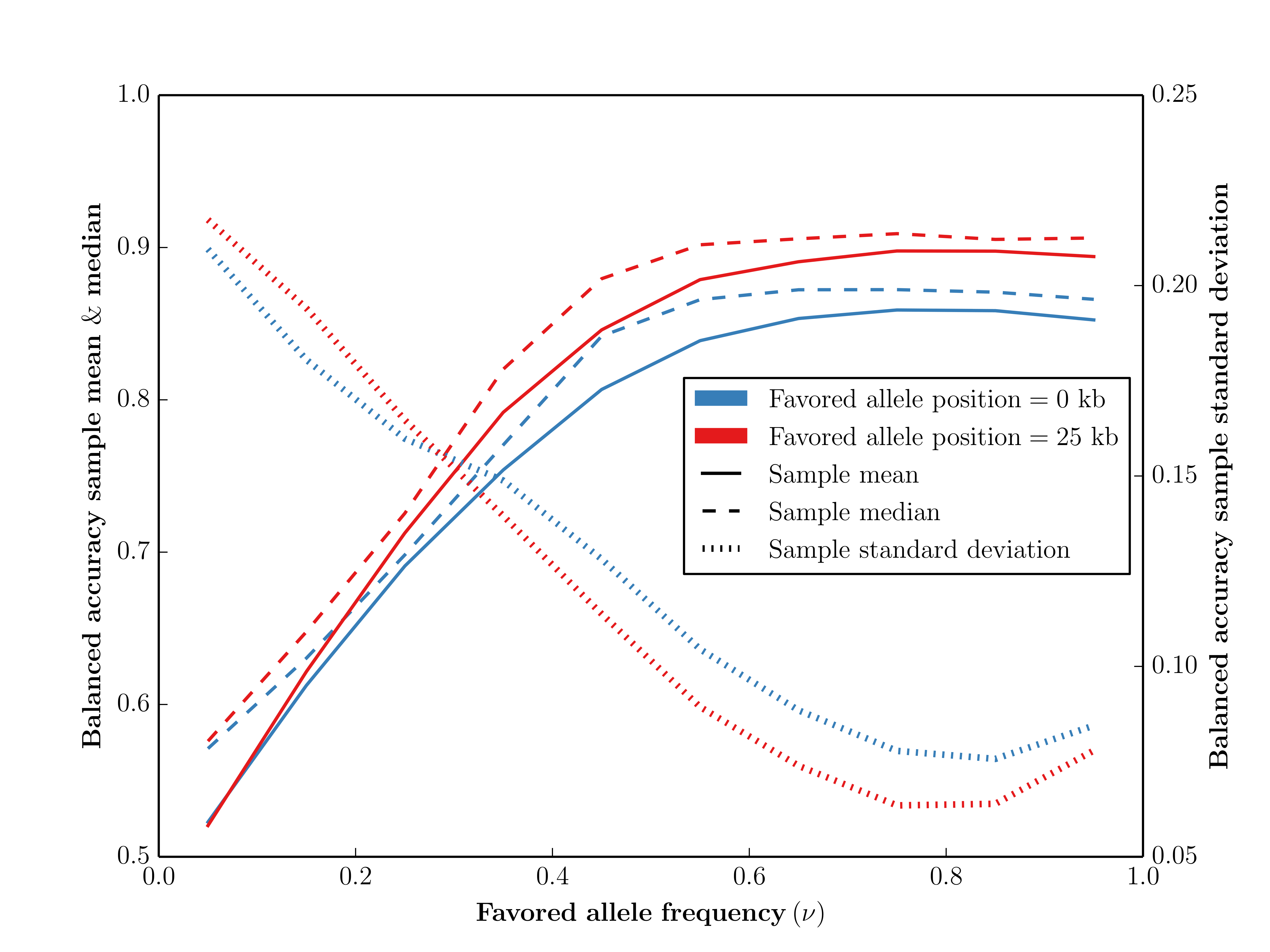

Supplement: S10 Fig — In each case, 5000 samples were simulated (N = 20000, n = 200, θ = 48, ρ = 25) while undergoing a hard sweep (s = 0.01) in a 50 kb window. The mean, median and standard deviation of balanced accuracy of PreCIOSS was measured with the favored allele at the start of the window (0 kb, in blue) and at the middle of the window (25 kb, in red). (TIF) [file pgen.1005527.s010.tif]

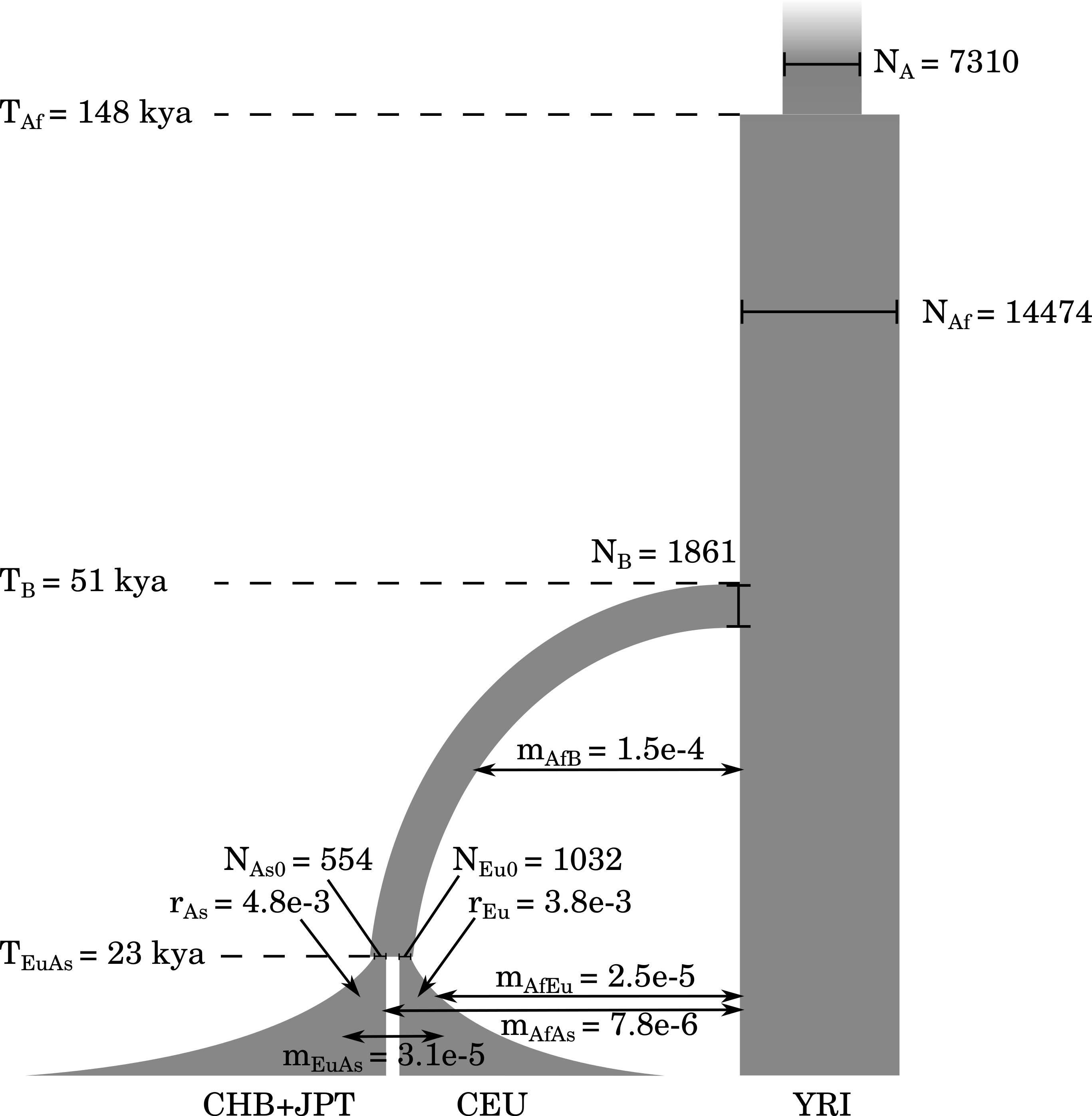

Supplement: S11 Fig — The model assumes an out-of-Africa split at time T B, with a bottleneck that reduced the effective population from N Af to N B, allowing for migrations at rate m Af-B. The African population stays constant at N Af up to the present generation. The model assumes a second split between European and Asian populations at time T EuAs, with a bottleneck reducing the Asian and European populations to N As0 and N Eu0 respectively. The bottleneck was followed by exponential growth at rates r As and r Eu, as well as migrations among all three sub-populations, leading to current populations from which Asian (CHB+JPT), European (CEU), and Africans (YRI) individuals were sampled. (TIF) [file pgen.1005527.s011.tif]

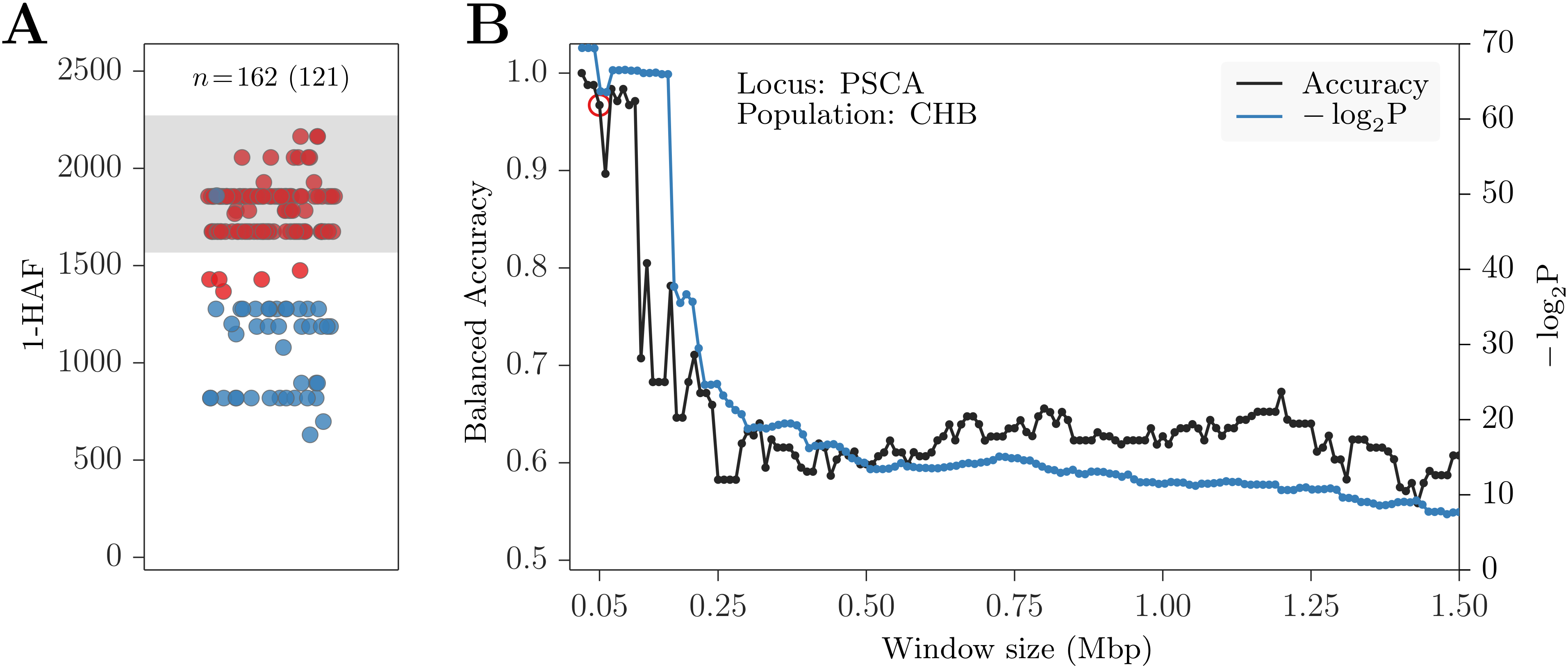

Supplement: S12 Fig — (A) Haplotype 1-HAF scores in a 50 kb window centered at the favored site. (B) Balanced classification accuracy (black) and −log2(P) values (blue) as function of window size around the favored allele. P-values are for Wilcoxon rank sum tests rejecting the null hypothesis of identically distributed 1-HAF scores among carriers and non-carriers. The red circle indicates the balanced accuracy obtained for the 50 kb window shown on the left. As with the YRI population, we achieve high classification accuracy when considering ∼100 kb window centered at the favored allele. But unlike in YRI, we see a sharp decline in both accuracy and −log2(P) values beginning at larger distances from the favored allele. See Fig 7 for further details on the conventions used. (TIF) [file pgen.1005527.s012.tif]

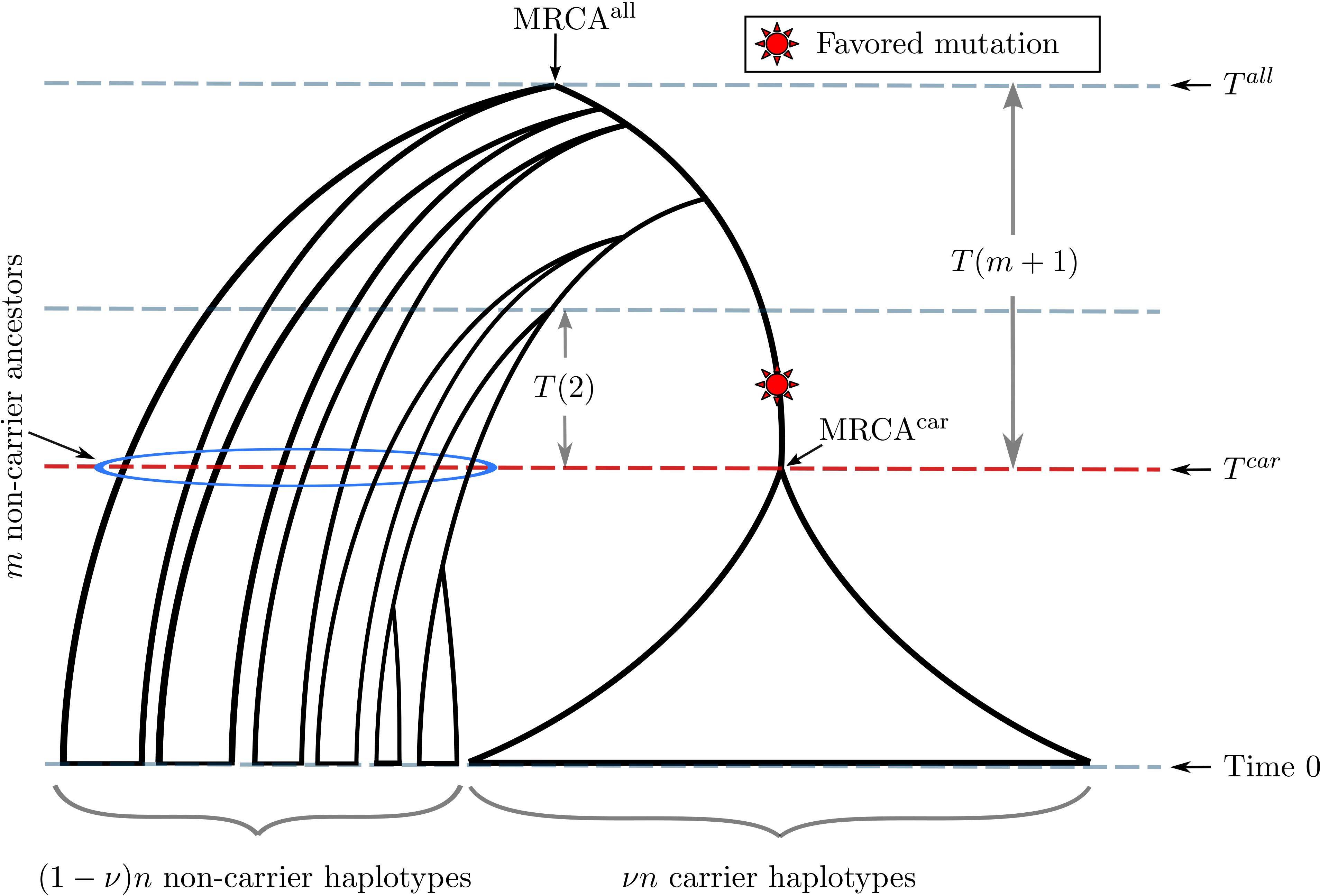

Supplement: S13 Fig — We assume that the current time has νn carriers of the favored allele. These coalesce to MRCAcar in T car generations. From that point, the coalescence to MRCAall is governed by neutral coalescent theory. T(k) is time to MRCA of k randomly chosen haplotypes in a neutrally evolving population. (TIF) [file pgen.1005527.s013.tif]

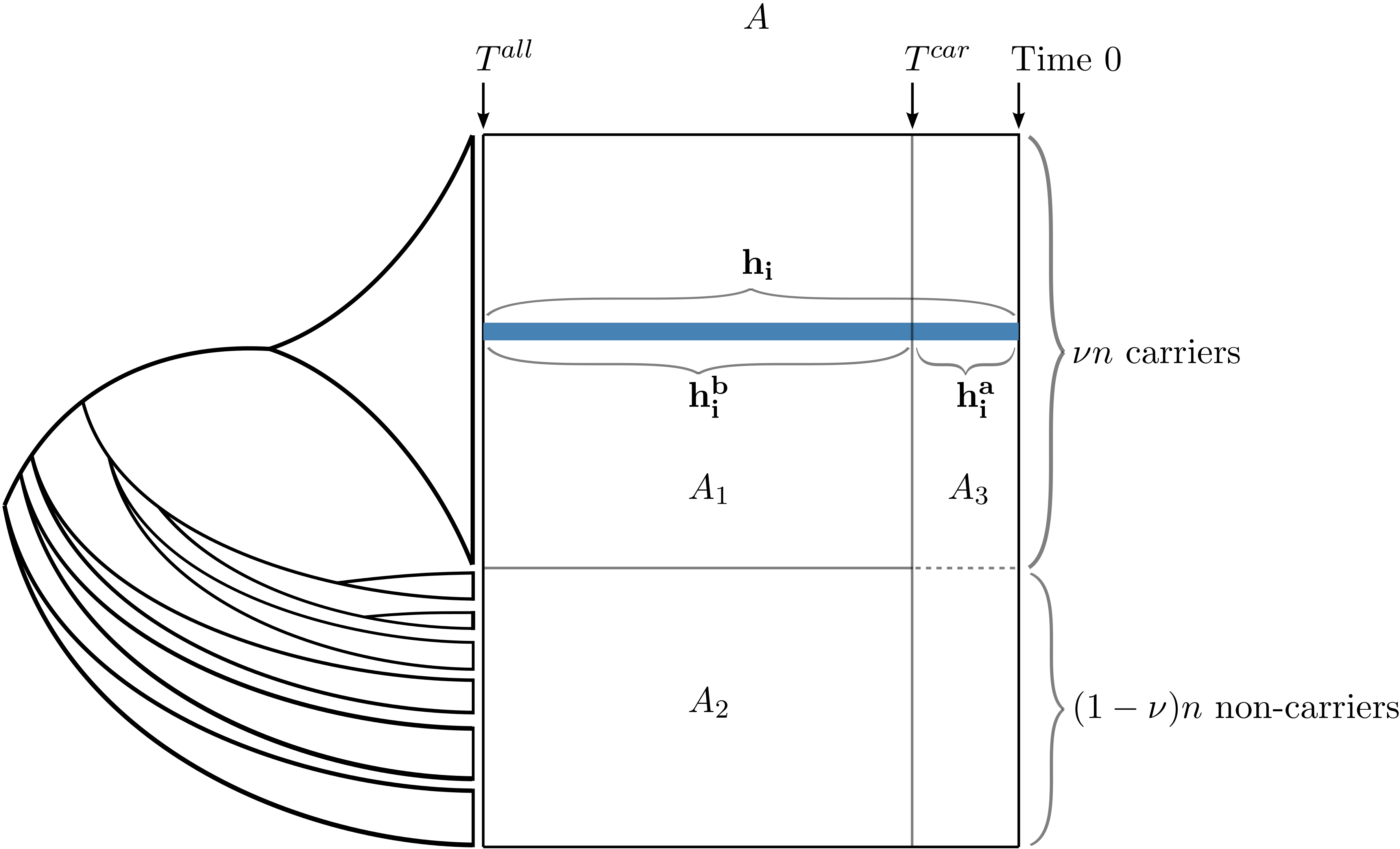

Supplement: S14 Fig — (TIF) [file pgen.1005527.s014.tif]

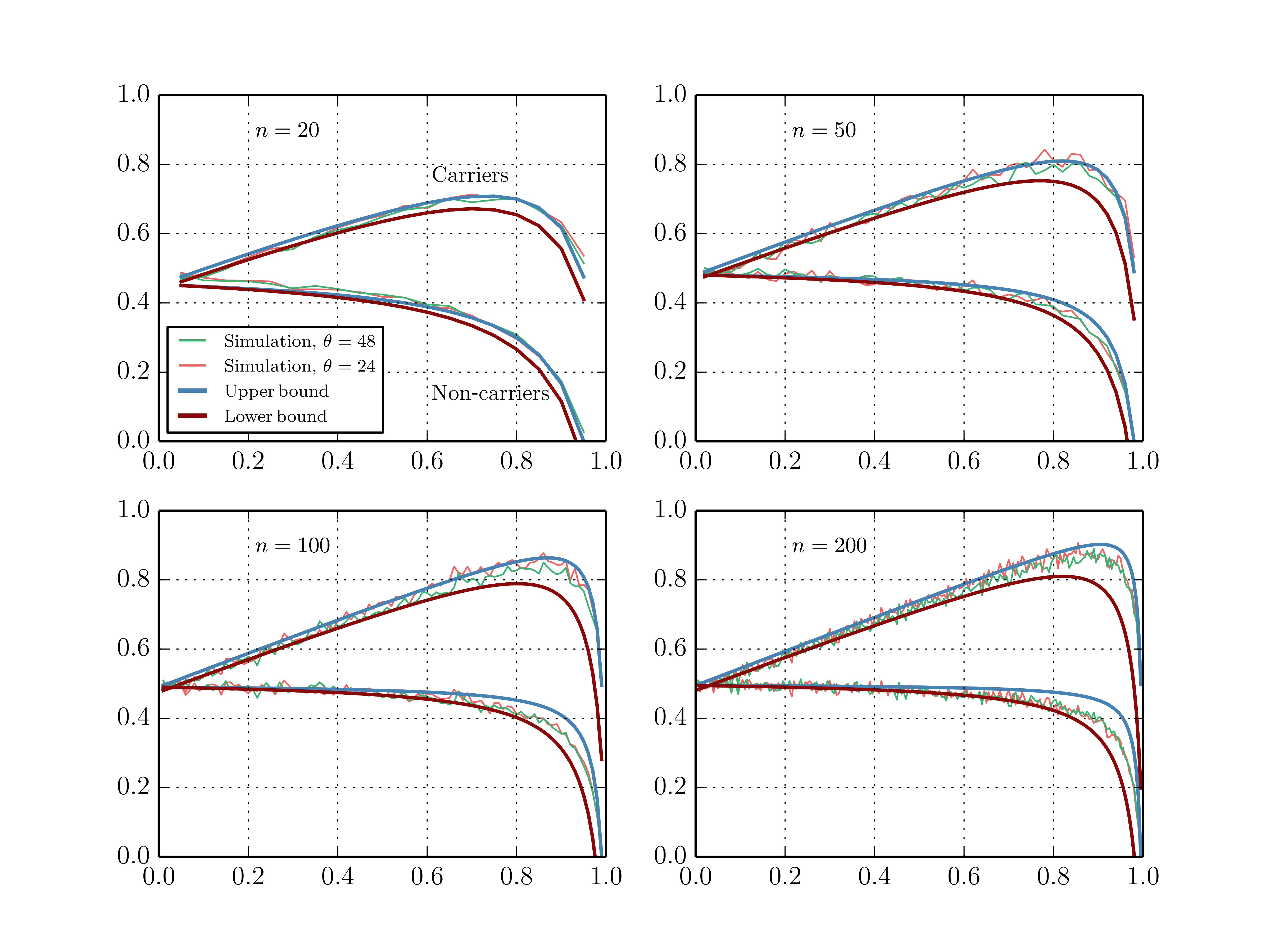

Supplement: S15 Fig — For each (θ, n, ν) with θ ∈ {24, 48}, n ∈ {100, 200, 300, 400}, ν∈{1n,2n,…,n-1n}, s = 0.08, and N = 2000, we did 1500 trials. We plotted the mean value of (1-HAF)/(θn) as a function of ν, for both carriers and non-carriers, and compared against the theoretical expected value. The expected value of (1-HAF)/(nθ) lies somewhere between the blue and red curves. The mean values may range over the whole distribution (and are not constrained by the blue and red curves) but tend to vary around the expected value. (TIF) [file pgen.1005527.s015.tif]

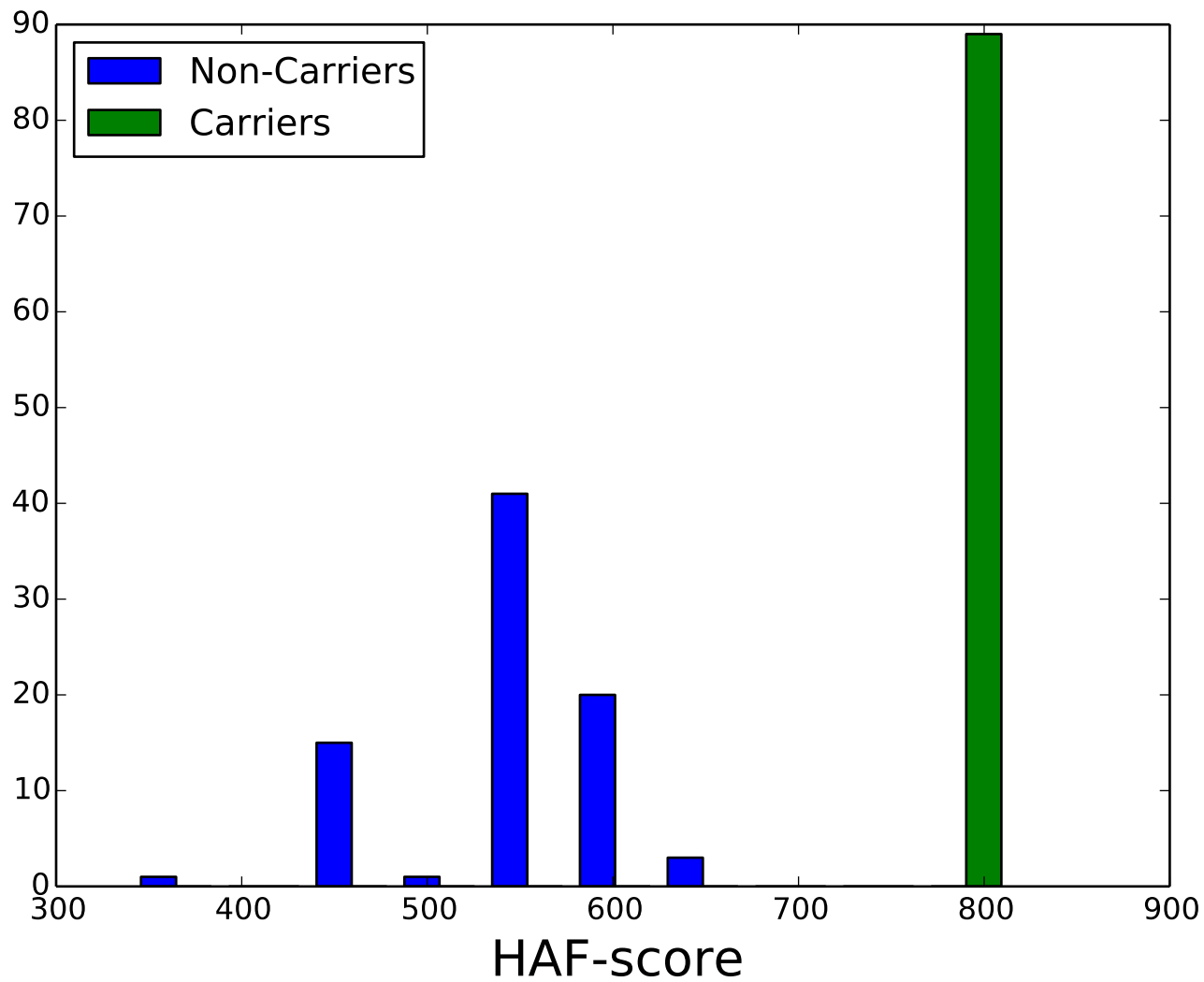

Supplement: S1 Source Code — ZIP file with source code for PreCIOSS. See the project website for the latest version: http://bix.ucsd.edu/projects/precioss/ (ZIP) [file pgen.1005527.s018.zip › S1_Source_Code/PreCIOSS/ch1.pdf]
