# Supplementary material for: Predicting Carriers of Ongoing Selective Sweeps without Knowledge of the Favored Allele
Source: PLoS Genet. 2015 Sep 24;11(9):e1005527. doi: 10.1371/journal.pgen.1005527 (PMC4581834; doi:10.1371/journal.pgen.1005527)
Supplement: S1 Table — In simulations B through E, we changed one parameter (in boldface) at a time vs. simulation A. (PDF) [file pgen.1005527.s016.pdf]

Parameters	s	θ	n	ρ
A	0.01	48	200	25
B	0.01	48	**100**	25
C	0.01	48	**50**	25
D	0.01	48	**20**	25
E	**0.005**	48	200	25
F	0.01	**24**	200	25
G	0.01	48	200	**0**
H	0.01	48	200	**50**

S1 Table. Simulation parameter sets used for generating S8 Fig. In simulations B through E, we changed one parameter (in boldface) at a time vs. simulation A.
