# Supplementary material for: Predicting Carriers of Ongoing Selective Sweeps without Knowledge of the Favored Allele
Source: PLoS Genet. 2015 Sep 24;11(9):e1005527. doi: 10.1371/journal.pgen.1005527 (PMC4581834; doi:10.1371/journal.pgen.1005527)
Supplement: S1 Text — (PDF) [file pgen.1005527.s017.pdf]

Predicting Carriers of Ongoing Selective Sweeps Without Knowledge of the Favored Allele — Supporting Information

Roy Ronen[1,◕], Glenn Tesler[2,◕], Ali Akbari[3,◕], Shay Zakov[4], Noah A. Rosenberg[5], Vineet Bafna[4,*]

1 Bioinformatics Graduate Program, University of California, San Diego, La Jolla, CA, USA
2 Department of Mathematics, University of California, San Diego, La Jolla, CA, USA
3 Department of Electrical & Computer Engineering, University of California, San Diego, La Jolla, CA, USA
4 Department of Computer Science & Engineering, University of California, San Diego, La Jolla, CA, USA
5 Department of Biology, Stanford University, Stanford, CA, USA

◕These authors contributed equally to this work.
*** vbafna@ucsd.edu**

S1 Text. Mathematical derivations of HAF score expected values, peak scores, and dynamics.

Derivation of the expected ℓ-HAF score.

We compute the expected value by averaging over all haplotypes in a genealogy (sum over the haplotypes and divide by n) and then averaging over all genealogies. Recall from the main text that $M_{k,i}$ and $W_{k,i}$ refer to random variables denoting the number of mutations and the frequency, respectively on the ith lineage of the kth epoch. As the genealogy of a neutrally evolving sample is independent of branch lengths [1], $M_{k,i}$ and $W_{k,i}$ are independent random variables. Recall Eq. (7):

$$\mathbb{E}[\ell\text{-HAF}] = \frac{1}{n}\sum_{k=2}^{n}\sum_{i=1}^{k}\mathbb{E}[M_{k,i}W_{k,i}^{\ell+1}] = \frac{1}{n}\sum_{k=2}^{n}\sum_{i=1}^{k}\mathbb{E}[M_{k,i}]\mathbb{E}[W_{k,i}^{\ell+1}]\,. \tag{S1}$$

Expected value of $M_{k,i}$, constant population size. Let T_k denote the duration of epoch k. For a population of constant size N, the duration T_k is exponentially distributed with rate $\binom{k}{2}/N$ (see [2]):

$$\mathbb{E}[T_k] = \frac{N}{\binom{k}{2}}\,, \tag{S2}$$

and the number of mutations on a lineage i in epoch k, denoted $M_{k,i}$, is Poisson distributed with rate μT_k. For a constant-sized population, this implies

$$\mathbb{E}[M_{k,i}] = \frac{N\mu}{\binom{k}{2}} = \frac{\theta}{k(k-1)}\,. \tag{S3}$$

Later, we will explore $\mathbb{E}[T_k]$ and $\mathbb{E}[M_{k,i}]$ for exponentially growing populations.

Rising factorials and moment calculations. The probability distribution of $W_{k,i}$, or the number of descendants of the ith lineage in epoch k, is given by [3, Eq. (14)]:

$$\Pr(W_{k,i} = w) = \frac{\binom{n-w-1}{k-2}}{\binom{n-1}{k-1}}\,, \tag{S4}$$

where $1 \le i \le k$, $2 \le k \le n$, and $1 \le w \le n-k+1$. We wish to evaluate Eq. (7) (or (S1)) for $\ell = 1$, and generally for any ℓ. This requires $\mathbb{E}[M_{k,i}]$, which we already have for constant populations, and $\mathbb{E}[W_{k,i}^{\ell}]$, which we derive next. For a positive integer ℓ, denote the *rising factorial*

$$w^{(\ell)} = w(w+1)(w+2)\cdots(w+\ell-1) \tag{S5}$$

and set $w^{(0)} = 1$. Then $W_{k,i}^{(\ell)}$ denotes the ℓth rising factorial of random variable $W_{k,i}$, while $W_{k,i}^{\ell}$ denotes the ordinary ℓ^{th} power. We will compute $\mathbb{E}[W_{k,i}^{(\ell)}]$, and use it to compute $\mathbb{E}[W_{k,i}^{\ell}]$.

Deriving $\mathbb{E}[W_{k,i}^{(\ell)}]$

Let ℓ be a nonnegative integer. We will prove that

$$\mathbb{E}\left[\frac{W_{k,i}^{(\ell)}}{\ell!}\right] = \frac{n^{(\ell)}}{k^{(\ell)}}\,, \tag{S6}$$

which is equivalent to Eq. (9) in the text. Using Eq. (S4), the expected value is the sum

$$\mathbb{E}\left[\frac{W_{k,i}^{(\ell)}}{\ell!}\right] = \sum_{w=1}^{n-k+1} \frac{\binom{n-w-1}{k-2}}{\binom{n-1}{k-1}} \cdot \frac{w^{(\ell)}}{\ell!} = \frac{1}{\binom{n-1}{k-1}} \sum_{w=1}^{n-k+1} \binom{n-w-1}{k-2} \cdot \binom{w+\ell-1}{\ell}\,. \tag{S7}$$

Here we use that rising factorials and binomial coefficients are related via $\frac{w^{(\ell)}}{\ell!} = \binom{w+\ell-1}{\ell}$.

The rightmost sum may be evaluated using a combinatorial method: we will count all $(k+\ell-1)$-element subsets of $\{1, 2, \ldots, n+\ell-1\}$ by two methods. First, it is the binomial coefficient $\binom{n+\ell-1}{k+\ell-1}$. Second, we will show that it also equals the rightmost sum in (S7).

To show this, we may order any such subset and partition it into three parts, (A, b, C): A is the set of its smallest ℓ elements; b is the $(\ell+1)^{\text{th}}$ smallest element; and C is the set of the largest $k-2$ elements.

Element b must be in the range $\ell+1 \le b \le n-k+\ell+1$ to allow for ℓ smaller elements and $k-2$ larger elements. We rewrite b as $b = \ell + w$, where w is in the range $1 \le w \le n-k+1$.

Given b, we may choose A in one of $\binom{b-1}{\ell} = \binom{w+\ell-1}{\ell}$ ways and choose C in one of $\binom{n+\ell-1-b}{k-2} = \binom{n-w-1}{k-2}$ ways. Multiply these counts for a given b, and sum over all b (by summing over all w) to obtain that the total number of subsets is the rightmost sum in (S7). But we also showed the number of such subsets is $\binom{n+\ell-1}{k+\ell-1}$. Thus, (S7) evaluates to

$$\begin{aligned}\mathbb{E}\left[\frac{W_{k,i}^{(\ell)}}{\ell!}\right] &= \frac{\binom{n+\ell-1}{k+\ell-1}}{\binom{n-1}{k-1}} = \frac{(n+\ell-1)!}{(k+\ell-1)!\,(n-k)!}\Bigg/\frac{(n-1)!}{(k-1)!\,(n-k)!} \\ &= \frac{(n+\ell-1)!\,/\,(n-1)!}{(k+\ell-1)!\,/\,(k-1)!} = \frac{n^{(\ell)}}{k^{(\ell)}}\,.\end{aligned}$$

Deriving $\mathbb{E}[\ell\text{-HAF}]$ in a constant-sized population

Our derivation of $\mathbb{E}[\ell\text{-HAF}]$ consists of three main steps. We start by defining a more general form of the HAF score for an arbitrary function $f(w)$, which we denote HAF_f. We then set $f(w)$ to the rising factorial function and derive $\mathbb{E}[\text{HAF}_{w^{(\ell)}}]$. Finally, we use Stirling Numbers to convert between rising factorials and powers, and obtain $\mathbb{E}[\ell\text{-HAF}]$.

Step 1: The generalized form $\mathbb{E}[\mathbf{HAF}_f]$

Consider an arbitrary polynomial $f(w)$ (or more generally, an arbitrary function $f : \mathbb{Z} \to \mathbb{Z}$). Define the HAF_f score of a HAF vector $\mathbf{c}$ as

$$\mathrm{HAF}_f(\mathbf{c}) = \sum_j f(c_j) = \sum_{k=2}^{n} m_k f(w_k)\,. \tag{S8}$$

We now generalize the derivation of Eq. (7) to HAF_f. Sum the above equation over all haplotypes in a genealogy:

$$\begin{aligned}\sum_{v=1}^{n} \mathrm{HAF}_f(\mathbf{c}(v)) &= \sum_{k=2}^{n}\sum_{v=1}^{n} m_k(v) f(w_k(v)) \\ &= \sum_{k=2}^{n}\sum_{i=1}^{k}\sum_{j=1}^{w_{k,i}} m_{k,i} f(w_{k,i}) = \sum_{k=2}^{n}\sum_{i=1}^{k} m_{k,i} w_{k,i} \cdot f(w_{k,i})\,. \end{aligned} \tag{S9}$$

Note that in the sum over j, all summands are independent of j, so that sum is replaced by multiplying by the number of terms, $w_{k,i}$. We divide the above sum by n and then average over all genealogies to obtain

$$\mathbb{E}[\mathrm{HAF}_f] = \frac{1}{n}\sum_{k=2}^{n}\sum_{i=1}^{k} \mathbb{E}[M_{k,i} W_{k,i} f(W_{k,i})] = \frac{1}{n}\sum_{k=2}^{n}\sum_{i=1}^{k} \mathbb{E}[M_{k,i}]\mathbb{E}[W_{k,i} \cdot f(W_{k,i})]\,. \tag{S10}$$

Further, since the random variables $W_{k,i}$ for $i = 1, \ldots, k$ are identically distributed, all terms in the inner sum are the same, and may be consolidated into k times the value of the term on one branch:

$$\mathbb{E}[\mathrm{HAF}_f] = \frac{1}{n}\sum_{k=2}^{n} k \cdot \mathbb{E}[M_{k,1}]\,\mathbb{E}[W_{k,1} \cdot f(W_{k,1})]\,. \tag{S11}$$

In a constant-sized population, Eq. (S3) states that $\mathbb{E}[M_{k,i}] = \theta/(k(k-1))$. Plug this in:

$$\mathbb{E}[\mathrm{HAF}_f] = \frac{\theta}{n}\sum_{k=2}^{n} \frac{1}{k-1}\mathbb{E}[W_{k,1} \cdot f(W_{k,1})]\,. \tag{S12}$$

Step 2: The rising factorial form $\mathbb{E}[\mathbf{HAF}_{w^{(\ell)}}]$

We will show that for $f(w) = w^{(\ell)}$, for any nonnegative integer ℓ:

$$\mathbb{E}[\mathrm{HAF}_{w^{(\ell)}}] = \frac{\theta}{n(\ell+1)} \cdot (n-1)^{(\ell+1)}\,. \tag{S13}$$

For this proof only, abbreviate $W = W_{k,1}$. Note that

$$W \cdot W^{(\ell)} = (W + \ell - \ell)W^{(\ell)} = (W+\ell)W^{(\ell)} - \ell\, W^{(\ell)} = W^{(\ell+1)} - \ell\, W^{(\ell)}. \tag{S14}$$

Using this and Eq. (S6) gives

$$\begin{aligned}\mathbb{E}[\mathrm{HAF}_{w^{(\ell)}}] &= \frac{\theta}{n}\sum_{k=2}^{n}\frac{1}{k-1}\mathbb{E}[W \cdot W^{(\ell)}] \\ &= \frac{\theta}{n}\sum_{k=2}^{n}\frac{1}{k-1}\mathbb{E}[W^{(\ell+1)} - \ell\, W^{(\ell)}] \\ &= \frac{\theta}{n}\sum_{k=2}^{n}\frac{1}{k-1}\left((\ell+1)!\,\frac{n^{(\ell+1)}}{k^{(\ell+1)}} - \ell \cdot \ell! \cdot \frac{n^{(\ell)}}{k^{(\ell)}}\right) &\text{(S15)} \\ &= \frac{\theta \cdot \ell! \cdot n^{(\ell)}}{n}\sum_{k=2}^{n}\left(\frac{(\ell+1)(n+\ell)}{(k-1)^{(\ell+2)}} - \frac{\ell}{(k-1)^{(\ell+1)}}\right). &\text{(S16)}\end{aligned}$$

We will split this sum into two telescoping sums. One may show that

$$\frac{1}{k^{(\ell)}} = \frac{1}{\ell - 1}\left(\frac{k+\ell-1}{k^{(\ell)}} - \frac{k+\ell}{(k+1)^{(\ell)}}\right). \tag{S17}$$

Using this with shifted k's and ℓ's, part of the sum in Eq. (S16) can be written

$$\sum_{k=2}^{n} \frac{1}{(k-1)^{(\ell+2)}} = \sum_{k=2}^{n} \frac{1}{\ell+1}\left(\frac{k+\ell}{(k-1)^{(\ell+2)}} - \frac{k+\ell+1}{k^{(\ell+2)}}\right),$$

which is a telescoping sum that evaluates to

$$= \frac{1}{\ell+1}\left(\frac{2+\ell}{1^{(\ell+2)}} - \frac{n+\ell+1}{n^{(\ell+2)}}\right).$$

Note that $1^{(\ell+2)} = (\ell+2)!$, simplifying this to

$$= \frac{1}{\ell+1}\left(\frac{1}{(\ell+1)!} - \frac{n+\ell+1}{n^{(\ell+2)}}\right). \tag{S18}$$

Similarly,

$$\sum_{k=2}^{n} \frac{1}{(k-1)^{(\ell+1)}} = \frac{1}{\ell}\left(\frac{1}{\ell!} - \frac{n+\ell}{n^{(\ell+1)}}\right). \tag{S19}$$

Plugging Eqs. (S18),(S19) into Eq. (S16), we obtain:

$$\begin{aligned}
\mathbb{E}[\mathrm{HAF}_{w^{(\ell)}}] &= \frac{\theta \cdot \ell! \cdot n^{(\ell)}}{n}\left(\frac{(\ell+1)(n+\ell)}{\ell+1}\left(\frac{1}{(\ell+1)!} - \frac{n+\ell+1}{n^{(\ell+2)}}\right) - \frac{\ell}{\ell}\left(\frac{1}{\ell!} - \frac{n+\ell}{n^{(\ell+1)}}\right)\right) \\
&= \frac{\theta \cdot n^{(\ell)}}{n}\left((n+\ell)\left(\frac{1}{\ell+1} - \frac{1}{n^{(\ell+1)}}\right) - \left(1 - \frac{n+\ell}{n^{(\ell+1)}}\right)\right) \\
&= \frac{\theta \cdot n^{(\ell)}}{n}\left(\frac{n+\ell}{\ell+1} - 1\right) = \frac{\theta \cdot n^{(\ell)}}{n} \cdot \frac{n-1}{\ell+1} = \frac{\theta \cdot (n-1)^{(\ell+1)}}{n(\ell+1)}.
\end{aligned}$$

This proves Eq. (S13).

Step 3: The final form $\mathbb{E}[\ell$-HAF]

Powers w^ℓ and rising factorials $w^{(\ell)}$ may be expressed as linear combinations of each other via Stirling numbers. Let $c(\ell, q)$ denote the Unsigned Stirling Number of the First Kind, and $S(\ell, q)$ denote the Stirling Number of the Second Kind. Then [4, p. 264]

$$\text{(a) } w^\ell = \sum_{q=0}^{\ell} (-1)^{\ell-q} S(\ell, q) w^{(q)}, \qquad \text{(b) } w^{(\ell)} = \sum_{q=0}^{\ell} c(\ell, q)\, w^q. \tag{S20}$$

The expected value of $(W_{k,i})^\ell$ is then:

$$\mathbb{E}[(W_{k,i})^\ell] = \sum_{q=0}^{\ell} (-1)^{\ell-q} S(\ell, q)\, \mathbb{E}[(W_{k,i})^{(q)}] = \sum_{q=0}^{\ell} (-1)^{\ell-q} S(\ell, q)\, q! \cdot \frac{n^{(q)}}{k^{(q)}}. \tag{S21}$$

We evaluate $\mathbb{E}[\ell\text{-HAF}] = \mathbb{E}[\mathrm{HAF}_{w^\ell}]$ as a linear combination of terms $\mathbb{E}[\mathrm{HAF}_{w^{(q)}}]$, using the same coefficients that express w^ℓ as a linear combination of terms of $w^{(q)}$ (Eq. (S20a)):

$$\mathbb{E}[\ell\text{-HAF}] = \sum_{q=0}^{\ell} (-1)^{\ell-q} S(\ell, q) \mathbb{E}[\mathrm{HAF}_{w^{(q)}}] = \frac{\theta}{n} \sum_{q=0}^{\ell} (-1)^{\ell-q} S(\ell, q) \cdot \frac{(n-1)^{(q+1)}}{q+1}. \tag{S22}$$

For $\ell = 0$:

$$E[0\text{-HAF}] = \frac{\theta}{n}(-1)^{0-0} S(0,0) \frac{(n-1)^{(1)}}{1} = \frac{\theta}{n} 1 \cdot 1 \cdot (n-1) = \frac{\theta(n-1)}{n} = \theta\left(1 - \frac{1}{n}\right). \tag{S23}$$

For $\ell = 1$:

$$E[\text{1-HAF}] = \frac{\theta}{n}\left((-1)^{1-0}S(1,0)\frac{(n-1)^{(1)}}{1} + (-1)^{1-1}S(1,1)\frac{(n-1)^{(2)}}{2}\right)$$
$$= \frac{\theta}{n}\left(-0\cdot(n-1) + 1\cdot\frac{(n-1)(n)}{2}\right) = \theta\cdot\frac{n-1}{2}.$$

For $\ell = 2$:

$$E[\text{2-HAF}] = \frac{\theta}{n}\Bigg((-1)^{2-0}S(2,0)\frac{(n-1)^{(1)}}{1} + (-1)^{2-1}S(2,1)\frac{(n-1)^{(2)}}{2}$$
$$+ (-1)^{2-2}S(2,2)\frac{(n-1)^{(3)}}{3}\Bigg)$$
$$= \frac{\theta}{n}\left(0\cdot\frac{n-1}{1} - 1\cdot\frac{(n-1)n}{2} + 1\cdot\frac{(n-1)n(n+1)}{3}\right) = \frac{\theta(n-1)(2n-1)}{6}.$$

It is straightforward to compute this for any ℓ in the same fashion.

$$E[\text{3-HAF}] = \frac{\theta\cdot n(n-1)^2}{4} \qquad E[\text{4-HAF}] = \frac{\theta\cdot(n-1)(2n-1)(3n^2-3n-1)}{30} \qquad \cdots$$

Equivalence of Eq. (3) and Eq. (7) for $\mathbb{E}[\ell\text{-HAF}]$ in a constant-sized population

Eq. (3) has simple derivation, but a variable number of terms, n, that grows as n grows. Eq. (7) leads to an evaluation (S22) that is a polynomial in n of degree ℓ. Both represent $\mathbb{E}[\ell\text{-HAF}]$ in a constant-sized population; we now show that they are equal as a consequence of the following lemma:

Lemma 1 *In a constant-sized population, for any polynomial $f(w)$, we have*

$$\mathbb{E}[HAF_f] = \frac{\theta}{n}\sum_{w=1}^{n-1} f(w).$$

Applying this to $f(w) = w^\ell$ gives equivalence of the two formulas (3) and (S22) for $\mathbb{E}[\ell\text{-HAF}]$.

Proof of lemma. Both sides are linear in f, so it suffices to prove it on any basis of polynomials. We use the basis of rising factorials, $f(w) = w^{(\ell)}$ for $\ell \geq 0$. Eq. (S13) evaluates $\mathbb{E}[\text{HAF}_f]$ on this basis. We now evaluate $\sum_{w=1}^{n-1} w^{(\ell)}$. Note that

$$w^{(\ell+1)} - (w-1)^{(\ell+1)} = w^{(\ell)}\cdot(w+\ell-(w-1)) = (\ell+1)\cdot w^{(\ell)},$$

so

$$w^{(\ell)} = \frac{w^{(\ell+1)} - (w-1)^{(\ell+1)}}{\ell+1}.$$

This leads to a telescoping sum:

$$\sum_{w=1}^{n-1} w^{(\ell)} = \frac{1}{\ell+1}\sum_{w=1}^{n-1}\left(w^{(\ell+1)} - (w-1)^{(\ell+1)}\right) = \frac{(n-1)^{(\ell+1)} - 0^{(\ell+1)}}{\ell+1} = \frac{(n-1)^{(\ell+1)}}{\ell+1}.$$

Multiplying this sum by θ/n gives the same value as (S13) gives for $\mathbb{E}[\text{HAF}_{w^{(\ell)}}]$. Thus, the lemma holds on the basis $f(w) = w^{(\ell)}$ for $\ell \geq 0$. By linearity, it holds on all polynomials $f(w)$. ■

Deriving $\mathbb{E}[\ell\text{-HAF}]$ in an exponentially growing population

Let the population size at time t in the past be given by $N(t) = N_0\, e^{-rt}$, where N_0 is the current population size. $M_{k,i}$ depends on T_k, which in turn depends on the size of the population in epoch k. Slatkin and Hudson (1991) [5, p. 559] derived an iterative formula to generate a sequence of random times T_k ($k = n, n-1, \ldots, 2$).

The actual formula Hudson implemented in the simulator *ms* [6] (precursor to *msms* [7]) is slightly different; in our notation, it is as follows. Let $\alpha = 2\,N_0\,r$ be a scaled growth rate. Generate random values $U_n, \ldots, U_2$ that are uniformly distributed in $(0,1)$, and iteratively compute $T_n, \ldots, T_2$ via

$$T_k = \frac{1}{r}\ln\left[1 - \frac{\alpha}{k(k-1)}\,e^{-r\,\tau_k}\,\ln(U_k)\right], \qquad \text{where} \qquad \tau_k = \sum_{i=k+1}^{n} T_k\,. \tag{S24}$$

Then generate a random value for each $M_{k,i}$ using a Poisson distribution with mean $\mu\,T_k$ ($k = 2, \ldots, n$ and $i = 1, \ldots, k$). This does not lead to a closed form expression for the expected HAF scores, but we may nonetheless use it to estimate the expected HAF scores computationally, as illustrated in S2 Fig.

The first method, *cumulative time*, is to generate random values of T_k's and $M_{k,i}$'s as above, and plug them into (S10), along with exact values of $\mathbb{E}[W_{k,i} \cdot f(W_{k,i})]$ computed using (S6) or (S21).

The second method is *conditional expectation.* For a given k, we may compute the expected value of (S24) given the condition that $T_{k+1}, \ldots, T_n$ are known. This gives estimates $T_k = t_k$ (computed in order $k = n, n-1, \ldots, 2$) as follows:

$$\begin{aligned} t_k &= \mathbb{E}\left[T_k \mid T_{k+1} = t_{k+1}, \ldots, T_n = t_n\right] \\ &= \frac{1}{r}\int_0^1 \ln\left(1 - \frac{\alpha}{k(k-1)}\,e^{-r\cdot\tau_k}\,\ln(u)\right)du \\ &= \frac{1}{r}\exp\left(\frac{k(k-1)}{\alpha}e^{r\cdot\tau_k}\right)E_1\left(\frac{k(k-1)}{\alpha}e^{r\cdot\tau_k}\right), \end{aligned} \tag{S25}$$

where $\tau_k = t_{k+1} + \cdots + t_n$ (with $\tau_n = 0$) and $E_1(x)$ is the exponential integral $E_1(x) = \int_1^\infty \frac{\exp(-x\,t)}{t}\,dt$.

In practice, Eq. (S25) is used to generate scaled times,

$$r \cdot t_k = \mathbb{E}[r \cdot T_k | r \cdot T_{k+1} = r \cdot t_{k+1}, \ldots, r \cdot T_n = r \cdot t_n].$$

In terms of these times, $\mathbb{E}[M_{k,i}] = \mu\mathbb{E}[T_k] \approx \mu\,t_k$ (which is approximate since the formula for t_k uses conditional expectation while this equation does not). We then estimate $\mathbb{E}[\mathrm{HAF}_f]$ via (S11):

$$\mathbb{E}[\mathrm{HAF}_f] \approx \frac{1}{n}\sum_{k=2}^{n} k \cdot \mu\,t_k \cdot \mathbb{E}[W_{k,1} \cdot f(W_{k,1})] = \frac{\mu}{r}\sum_{k=2}^{n}(r \cdot t_k) \cdot \frac{k}{n} \cdot \mathbb{E}[W_{k,1} \cdot f(W_{k,1})]. \tag{S26}$$

For $f(w) = w^{(\ell)}$, similarly to (S15), this gives an estimate

$$\begin{aligned} \mathbb{E}[\mathrm{HAF}_{w^{(\ell)}}] &\approx \frac{\mu}{r}\sum_{k=2}^{n}(r \cdot t_k) \cdot \frac{k}{n}\left((\ell+1)!\,\frac{n^{(\ell+1)}}{k^{(\ell+1)}} - \ell \cdot \ell! \cdot \frac{n^{(\ell)}}{k^{(\ell)}}\right) \\ &= \frac{\mu}{r}\sum_{k=2}^{n}(r \cdot t_k) \cdot \left((\ell+1)!\,\frac{(n+1)^{(\ell)}}{(k+1)^{(\ell)}} - \ell \cdot \ell! \cdot \frac{(n+1)^{(\ell-1)}}{(k+1)^{(\ell-1)}}\right), \end{aligned} \tag{S27}$$

and for $f(w) = w^\ell$, we use (S20) to obtain

$$\mathbb{E}[\ell\text{-HAF}] \approx \frac{\mu}{r}\sum_{k=2}^{n}(r \cdot t_k)\sum_{q=0}^{\ell}(-1)^{\ell-q}S(\ell,q)\left((q+1)!\,\frac{(n+1)^{(q)}}{(k+1)^{(q)}} - q \cdot q! \cdot \frac{(n+1)^{(q-1)}}{(k+1)^{(q-1)}}\right). \tag{S28}$$

In terms of $\alpha = 2\,N_0\,r$ and $\theta = 2\,N_0\,\mu$, the coefficient μ/r may be replaced by θ/α.

We used Maple (maplesoft.com) to generate the sequence of scaled times $r \cdot t_k$ by iterating Eq. (S25). Maple supports arbitrary precision numerical computations, which is needed due to complications with double precision arithmetic. Let $x = \frac{k(k-1)}{\alpha}\exp(r \cdot \tau_k)$. Small rounding errors in x due to limited precision are amplified in e^x, which may lead to slightly different results when iterating the recursion using double precision arithmetic vs. Maple's arbitrary precision arithmetic. Additionally, as x increases, e^x grows rapidly and leads to an overflow in double precision arithmetic at approximately $x \geq 709.7827$, while $E_1(x)$ decays rapidly and leads to an underflow at approximately $x \geq 701.8334$, even though $e^x E_1(x)$ is usually representable in double precision. For example, $E_1(702) \approx 1.9 \cdot 10^{-308}$ underflows in double precision arithmetic, but $e^{702}E_1(702) \approx 0.0014$ is representable. The largest value of x used in Eq. (S25) is $x = n(n-1)/\alpha$ in iteration $k = n$, leading to an underflow in double precision if $n(n-1)/\alpha > 701.8$.

Number of mutations in a genealogy

Let $f(w) = 1/w$ for $w > 0$ and $f(0) = 0$. Note that for an individual HAF vector $\mathbf{c}$, some entries may be 0, but in a genealogy, all $w_{k,i} \neq 0$ since every branch of the observed tree has at least one haplotype as its descendant. The sum (S9) of $\mathrm{HAF}_f(\mathbf{c})$ over all haplotypes in a genealogy becomes

$$\sum_{v=1}^{n} \mathrm{HAF}_f(\mathbf{c}(v)) = \sum_{k=2}^{n} \sum_{i=1}^{k} m_{k,i}\,, \tag{S29}$$

and thus (S11) becomes

$$\mathbb{E}[\mathrm{HAF}_f] = \frac{1}{n} \sum_{k=2}^{n} k \cdot \mathbb{E}[M_{k,1}]\,. \tag{S30}$$

In a constant-sized population, Eqs. (S3) and (S29) give that the expected number of mutations in a genealogy is

$$\mathbb{E}[\mathrm{HAF}_f] = \frac{1}{n} \sum_{k=2}^{n} k \cdot \frac{\theta}{k(k-1)} = \frac{\theta}{n} \sum_{k=2}^{n} \frac{1}{k-1} = \frac{\theta}{n} H(n-1)\,, \tag{S31}$$

where $H(n) = \sum_{k=1}^{n} \frac{1}{k}$ is the Harmonic number.

Under exponential growth, we instead estimate times t_k or scaled times $r \cdot t_k$ via (11). Then we estimate the expected number of mutations by

$$\mathbb{E}[\mathrm{HAF}_f] \approx \frac{\mu}{n} \sum_{k=2}^{n} k \cdot t_k = \frac{\mu/r}{n} \sum_{k=2}^{n} k \cdot (r \cdot t_k) = \frac{\theta/\alpha}{n} \sum_{k=2}^{n} k \cdot (r \cdot t_k)\,. \tag{S32}$$

Computing the mean HAF_f score from a SNP matrix

For a sample of n individuals whose genealogy has q segregating sites, the SNP matrix A consists of n rows (each row representing one individual) and q columns, with entry 0 denoting ancestral alleles and 1 denoting derived alleles.

The vth row of A is a haplotype vector as depicted as $\mathbf{h}$ in Fig 1.

Let $\mathbf{w}^{\text{all}}$ denote the sum of the rows of A. The frequency of the jth allele is w_j^{all}, the sum of all entries in the jth column of A.

In $\sum_{v=1}^{n} \mathrm{HAF}_f(\mathbf{c}(v))$, the jth mutation contributes $f(w_j)$ for each of the w_j haplotypes that have that mutation ($A_{vj} = 1$) and contributes 0 on each of the $n - w_j$ others. Thus, the average of HAF_f over the genealogy is

$$\frac{1}{n} \sum_{v=1}^{n} \mathrm{HAF}_f(\mathbf{c}(v)) = \frac{1}{n} \sum_{j=1}^{q} f(w_j^{\text{all}}) \cdot w_j^{\text{all}}\,. \tag{S33}$$

We use this to compute empirical averages of HAF_f in simulations (*ms* [6] and *msms* [7] output a SNP matrix), as well as in further theoretical developments. In particular, given a haplotype vector $\mathbf{h}$ and the corresponding HAF vector $\mathbf{c}$, we have

$$\text{1-HAF}(\mathbf{c}) = \mathbf{w}^{\text{all}} \cdot \mathbf{h}\,. \tag{S34}$$

HAF score dynamics and peak values

Consider n haplotypes randomly sampled from a Wright-Fischer (WF) fixed-size population of N haploid individuals under a hard sweep with selection coefficient s. Let ν denote the fraction of individuals that carry the favored allele. We assume the sample has at least one carrier and at least one non-carrier, so that $\nu \in \{\frac{1}{n}, \frac{2}{n}, \ldots, \frac{n-1}{n}\}$; at fixation ($\nu = 1$), there are no non-carriers remaining. We assume strong selection ($Ns \gg 1$) and no recombination in the region being sampled. We measure time in generations going backwards. See S13 Fig. The current time is time 0; let T^{car} denote the time when all sampled carriers coalesce to their most recent common ancestor (denoted by MRCA^{car}). Let T^{all} denote the time when all

sampled individuals coalesce to a common ancestor (denoted by MRCA^{all}). Let $T(k)$ denote time to MRCA of k randomly chosen haplotypes in a population of size N. From Nordborg [8], we have

$$\mathbb{E}[T(k)] = 2N\left(1 - \frac{1}{k}\right). \tag{S35}$$

At time T^{car}, we have exactly one ancestor of the favored allele carrier, and assume we have m ancestors of non-carriers. In a hard sweep scenario, the favored mutation arises at the same time as the onset of selection; therefore, the time between T^{car} and T^{all} is governed by the neutral WF model with population N, and is well approximated by neutral coalescent theory. Applying Eq. (S35) to the remaining sample of $m+1$ individuals at time T^{car} gives the expected time to coalesce:

$$\mathbb{E}[T^{\text{all}} - T^{\text{car}}] = \mathbb{E}[T(m+1)] = 2N\left(1 - \frac{1}{m+1}\right). \tag{S36}$$

Let A denote the SNP matrix of a sample of n individuals, with νn carriers of the favored allele (S14 Fig). We order the columns so that all mutations are ordered chronologically from left to right. Similarly, the rows are ordered so that the first νn rows correspond to carrier haplotypes.

The ith haplotype is represented by the ith row of A (denoted by row vector $\mathbf{h_i}$) and has 1-HAF score denoted by 1-HAF$_i$. From Eq. (S34), we have

$$\text{1-HAF}_i = \mathbf{w}^{\text{all}} \cdot \mathbf{h}_i\ .$$

We partition the matrix into three submatrices: A_1 consists of the rows corresponding to carriers and columns corresponding to mutations occurring prior to the onset of selection (times between T^{car} and T^{all}). A_2 is the submatrix from all non-carrier rows and mutations prior to onset of selection. Finally, A_3 is the submatrix of all columns after the onset of selection (times between 0 and T^{car}). Define $\mathbf{w}^{\text{car}}$ as the sum of all carrier haplotypes; similarly, let $\mathbf{w}^{\text{non}}$ be the sum of all non-carrier haplotypes. Thus,

$$\mathbf{w}^{\text{all}} = \mathbf{w}^{\text{car}} + \mathbf{w}^{\text{non}}.$$

Also, we can partition the equation for computing 1-HAF$_i$ by rows as

$$\text{1-HAF}_i = \mathbf{w}^{\text{car}} \cdot \mathbf{h}_i + \mathbf{w}^{\text{non}} \cdot \mathbf{h}_i\ .$$

We partition a haplotype vector $\mathbf{h}$ in the matrix by separating SNPs that occurred before (subvector $\mathbf{h}^b$) and after (subvector $\mathbf{h}^a$) the onset of mutation. A similar partitioning works for $\mathbf{w}^{\text{all}}, \mathbf{w}^{\text{car}}$, and $\mathbf{w}^{\text{non}}$. Thus, for a random haplotype $\mathbf{h}$,

$$\text{1-HAF} = (\mathbf{w}^{\text{car}})^b \cdot \mathbf{h}^b + (\mathbf{w}^{\text{non}})^b \cdot \mathbf{h}^b + (\mathbf{w}^{\text{all}})^a \cdot \mathbf{h}^a\ .$$

The expected 1-HAF score of a carrier (or non-carrier) haplotype $\mathbf{h}$ can similarly be decomposed as

$$\mathbb{E}[\text{1-HAF}] = \mathbb{E}[(\mathbf{w}^{\text{car}})^b \cdot \mathbf{h}^b] + \mathbb{E}[(\mathbf{w}^{\text{non}})^b \cdot \mathbf{h}^b] + \mathbb{E}[(\mathbf{w}^{\text{all}})^a \cdot \mathbf{h}^a]\ . \tag{S37}$$

To compute the expected 1-HAF score, we bound each of these constituent terms.

Lemma 2 *Consider a sample of n individuals under a hard sweep with νn carriers ($\frac{1}{n} \le \nu \le \frac{n-1}{n}$). Let m be the number of non-carrier haplotypes remaining at time T^{car}. Then for a random carrier haplotype $\mathbf{h}$, we have*

$$\mathbb{E}[(\mathbf{w}^{car})^b \cdot \mathbf{h}^b] = 2N\mu\nu n\left(1 - \frac{1}{m+1}\right), \tag{S38}$$

$$\mathbb{E}[(\mathbf{w}^{non})^b \cdot \mathbf{h}^b] = 2N\mu(1-\nu)n\left(\frac{1}{2} - \frac{1}{m+1}\right), \tag{S39}$$

while for a random non-carrier haplotype $\mathbf{h}$, these become

$$\mathbb{E}[(\mathbf{w}^{car})^b \cdot \mathbf{h}^b] = 2N\mu\nu n\left(\frac{1}{2} - \frac{1}{m+1}\right), \tag{S40}$$

$$\mathbb{E}[(\mathbf{w}^{non})^b \cdot \mathbf{h}^b] = 2N\mu(1-\nu)n\left(\frac{1}{2} - \frac{m-1}{2(m+1)(1-\nu)n}\right). \tag{S41}$$

Proof of Eq. (S38). Mutations that happen on the lineage from MRCA^{all} to MRCA^{car} are shared by all carriers, so all rows of matrix A_1 are identical. Thus, for all carrier haplotypes $\mathbf{h}_i, \mathbf{h}_j$, we have $\mathbf{h}_i^b = \mathbf{h}_j^b$. On restricting $\mathbf{w}^{\text{car}} = \sum_{i=1}^{\nu n} \mathbf{h}_i$ to the mutations from before the onset of selection, we obtain

$$\mathbb{E}[(\mathbf{w}^{\text{car}})^b \cdot \mathbf{h}^b] = \sum_{i=1}^{\nu n} \mathbb{E}[\mathbf{h}_i^b \cdot \mathbf{h}^b] = \nu\, n\, \mathbb{E}[\mathbf{h}^b \cdot \mathbf{h}^b]. \tag{S42}$$

The term $\mathbb{E}[\mathbf{h}^b \cdot \mathbf{h}^b]$ is the expected number of ones in $\mathbf{h}^b$, which is the expected number of mutations on the lineage from MRCA^{all} to MRCA^{car}. Therefore, $\mathbb{E}[\mathbf{h}^b \cdot \mathbf{h}^b] = \mu\mathbb{E}[T^{\text{all}} - T^{\text{car}}]$. By Eq. (S36),

$$\mathbb{E}[\mathbf{h}^b \cdot \mathbf{h}^b] = \mu\mathbb{E}[T^{\text{all}} - T^{\text{car}}] = 2N\mu\left(1 - \frac{1}{m+1}\right). \tag{S43}$$

Plugging this into Eq. (S42) gives Eq. (S38). ■

Proof of Eq. (S39). On restricting $\mathbf{w}^{\text{non}} = \sum_{i=\nu n+1}^{n} \mathbf{h}_i$ to the mutations from before the onset of selection, we obtain

$$\mathbb{E}[(\mathbf{w}^{\text{non}})^b \cdot \mathbf{h}^b] = \sum_{i=\nu n+1}^{n} \mathbb{E}[\mathbf{h}_i^b \cdot \mathbf{h}^b]. \tag{S44}$$

Going back from time T^{car} to T^{all}, we observe the coalescence of the ancestors of carrier haplotype $\mathbf{h}$ and non-carrier haplotype $\mathbf{h}_i$ at a time T', where $T^{\text{car}} \le T' \le T^{\text{all}}$. The term $\mathbb{E}[\mathbf{h}_i^b \cdot \mathbf{h}^b]$ is the expected number of ones in common between these two haplotypes among the SNPs occurring before the onset of selection. This equals the expected number of mutations in the lineage from T' to T^{all}:

$$\mathbb{E}[\mathbf{h}_i^b \cdot \mathbf{h}^b] = \mu\mathbb{E}[T^{\text{all}} - T'] = \mu\mathbb{E}\big[(T^{\text{all}} - T^{\text{car}}) - (T' - T^{\text{car}})\big] = \mu\big(\mathbb{E}[T(m+1)] - \mathbb{E}[T(2)]\big). \tag{S45}$$

Note that $T' - T^{\text{car}}$ is the time for a sample of 2 individuals (ancestors of $\mathbf{h}$ and $\mathbf{h}_i$ at time T^{car}) to coalesce. We evaluate Eq. (S45) using Eq. (S35):

$$\mathbb{E}[\mathbf{h}_i^b \cdot \mathbf{h}^b] = 2N\mu\left(\frac{1}{2} - \frac{1}{m+1}\right). \tag{S46}$$

Plug this into Eq. (S44). All $n - \nu n = (1-\nu)n$ terms are the same, so it simplifies to Eq. (S39). ■

Proof of Eq. (S40). We have

$$\mathbb{E}[(\mathbf{w}^{\text{car}})^b \cdot \mathbf{h}^b] = \sum_{i=1}^{\nu n} \mathbb{E}[\mathbf{h}_i^b \cdot \mathbf{h}^b]. \tag{S47}$$

Here, $\mathbf{h}$ is a non-carrier while $\mathbf{h}_i$ is a carrier, so they are distinct and they coalesce prior to the onset of selection. Thus, Eq. (S46) applies here. All νn terms of the sum in Eq. (S47) evaluate to Eq. (S46), so it simplifies to Eq. (S40). ■

Proof of Eq. (S41). We have

$$\mathbb{E}[(\mathbf{w}^{\text{non}})^b \cdot \mathbf{h}^b] = \sum_{i=\nu n+1}^{n} \mathbb{E}[\mathbf{h}_i^b \cdot \mathbf{h}^b]. \tag{S48}$$

The term $\mathbb{E}[\mathbf{h}_i^b \cdot \mathbf{h}^b]$ is the expected number of ones in common in haplotypes $\mathbf{h}$ and $\mathbf{h}_i$ among the SNPs occurring before the onset of selection.

We apply Lemma 3 (upcoming) to show that two random non-carrier haplotypes $\mathbf{h}$ and $\mathbf{h_i}$ have the same ancestor at time T^{car} with probability

$$p = 1 - \left(1 + \frac{1}{(1-\nu)n}\right)\left(1 - \frac{2}{m+1}\right)$$

or different ancestors with probability $1-p$. Thus, with probability p, we have $\mathbf{h}_i^b = \mathbf{h}^b$, and with probability $1-p$, we have $\mathbf{h}_i^b \neq \mathbf{h}^b$. Therefore,

$$\mathbb{E}[\mathbf{h}_i^b \cdot \mathbf{h}^b] = p \cdot \mathbb{E}\left[\mathbf{h}_i^b \cdot \mathbf{h}^b \mid \mathbf{h}_i^b = \mathbf{h}^b\right] + (1-p)\mathbb{E}\left[\mathbf{h}_i^b \cdot \mathbf{h}^b \mid \mathbf{h}_i^b \neq \mathbf{h}^b\right]. \tag{S49}$$

Term $\mathbb{E}\left[\mathbf{h}_i^b \cdot \mathbf{h}^b \mid \mathbf{h}_i^b = \mathbf{h}^b\right]$ is the expected number of ones in $\mathbf{h}^b$, which is $\mu\mathbb{E}[T^{\,\mathrm{all}} - T^{\,\mathrm{car}}]$. Term $\mathbb{E}\left[\mathbf{h}_i^b \cdot \mathbf{h}^b \mid \mathbf{h}_i^b \neq \mathbf{h}^b\right]$ is the expected number of ones in common between $\mathbf{h}_i^b$ and $\mathbf{h}^b$, which is $\mu\mathbb{E}[T^{all} - T']$. These expected times were evaluated in Eqs. (S43) and (S45). Then

$$\begin{aligned}
\mathbb{E}[\mathbf{h}_i^b \cdot \mathbf{h}^b] &= p\mu\mathbb{E}[T^{\,\mathrm{all}} - T^{\,\mathrm{car}}] + (1-p)\mu\mathbb{E}[T^{\,\mathrm{all}} - T'] \\
&= \left(1 - \left(1 + \frac{1}{(1-\nu)n}\right)\left(1 - \frac{2}{m+1}\right)\right) 2N\mu \left(1 - \frac{1}{m+1}\right) \\
&\quad + \left(1 + \frac{1}{(1-\nu)n}\right)\left(1 - \frac{2}{m+1}\right) 2N\mu \left(\frac{1}{2} - \frac{1}{m+1}\right) \\
&= 2N\mu \left(\frac{1}{2} - \frac{m-1}{2(m+1)(1-\nu)n}\right) \qquad \text{(S50)}
\end{aligned}$$

Eq. (S48) is a sum of $(1-\nu)n$ terms, each of form (S50), so Eq. (S48) evaluates to Eq. (S41). ■

In the preceding proof, the probability p that two non-carriers share the same ancestor was computed using the following lemma, applied to $n_0 = (1-\nu)n$ non-carriers in the sample and m ancestors of non-carriers at time $T^{\,\mathrm{car}}$.

Lemma 3 *Consider a subsample of n_0 individuals, which coalesce to m ancestors at some time T, while the rest of the sample coalesces to one or more ancestors distinct from these m. Draw two individuals (with replacement) from the subsample. The probability that they have the same ancestor at time T is*

$$\frac{2n_0 - m + 1}{n_0(m+1)} = 1 - \left(1 + \frac{1}{n_0}\right)\left(1 - \frac{2}{m+1}\right). \qquad \text{(S51)}$$

Proof. Throughout this proof, pairs of individuals (x, y) are drawn from the subsample of size n_0 *with replacement*. There are ${n_0}^2$ such pairs.

Consider the m lineages at time T. The number of pairs of individuals deriving from the ith lineage is $(w_{m,i})^2$. Sum over all lineages to obtain the total number of pairs of individuals with the same time T ancestor: $\sum_{i=1}^{m} (w_{m,i})^2$. The expected number of pairs with the same ancestor is

$$\begin{aligned}
\sum_{i=1}^{m} \sum_{w=1}^{n_0-m+1} \Pr(W_{m,i} = w) w^2 = \sum_{i=1}^{m} \sum_{w=1}^{n_0-m+1} \frac{\binom{n_0-w-1}{m-2}}{\binom{n_0-1}{m-1}} w^2 = m \sum_{w=1}^{n_0-m+1} \frac{\binom{n_0-w-1}{m-2}}{\binom{n_0-1}{m-1}} w^2 \\
= \frac{n_0(2n_0 - m + 1)}{m+1}.
\end{aligned}$$

Note that $\Pr(W_{m,i} = w)$ is given by Eq. (S4), and holds upon restricting the full genealogy to the subsample. Divide this expected value by ${n_0}^2$ pairs and simplify to obtain Eq. (S51). ■

Lemma 4 *Consider a sample of n individuals under a hard sweep. For a random haplotype* $\mathbf{h}$ *(carrier or non-carrier), we have*

$$0 \le \mathbb{E}[(\mathbf{w}^{\,all})^a \cdot \mathbf{h}^a] \le \mu n \mathbb{E}[T^{\,car}]\,. \qquad \text{(S52)}$$

Proof. We have

$$\mathbb{E}[(\mathbf{w}^{\,\mathrm{all}})^a \cdot \mathbf{h}^a] = \sum_{i=1}^{n} \mathbb{E}[\mathbf{h}_i^a \cdot \mathbf{h}^a]\,. \qquad \text{(S53)}$$

The dimension of vectors $\mathbf{h}_i^a$ and $\mathbf{h}$ is the number of mutations in $[0, T^{\,\mathrm{car}}]$. The expected value of this dimension is $\mu\mathbf{E}[T^{\,\mathrm{car}}]$. The dot product of two binary vectors is at least 0, and is bounded above by their dimension, so

$$0 \le \mathbb{E}[\mathbf{h}_i^a \cdot \mathbf{h}^a] \le \ \mu\mathbb{E}[T^{\,\mathrm{car}}]\,. \qquad \text{(S54)}$$

Plugging Eq. (S54) into Eq. (S53) gives Eq. (S52). ■

Lemma 5 *Consider a population of size N with selection coefficient s, under a hard sweep with strong selection ($Ns \gg 1$). Then*

$$0 \leq \mathbb{E}[(\mathbf{w}^{\,all})^a \cdot \mathbf{h}^a] \leq \theta n \frac{\ln(2Ns)}{2Ns}\ . \tag{S55}$$

Proof. Campbell [9] shows that under strong selection, the fixation time is approximately $\frac{1}{s}\ln(2Ns)$. As $T^{\,\mathrm{car}}$ is the time for the favored allele to reach frequency νn, it is dominated by the fixation time. Therefore,

$$\mathbb{E}[T^{\,\mathrm{car}}] \leq \frac{1}{s}\ln(2Ns)\,. \tag{S56}$$

Plug Eq. (S56) and the scaled mutation rate $\theta = 2N\mu$ into Lemma 4 to obtain Eq. (S55). ■

Lemma 6 *Consider a sample of n individuals under a hard sweep with νn carriers ($\frac{1}{n} \leq \nu \leq \frac{n-1}{n}$). In the time $[0, T^{\,car}]$, let the number of coalescent events among the $(1-\nu)n$ non-carrier haplotypes be $\varepsilon_\nu(1-\nu)n$. Then*

$$0 \leq \varepsilon_\nu \leq \varepsilon_\nu^{\max} \quad \textit{where} \quad \varepsilon_\nu^{\max} = \frac{1}{1 + \frac{2Ns}{n\ln(2Ns)}}\,. \tag{S57}$$

Proof. Since we have $(1-\nu)n$ non-carrier haplotypes in our sample, the expected number of non-carriers in the whole population is $(1-\nu)N$. Let $N_0^{\mathrm{non}} = (1-\nu)N$, and let N_u^{non} be the expected number of non-carriers in the whole population when the uth coalescent event occurs among the non-carrier samples. As we go backwards in time (increasing u), the expected number of non-carriers increases:

$$N_u^{\mathrm{non}} \geq N_{u-1}^{\mathrm{non}} \geq N_0^{\mathrm{non}} = (1-\nu)N \qquad \text{for } 1 \leq u \leq \varepsilon_\nu(1-\nu)n. \tag{S58}$$

Additionally, the chance of coalescing decreases and the expected coalescent time increases. For a fixed size population of size N, the expected time for one coalescent event in a sample of size n is $N/\binom{n}{2}$ (see [8]). Hence, the expected time for the uth coalescent event is at least

$$N_{u-1}\Big/\binom{(1-\nu)n-u+1}{2}.$$

On the other hand, the sum of all coalescent times during the selective sweep is at most $T^{\,\mathrm{car}}$. Therefore,

$$\begin{aligned}
\mathbb{E}[T^{\,\mathrm{car}}] &\geq \sum_{u=1}^{\varepsilon_\nu(1-\nu)n} \frac{2N_{u-1}}{((1-\nu)n-u+1)((1-\nu)n-u)} \\
&\geq \sum_{u=1}^{\varepsilon_\nu(1-\nu)n} \frac{2(1-\nu)N}{((1-\nu)n-u+1)((1-\nu)n-u)} \\
&= 2(1-\nu)N\left(\frac{1}{(1-\varepsilon_\nu)(1-\nu)n} - \frac{1}{(1-\nu)n}\right) \\
&= \frac{2N\varepsilon_\nu}{(1-\varepsilon_\nu)n}.
\end{aligned} \tag{S59}$$

Comparing this lower bound on $\mathbb{E}[T^{\,\mathrm{car}}]$ with the upper bound in Eq. (S56) gives

$$\frac{2N\varepsilon_\nu}{(1-\varepsilon_\nu)n} \leq \frac{\ln(2Ns)}{s}. \tag{S60}$$

This gives the upper bound on ε_ν in Eq. (S57). ■

Theorem 7 *Consider a sample of n individuals under a hard sweep with νn carriers ($\frac{1}{n} \leq \nu \leq \frac{n-1}{n}$). Assume strong selection ($Ns \gg 1$). In the time $[0, T^{\,car}]$, let the number of coalescent events among the $(1-\nu)n$ non-carrier haplotypes be $\varepsilon_\nu(1-\nu)n$. The expected 1-HAF score of a random carrier haplotype is*

$$\mathbb{E}[1\text{-}HAF^{car}] \approx \theta n \left(\frac{\nu+1}{2} - \frac{1}{(1-\varepsilon_\nu)(1-\nu)n+1}\right), \tag{S61}$$

while for a random non-carrier haplotype,

$$\mathbb{E}[1\text{-}HAF^{non}] \approx \theta n \left(\frac{1}{2} + \frac{1}{2n} - \frac{1 - \varepsilon_\nu (1-\nu)}{(1-\varepsilon_\nu)(1-\nu)n + 1} \right). \tag{S62}$$

Proof. Recall the decomposition of $\mathbb{E}[\text{1-HAF}]$ given in Eq. (S37):

$$\mathbb{E}[\text{1-HAF}] = \mathbb{E}[(\mathbf{w}^{\,\text{car}})^b \cdot \mathbf{h}^b] + \mathbb{E}[(\mathbf{w}^{\,\text{non}})^b \cdot \mathbf{h}^b] + \mathbb{E}[(\mathbf{w}^{\,\text{all}})^a \cdot \mathbf{h}^a]. \tag{S63}$$

The number of ancestors of non-carriers at time T^{car} is $m = (1-\varepsilon_\nu)(1-\nu)n\,.$

For a random carrier haplotype $\mathbf{h}$, we evaluate the first two terms in Eq. (S63) by using Eqs. (S38) and (S39), and bound the third term by using Lemma 5, to obtain:

$$0 \le \mathbb{E}[\text{1-HAF}^{\,\text{car}}] - \theta n \left(\frac{\nu+1}{2} - \frac{1}{(1-\varepsilon_\nu)(1-\nu)n+1} \right) \le \theta n \cdot \frac{\ln(2Ns)}{2Ns}.$$

For a random non-carrier haplotype $\mathbf{h}$, we use Eqs. (S40) and (S41) and Lemma 5:

$$0 \le \mathbb{E}[\text{1-HAF}^{\,\text{non}}] - \theta n \left(\frac{1}{2} + \frac{1}{2n} - \frac{1-\varepsilon_\nu(1-\nu)}{(1-\varepsilon_\nu)(1-\nu)n+1} \right) \le \theta n \cdot \frac{\ln(2Ns)}{2Ns}.$$

Ignoring the small term $\frac{\ln(2Ns)}{2Ns}$, we obtain the desired expressions (S61) and (S62). ■

We find the maximum of Eq. (S61) over $0 \le \nu \le 1$ using differentiation; the result is

$$\max_\nu \mathbb{E}[\text{1-HAF}^{\,\text{car}}] \approx \theta n \left(1 - \frac{1}{\sqrt{2(1-\varepsilon_\nu)n}} \right)^2 \approx \theta n. \tag{S64}$$

We simulated selective sweeps for a variety of parameters and compared their trajectories against these results. In S15 Fig, we compared the trajectories of both carriers and non-carriers in 500 selective sweeps for each pair (θ, n) with $\theta \in \{24, 48\}$, $n \in \{100, 200, 300, 400\}$, $s = 0.08$, and $N = 2000$. Over the course of a trajectory, the frequency ν of the favored allele varies from $\nu = 1/n$ (1 carrier) to $\nu = 1$ (fixation), but different trajectories will go through a different sequence of values of ν. We aligned the trajectories by the values of ν. For each ν, we separated carriers and non-carriers and averaged together (1-HAF)/$(n\theta)$ over the generation (if any) with allele frequency ν in each sweep. We plotted these averaged trajectories in green ($\theta = 24$) and pink ($\theta = 48$). We compared these against the expected value (Theorem 7). The expected value is bounded above in blue ($\varepsilon_\nu = 0$) and below in red ($\varepsilon_\nu = \varepsilon_\nu^{\max}$); see Lemma 6. These are bounds on the expected value, not on simulation means. Simulation means may range over the whole distribution, but tend to vary around the expected value.

In this paper, due to the run time for forward simulations, we used a relatively small value for population size ($N = 2000$); in reality, N is a lot larger (10000), so that ε_ν becomes smaller (see Lemma 6). Therefore, the upper and lower bounds in the proof of Theorem 7 move closer together, and we approximate $\varepsilon_\nu = 0$. To simplify the presentation in the main text, we set $\varepsilon_\nu = 0$, giving:

$$\mathbb{E}[\text{1-HAF}^{\,\text{car}}] \approx \theta n \left(\frac{\nu+1}{2} - \frac{1}{(1-\nu)n+1} \right),$$

$$\mathbb{E}[\text{1-HAF}^{\,\text{non}}] \approx \theta n \left(\frac{1}{2} + \frac{1}{2n} - \frac{1}{(1-\nu)n+1} \right),$$

$$\max_\nu \mathbb{E}[\text{1-HAF}^{\,\text{car}}] \approx \theta n \left(1 - \frac{1}{\sqrt{2n}} \right)^2 \approx \theta n.$$

References

1. Hudson RR. Gene genealogies and the coalescent process. In: Futuyma D, Antonovics J, editors. Oxford Surveys in Evolutionary Biology. Oxford: Oxford University Press; 1990. p. 1–44.

2. Kingman JFC. On the genealogy of large populations. Journal of Applied Probability. 1982;19:27–43.

3. Fu YX. Statistical properties of segregating sites. Theor Popul Biol. 1995 Oct;48(2):172–197.

4. Graham R, Knuth DE, Patashnik O. Concrete Mathematics: A Foundation for Computer Science. 2nd ed. Reading, Mass: Addison-Wesley; 1994.

5. Slatkin M, Hudson RR. Pairwise comparisons of mitochondrial DNA sequences in stable and exponentially growing populations. Genetics. 1991 Oct;129(2):555–562.

6. Hudson RR. Generating samples under a Wright-Fisher neutral model of genetic variation. Bioinformatics. 2002 Feb;18(2):337–338.

7. Ewing G, Hermisson J. MSMS: a coalescent simulation program including recombination, demographic structure and selection at a single locus. Bioinformatics. 2010 Aug;26(16):2064–2065.

8. Nordborg M. Coalescent Theory. In: Balding DJ, Bishop M, Cannings C, editors. Handbook of statistical genetics. 3rd ed. John Wiley & Sons, Ltd; 2008. p. 843–877.

9. Campbell RB. Coalescent size versus coalescent time with strong selection. Bull Math Biol. 2007 Oct;69(7):2249–2259.

10. Gravel S, Henn BM, Gutenkunst RN, Indap AR, Marth GT, Clark AG, et al. Demographic history and rare allele sharing among human populations. Proc Natl Acad Sci USA. 2011 Jul;108(29):11983–11988.

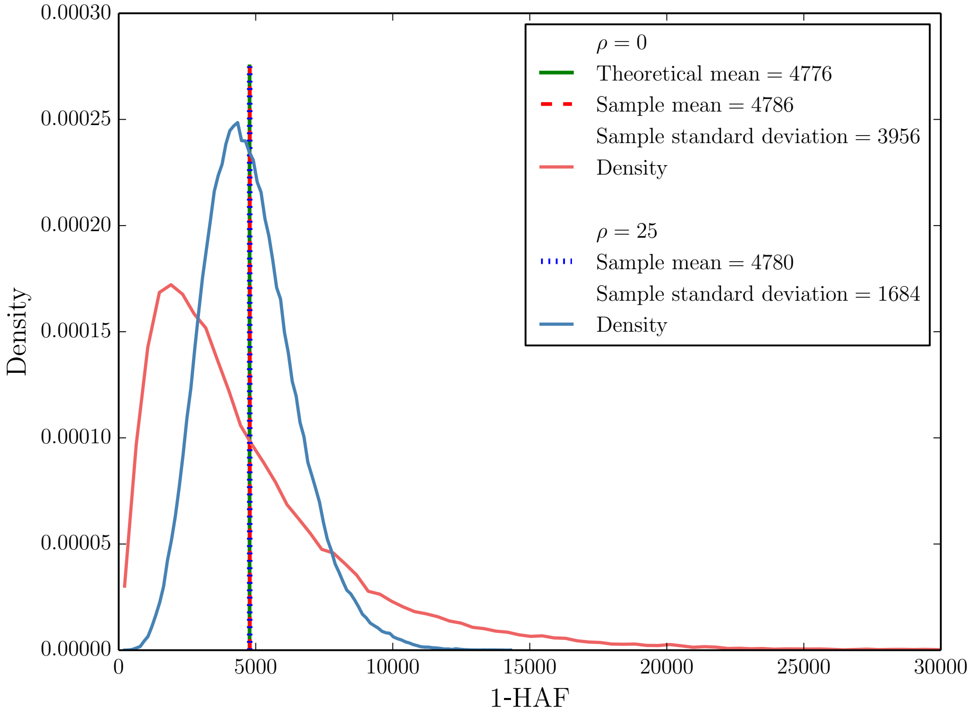

S1 Fig. HAF scores in neutrally evolving constant-sized populations. The distribution of 4×10^6 1-HAF scores aggregated from 20000 population samples (each of $n = 200$ haplotypes) simulated under a standard coalescent model without recombination. Plugging the simulation parameters $\theta = 48$, $n = 200$ into Eqs. (3) or (7) give an expected 1-HAF score of 4776. The observed mean 1-HAF score is 4786 ± 3956 with no recombination ($\rho = 0$), and 4780 ± 1684 with $\rho = 25$ (blue line).

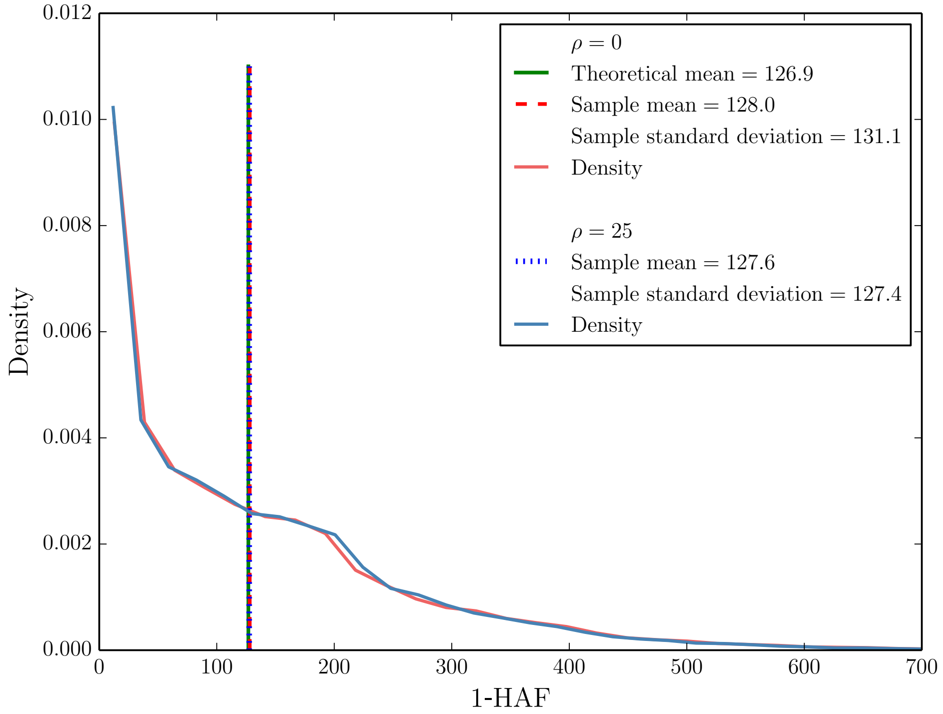

S2 Fig. HAF scores in neutrally evolving exponentially growing populations. The distribution of 4×10^6 1-HAF scores aggregated from 20000 population samples (each of $n = 200$ haplotypes) simulated under a coalescent model of exponential growth without recombination. Computing the conditional expectation as described in Eq. (12) with the simulation parameters ($\theta = 48$, $n = 200$, $\alpha = 80$) gives 126.9. The observed mean 1-HAF score is 128.0 with $\rho = 0$ (red line), and 127.4 with $\rho = 25$ (blue line).

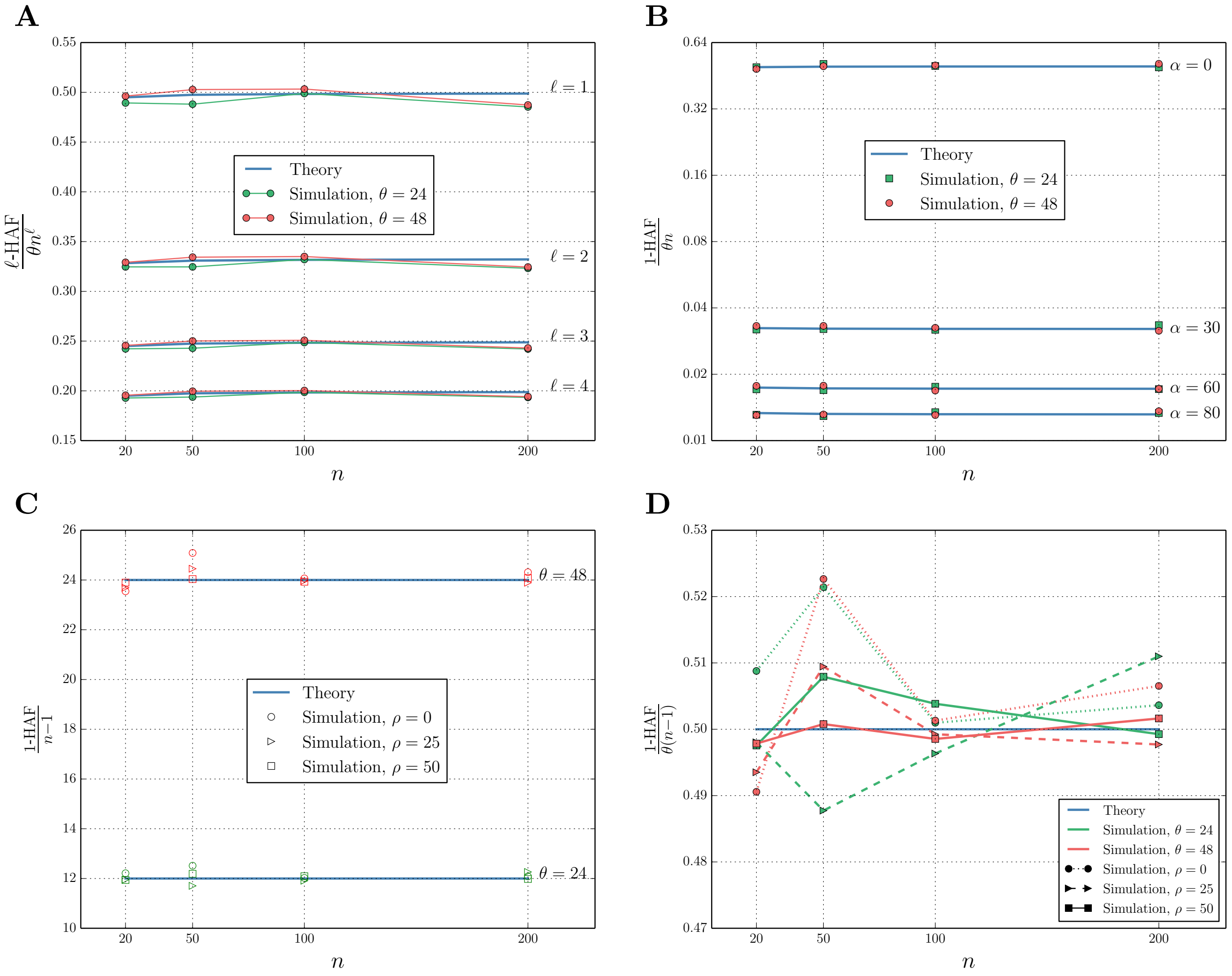

S3 Fig. HAF scores for a range of simulation parameters. Each empirical test is the average of 1000 trials. (A) Empirical mean and theoretical expected ℓ-HAF scores for a fixed size population ($\ell \in \{1, 2, 3, 4\}$, $\theta \in \{24, 48\}$, $\rho = 0$). (B) Empirical mean and theoretical expected 1-HAF scores for an exponentially growing population ($\alpha \in \{0, 30, 60, 80\}$, $\theta \in \{24, 48\}$, $\rho = 0$). (C) Theoretical expected 1-HAF scores (computed assuming $\rho = 0$) compared against empirical means of 1-HAF scores from samples with different recombination rates ($\rho \in \{0, 25, 50\}$, $\theta \in \{24, 48\}$). (D) Interestingly, higher recombination rates reduce the variance in 1-HAF estimates. In the three green curves for $\theta = 24$ (and in the three red curves for $\theta = 48$), the variation from the expected value (blue) decreases as ρ increases. Rate $\rho = 0$ (dotted) has the most variation; $\rho = 25$ (dashed) has less; and $\rho = 50$ (solid) has the least. The theoretical values are based on (A) Eqs. (3) and (S22), (B) Eq. (12), and (C,D) Eq. (4).

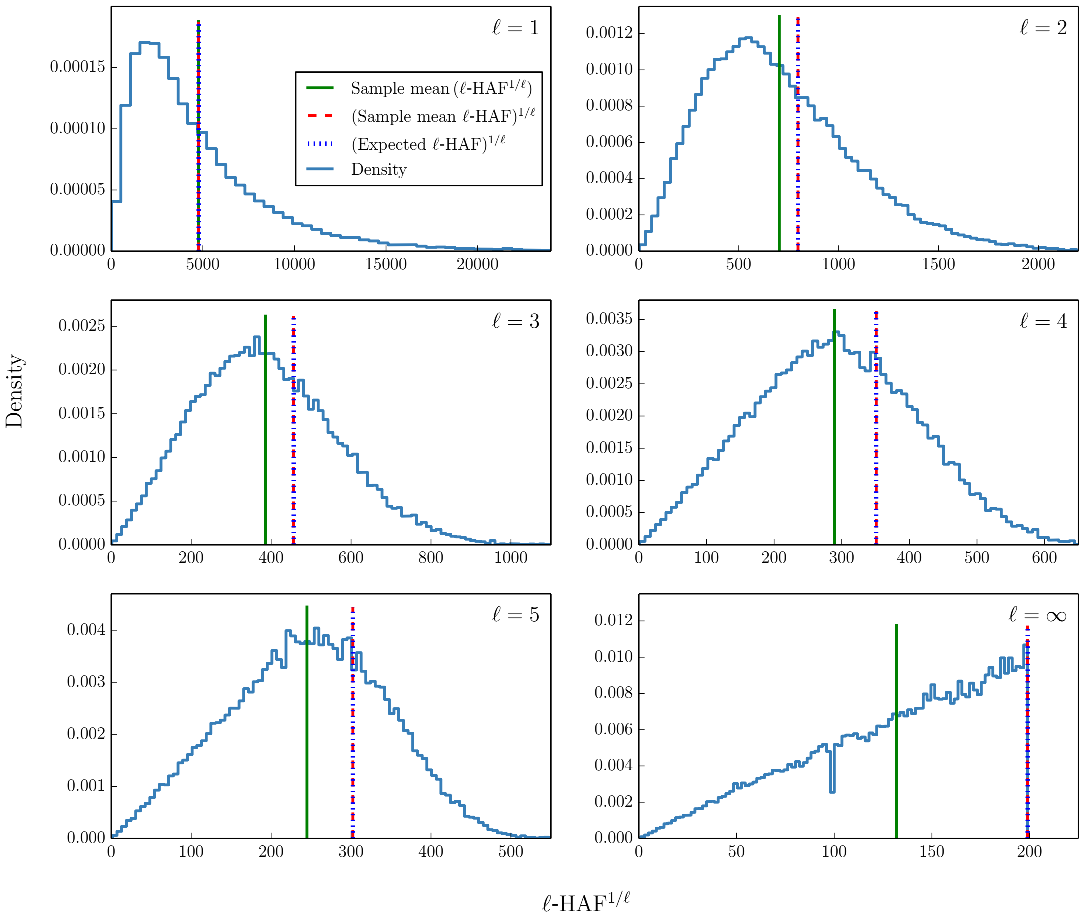

S4 Fig. Distribution of normalized ℓ-HAF scores (ℓ-HAF$^{1/\ell}$). Results are based on simulated samples of size $n = 200$ drawn from a larger population size of neutrally evolving haploid population with $N = 20000$ ($\theta = 48$, $\rho = 0$, $\alpha = 0$). The green line marks the sample mean of the ℓth root of ℓ-HAF, while the red dashed line marks the ℓth root of the sample mean of ℓ-HAF. The latter matches the blue dotted line, which marks the theoretically computed value of $(\mathbb{E}[\ell\text{-HAF}])^{1/\ell}$, using Eq. (S22). As ℓ increases, the high frequency mutations dominate the normalized ℓ-HAF score. The distribution becomes more left-skewed and has generally smaller values (upper bound of range approaching $n - 1$), with reduced variance.

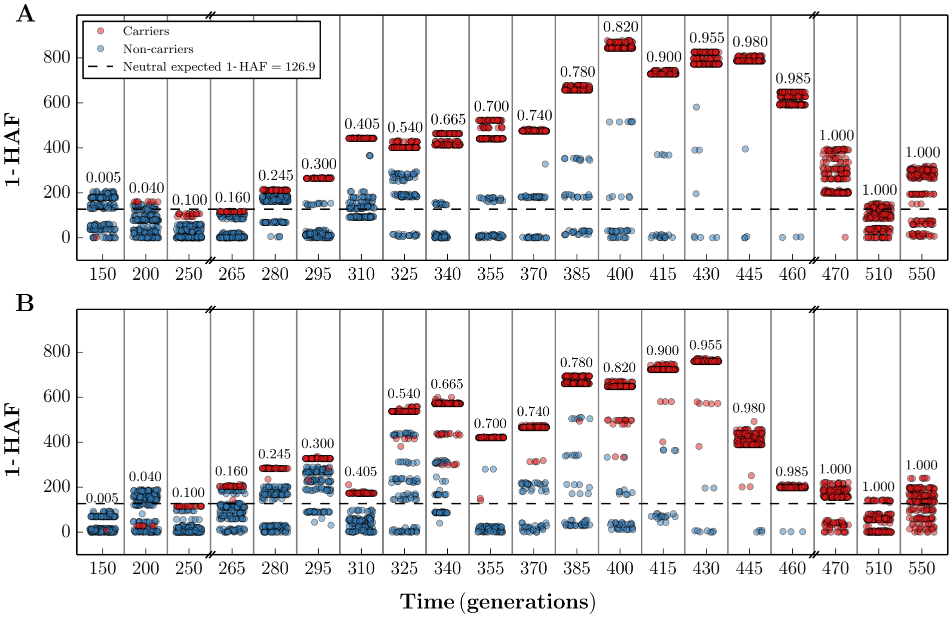

S5 Fig. Schematic of HAF score dynamics in an exponentially growing population with current population size $N = 20000$, population-scaled growth rate $\alpha = 80$, and population-scaled mutation rate $\theta = 48$. The population is under selection with $s = 0.05$. See Fig 2 for an explanation of the conventions used.

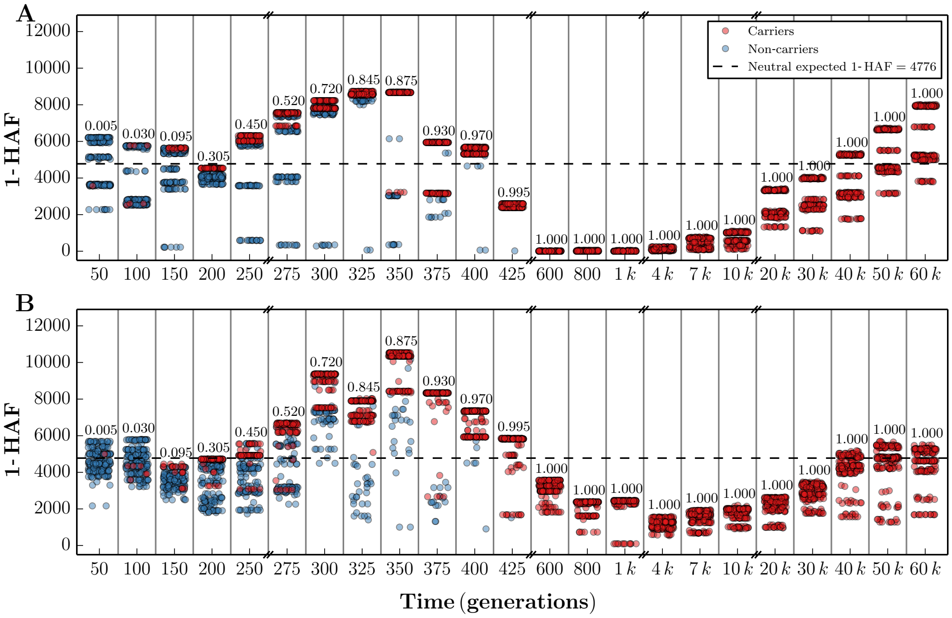

S6 Fig. Schematic of HAF score dynamics in a population undergoing a soft sweep due to standing variation with $\nu_0 = 0.002$. Samples were simulated with $\theta = 48$, $n = 200$, $s = 0.05$, and $\rho \in \{0, 25\}$. See Fig 2 for an explanation of the conventions used.

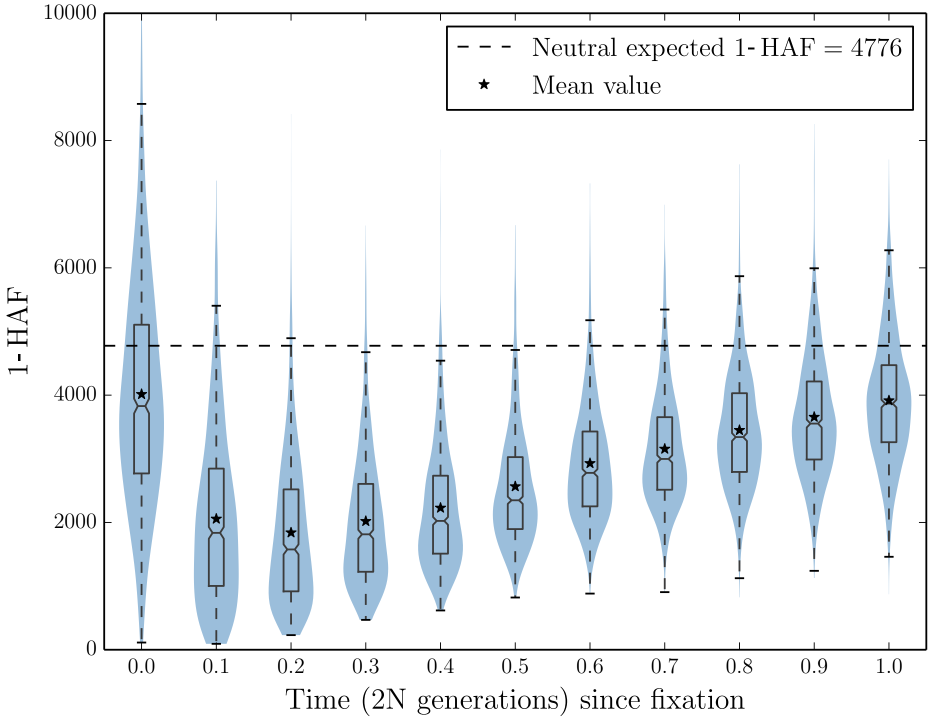

S7 Fig. Recovery of HAF scores after a selective sweep. Each violin shows the Gaussian kernel density estimation (KDE) of 1-HAF scores in populations sampled at regular time intervals following the fixation of a selective sweep. All individuals at this stage are carriers of the favored allele. A standard box plot is overlaid on each violin. The horizontal dotted line represents the neutral expected value. At each time point, HAF scores were computed from 1000 simulations with *msms* [7], each with $n = 200$ haplotypes undergoing a hard sweep, with parameters $N = 20000$, $\theta = 48$, $\rho = 25$, $n = 200$. At each time point, box plots marking 25th, 50th, and 75th percentiles were computed for the 1000×200 HAF scores, with an asterisk marking the mean.

Empirical validation of PreCIOSS

We tested the performance of PreCIOSS for different population genetics parameters provided in S1 Table. For each choice of parameters, we applied PreCIOSS to data from 200 samples.

Parameters	s	θ	n	ρ
A	0.01	48	200	25
B	0.01	48	**100**	25
C	0.01	48	**50**	25
D	0.01	48	**20**	25
E	**0.005**	48	200	25
F	0.01	**24**	200	25
G	0.01	48	200	**0**
H	0.01	48	200	**50**

S1 Table. Simulation parameter sets used for generating S8 Fig. In simulations B through E, we changed one parameter (in boldface) at a time vs. simulation A.

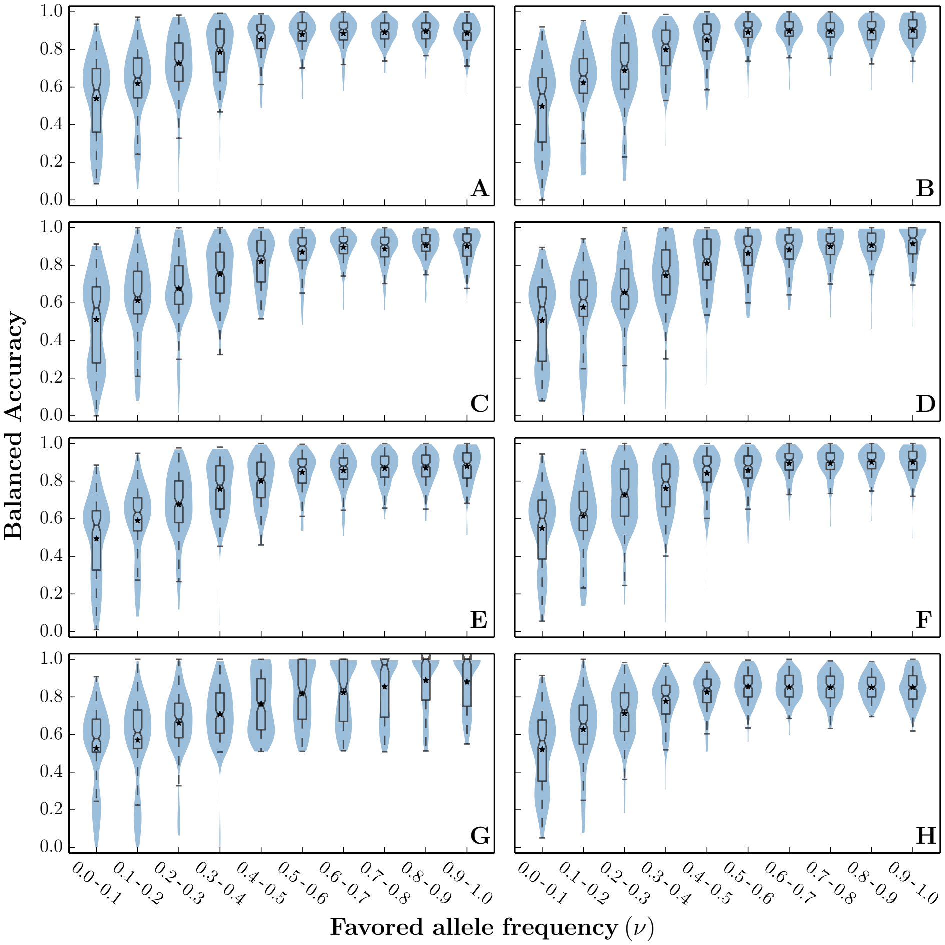

S8 Fig. Predicting carriers of hard sweeps. Balanced accuracy of PreCIOSS in populations undergoing hard sweeps. Balanced accuracy is shown for each allele frequency bin as a standard box plot computed over 200 samples for each frequency bin, and each parameter set in S1 Table.

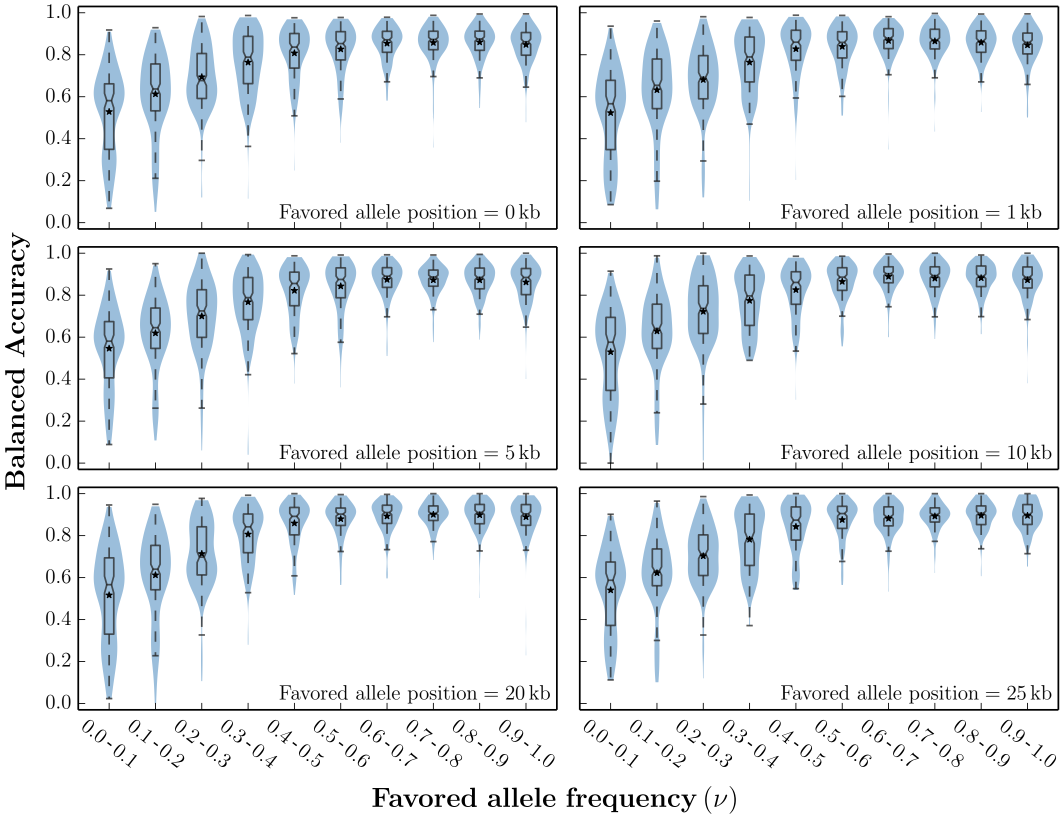

S9 Fig. Balanced accuracy with different positions of favored allele. In each panel, 200 samples were simulated ($N = 20000$, $n = 200$, $\theta = 48$, $\rho = 25$) while undergoing a hard sweep ($s = 0.01$) in a 50 kb window. Each panel shows balanced accuracy for a different position of the favored allele within the window, as the position varies from 0 to 25 kb.

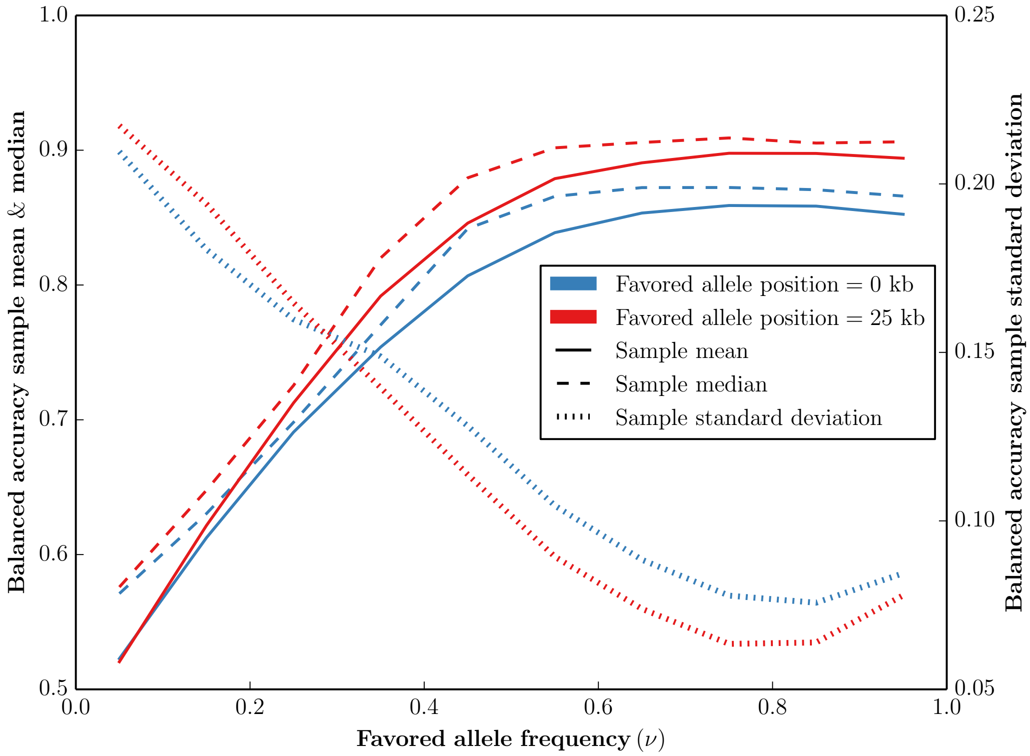

S10 Fig. Balanced accuracy variation with different positions of favored allele: summary statistics. In each case, 5000 samples were simulated ($N = 20000$, $n = 200$, $\theta = 48$, $\rho = 25$) while undergoing a hard sweep ($s = 0.01$) in a 50 kb window. The mean, median and standard deviation of balanced accuracy of PreCIOSS was measured with the favored allele at the start of the window (0 kb, in blue) and at the middle of the window (25 kb, in red).

PreCIOSS performance on demographic models

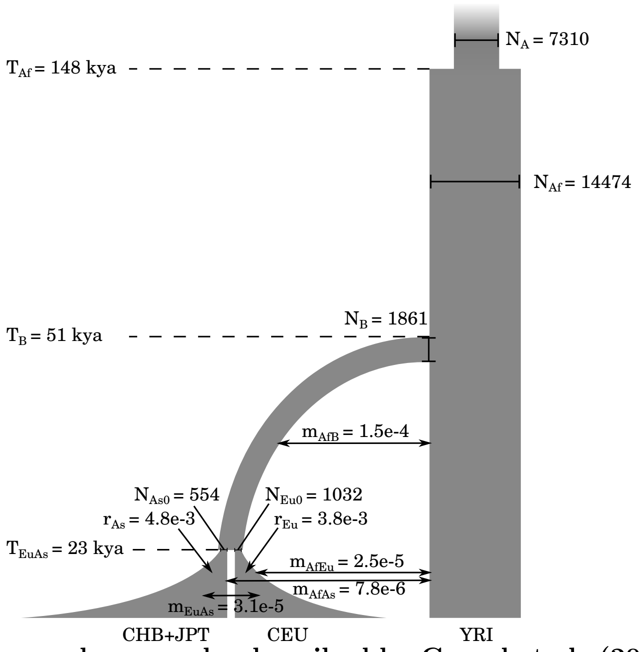

S11 Fig. A model of human demography described by Gravel et al. (2011) [10, Fig 4, Table 2]. The model assumes an out-of-Africa split at time T_B, with a bottleneck that reduced the effective population from N_{Af} to N_B, allowing for migrations at rate $m_{\text{Af-B}}$. The African population stays constant at N_{Af} up to the present generation. The model assumes a second split between European and Asian populations at time T_{EuAs}, with a bottleneck reducing the Asian and European populations to N_{As0} and N_{Eu0} respectively. The bottleneck was followed by exponential growth at rates r_{As} and r_{Eu}, as well as migrations among all three sub-populations, leading to current populations from which Asian (CHB+JPT), European (CEU), and Africans (YRI) individuals were sampled.

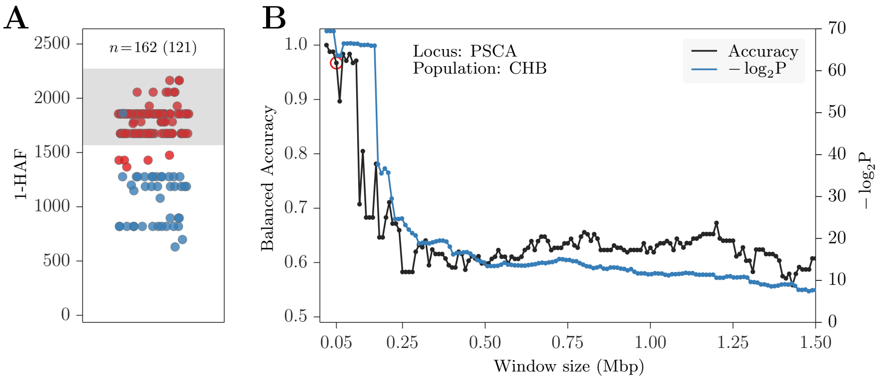

S12 Fig. Predicting carriers of the PSCA sweep in CHB. (A) Haplotype 1-HAF scores in a 50 kb window centered at the favored site. (B) Balanced classification accuracy (black) and $-\log_2(P)$ values (blue) as function of window size around the favored allele. P-values are for Wilcoxon rank sum tests rejecting the null hypothesis of identically distributed 1-HAF scores among carriers and non-carriers. The red circle indicates the balanced accuracy obtained for the 50 kb window shown on the left. As with the YRI population, we achieve high classification accuracy when considering ∼100 kb window centered at the favored allele. But unlike in YRI, we see a sharp decline in both accuracy and $-\log_2(P)$ values beginning at larger distances from the favored allele. See Fig 7 for further details on the conventions used.

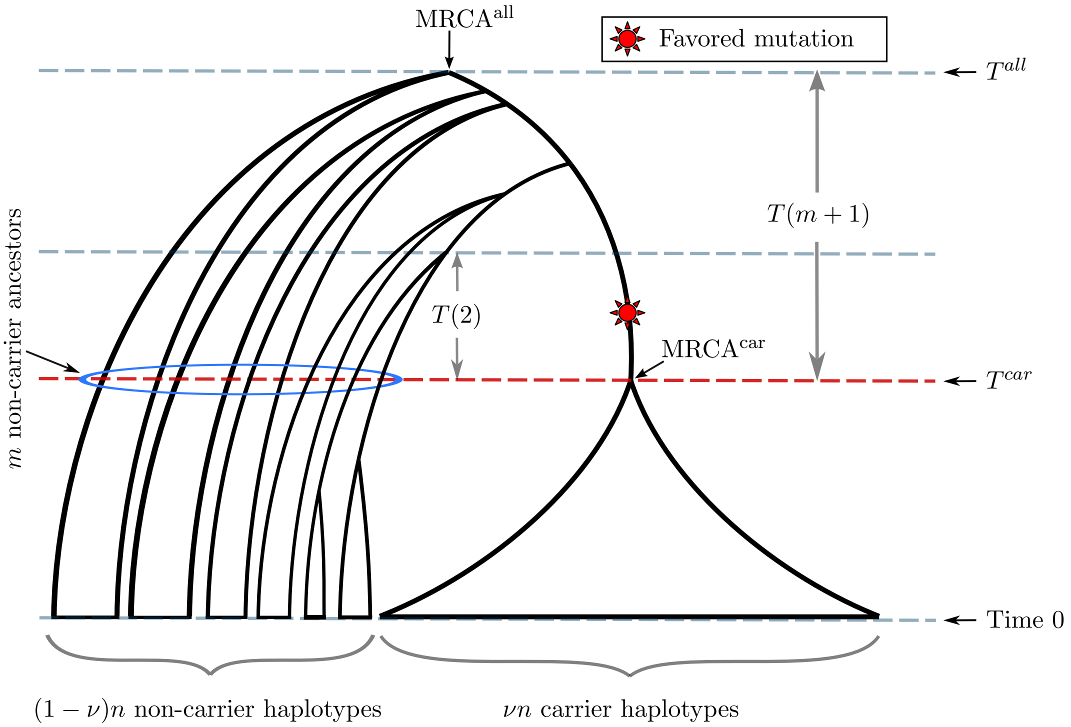

S13 Fig. The coalescence of a sample of n individuals to their most recent common ancestor MRCA^{all}, during a hard sweep. We assume that the current time has νn carriers of the favored allele. These coalesce to MRCA^{car} in T^{car} generations. From that point, the coalescence to MRCA^{all} is governed by neutral coalescent theory. $T(k)$ is time to MRCA of k randomly chosen haplotypes in a neutrally evolving population.

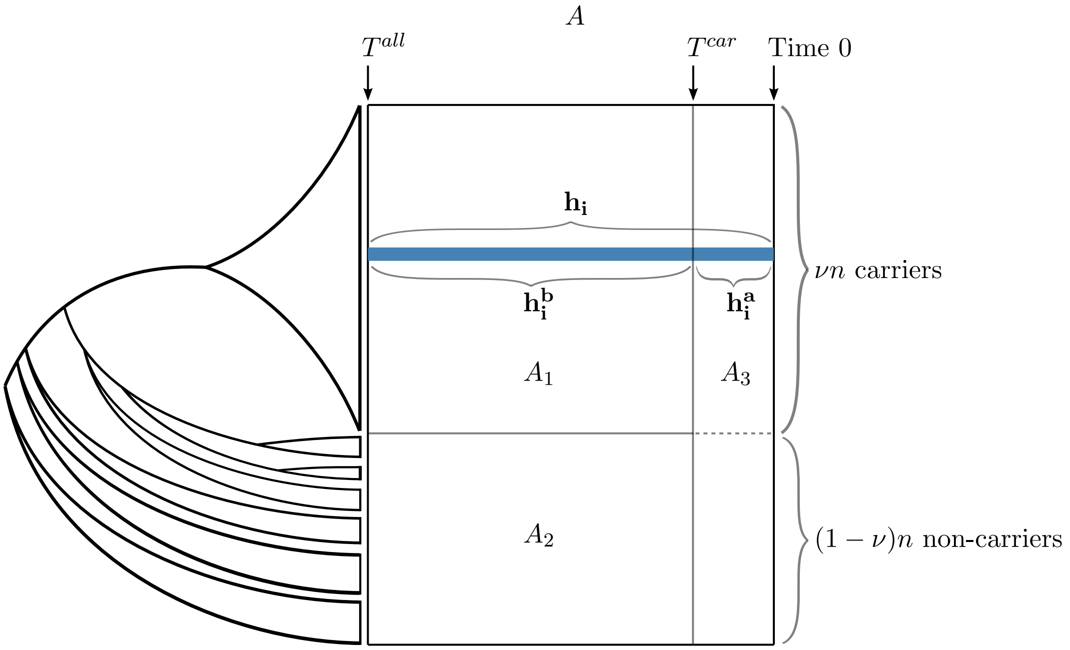

S14 Fig. Partitioning the SNP matrix A of a sample of n individuals.

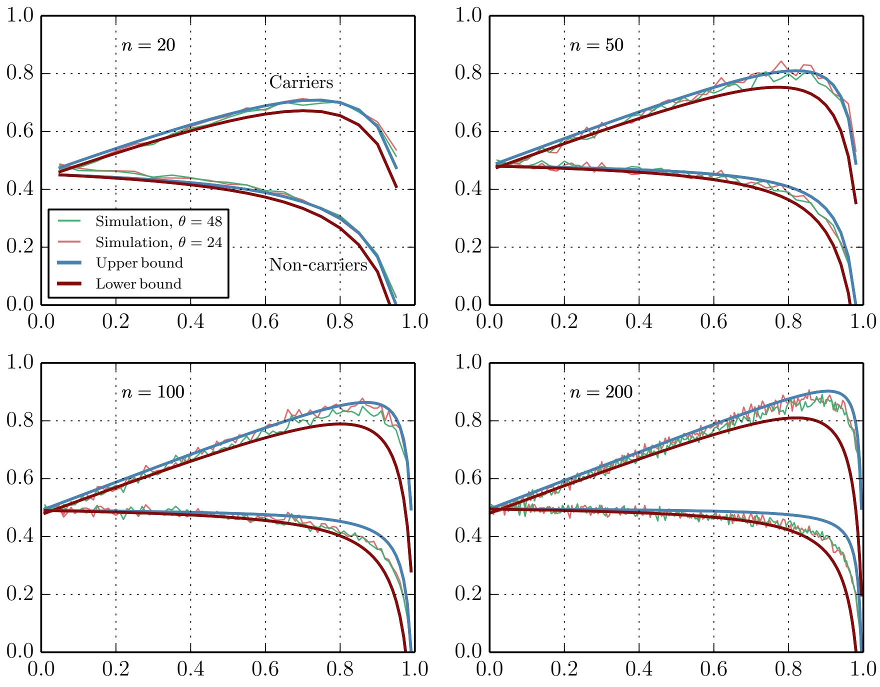

S15 Fig. Dynamics of expected 1-HAF score during a selective sweep. For each (θ, n, ν) with $\theta \in \{24, 48\}$, $n \in \{100, 200, 300, 400\}$, $\nu \in \{\frac{1}{n}, \frac{2}{n}, \ldots, \frac{n-1}{n}\}$, $s = 0.08$, and $N = 2000$, we did 1500 trials. We plotted the mean value of (1-HAF)/(θn) as a function of ν, for both carriers and non-carriers, and compared against the theoretical expected value. The expected value of (1-HAF)/$(n\theta)$ lies somewhere between the blue and red curves. The mean values may range over the whole distribution (and are not constrained by the blue and red curves) but tend to vary around the expected value.
